# Supplementary figures and images for: KLF7 is a general inducer of human pluripotency
Source: EMBO Rep. 2025 Oct 15;26(22):5372–96. doi: 10.1038/s44319-025-00595-2 (PMC12635360; doi:10.1038/s44319-025-00595-2)

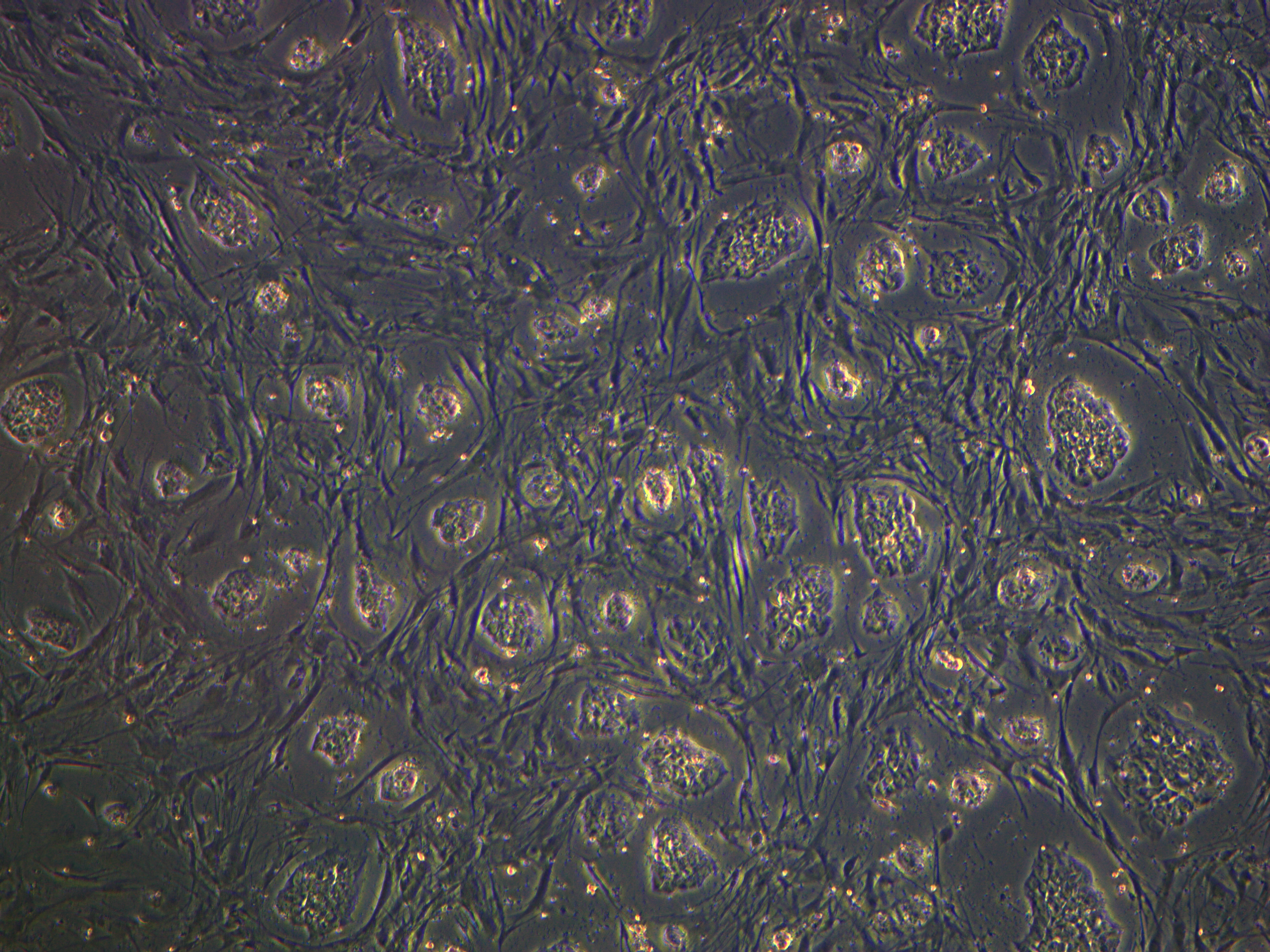

Supplement: Supplementary file 5 — Source data Fig. 3 [file 44319_2025_595_MOESM5_ESM.zip › Figure 3/3B/EMPTY-iPSC_D0.tiff]

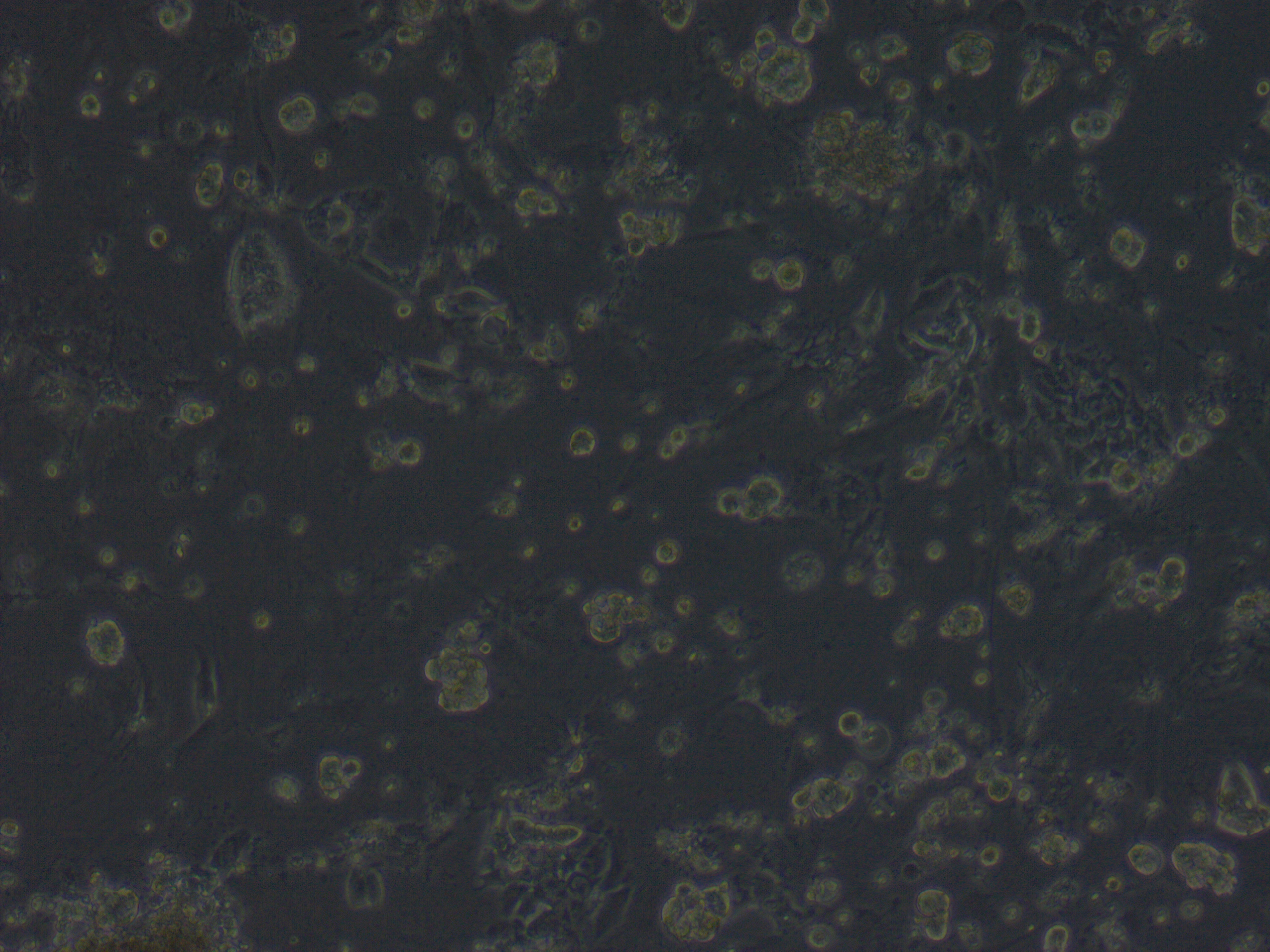

Supplement: Supplementary file 5 — Source data Fig. 3 [file 44319_2025_595_MOESM5_ESM.zip › Figure 3/3B/EMPTY-iPSC_D12_BF.tiff]

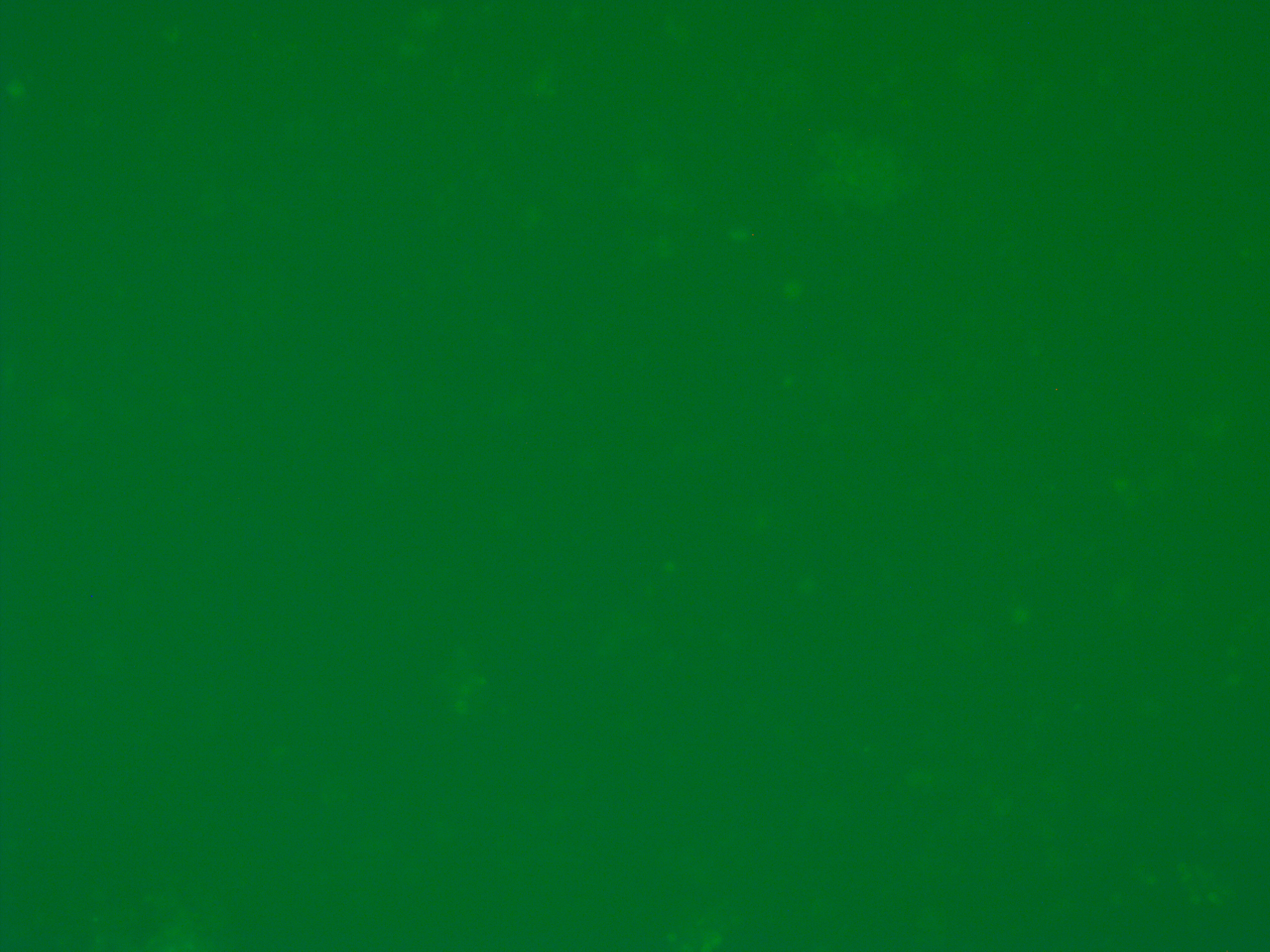

Supplement: Supplementary file 5 — Source data Fig. 3 [file 44319_2025_595_MOESM5_ESM.zip › Figure 3/3B/EMPTY-iPSC_D12_EOS.tiff]

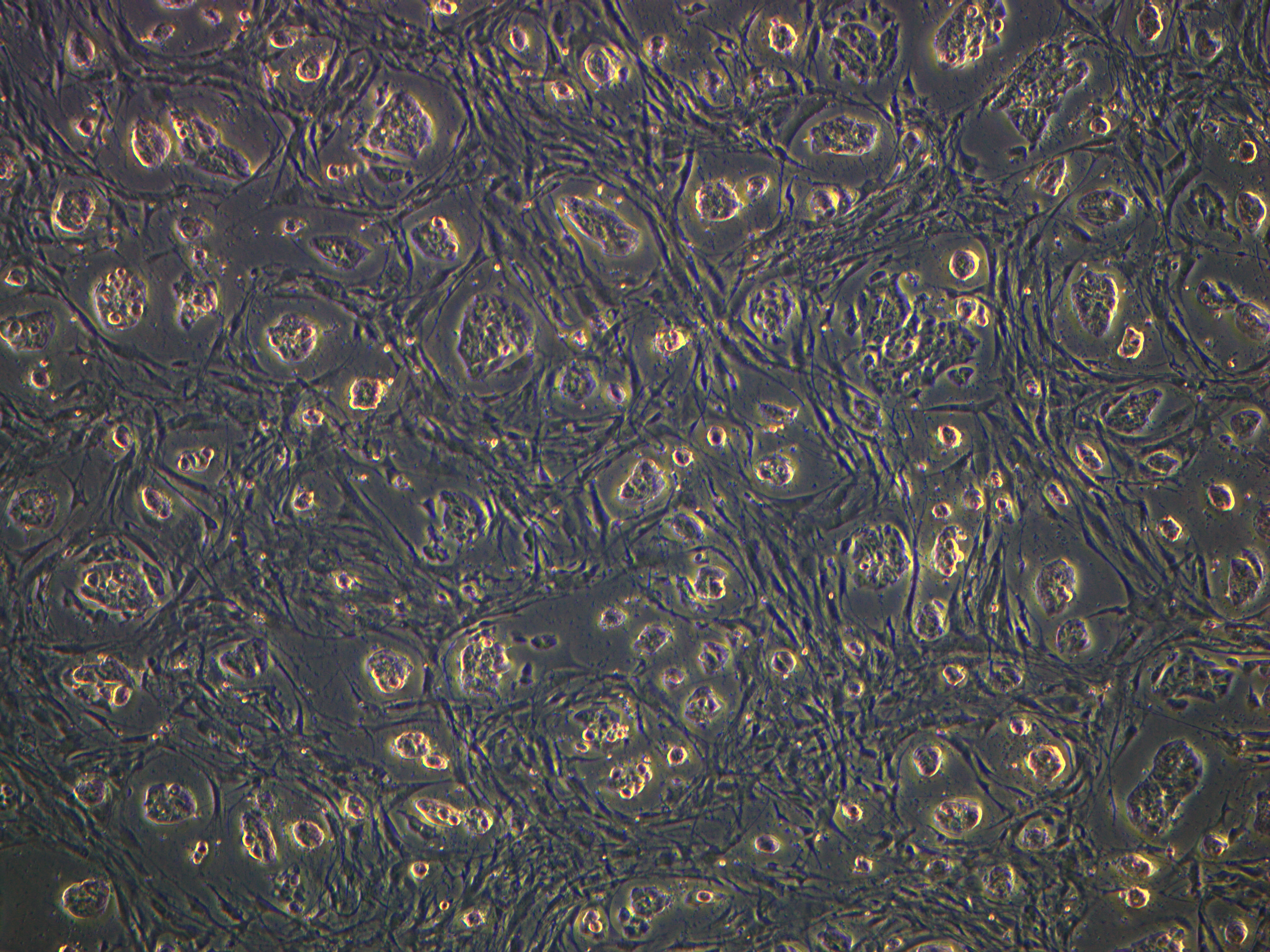

Supplement: Supplementary file 5 — Source data Fig. 3 [file 44319_2025_595_MOESM5_ESM.zip › Figure 3/3B/KLF7-iPSC_D0.tiff]

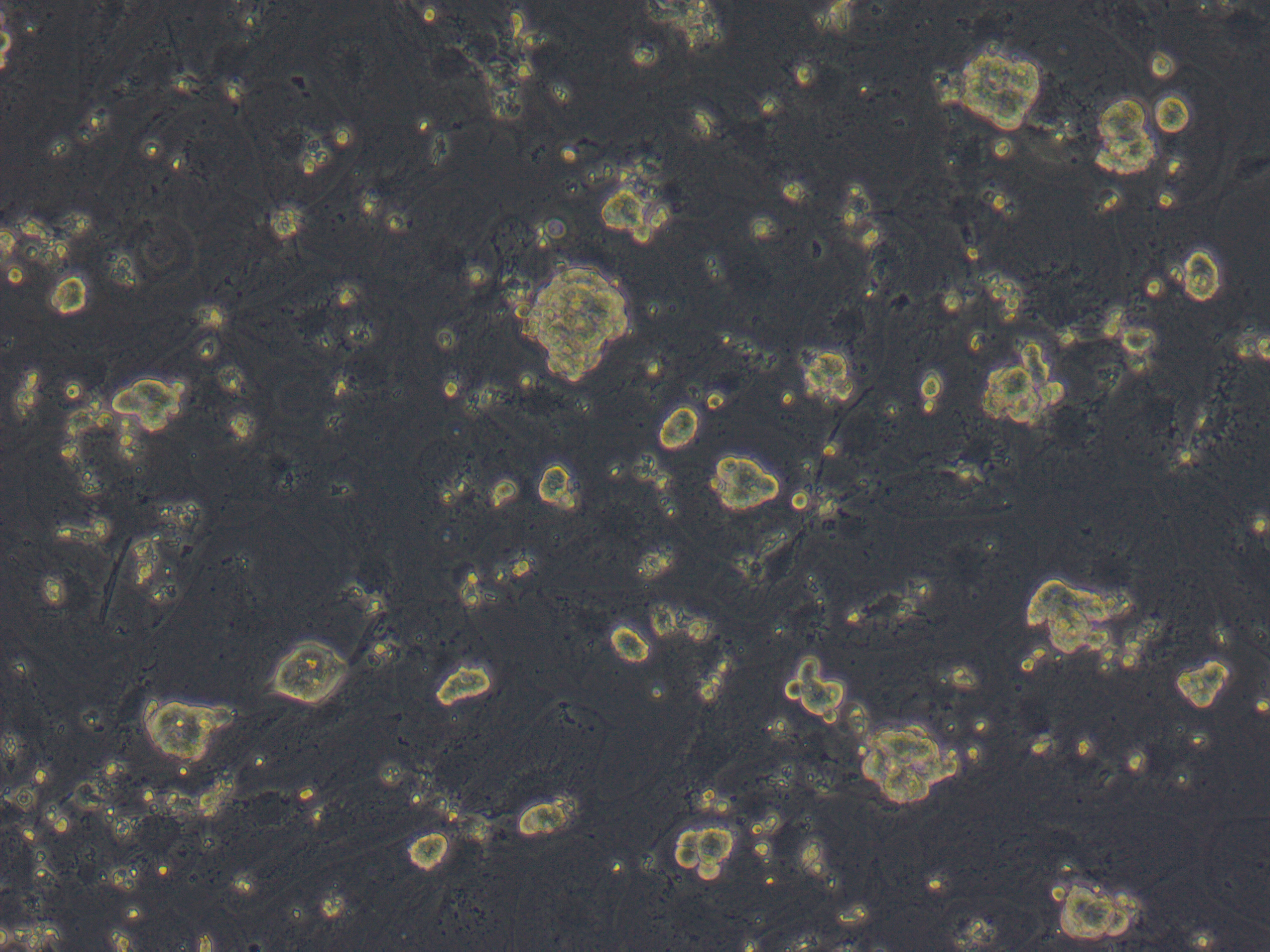

Supplement: Supplementary file 5 — Source data Fig. 3 [file 44319_2025_595_MOESM5_ESM.zip › Figure 3/3B/KLF7-iPSC_D12_BF.tiff]

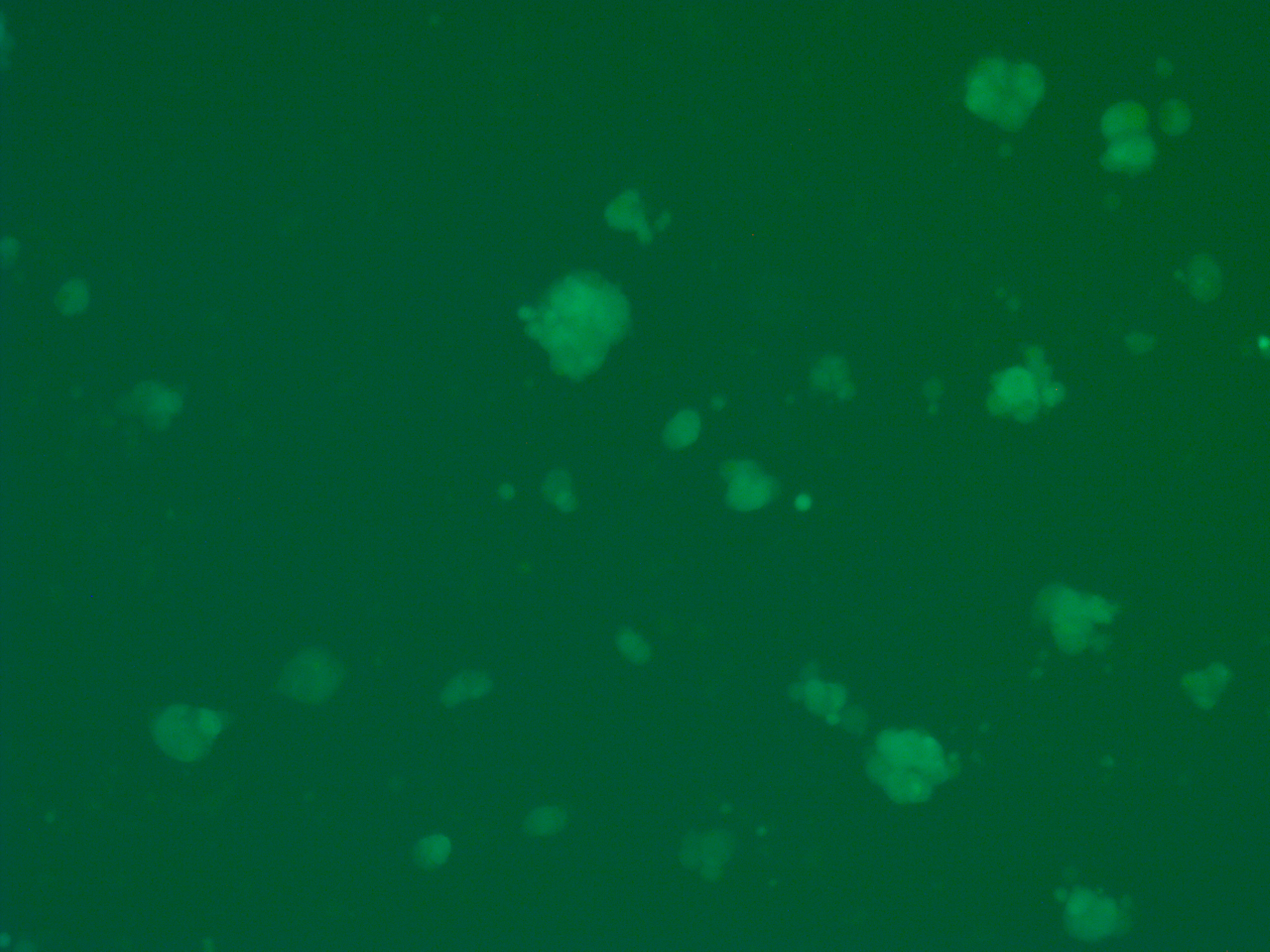

Supplement: Supplementary file 5 — Source data Fig. 3 [file 44319_2025_595_MOESM5_ESM.zip › Figure 3/3B/KLF7-iPSC_D12_EOS.tiff]

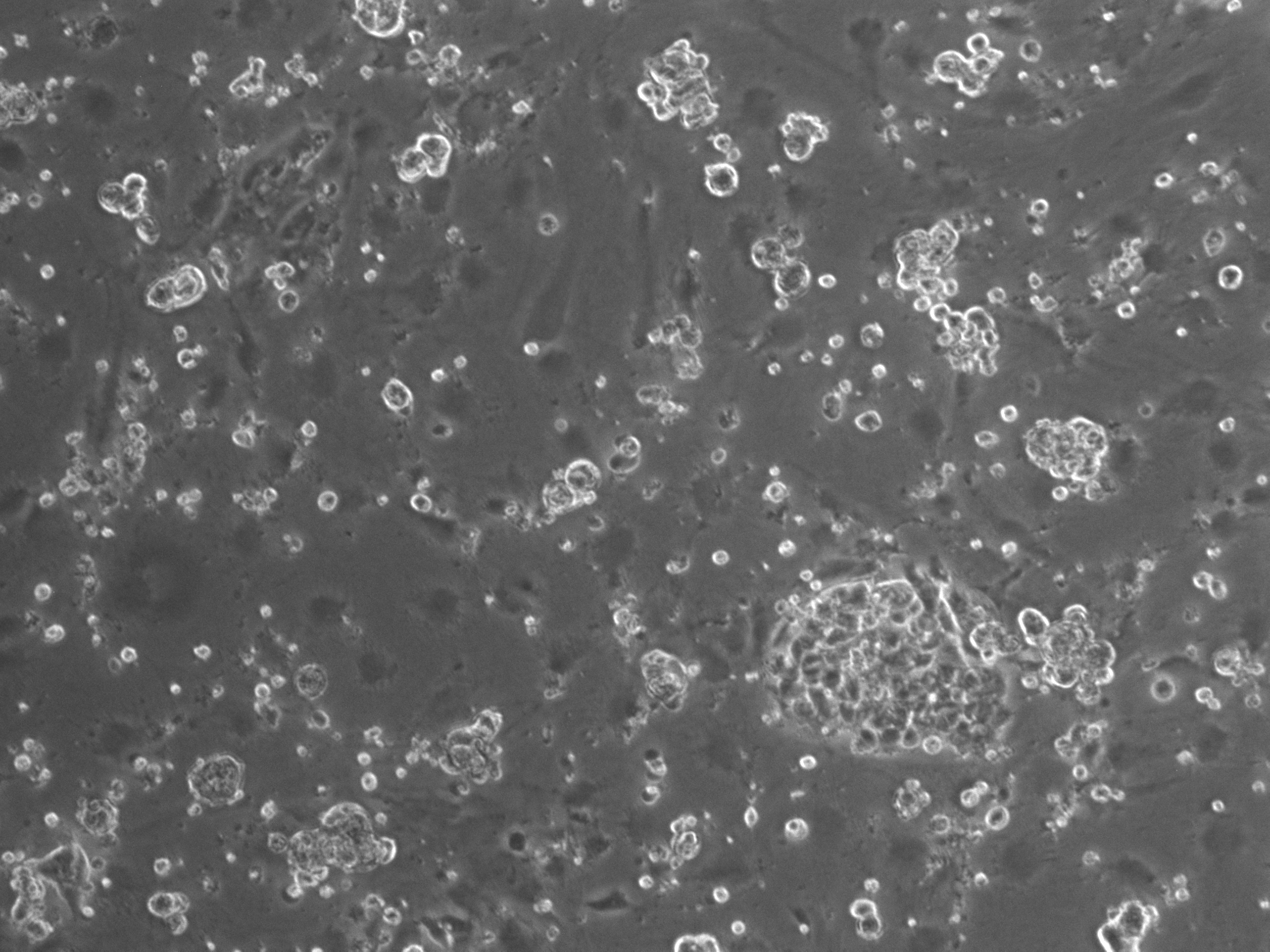

Supplement: Supplementary file 5 — Source data Fig. 3 [file 44319_2025_595_MOESM5_ESM.zip › Figure 3/3F/exp1 resetting KLF7 vs KLF4_EMPTY-iPSC_D14.tiff]

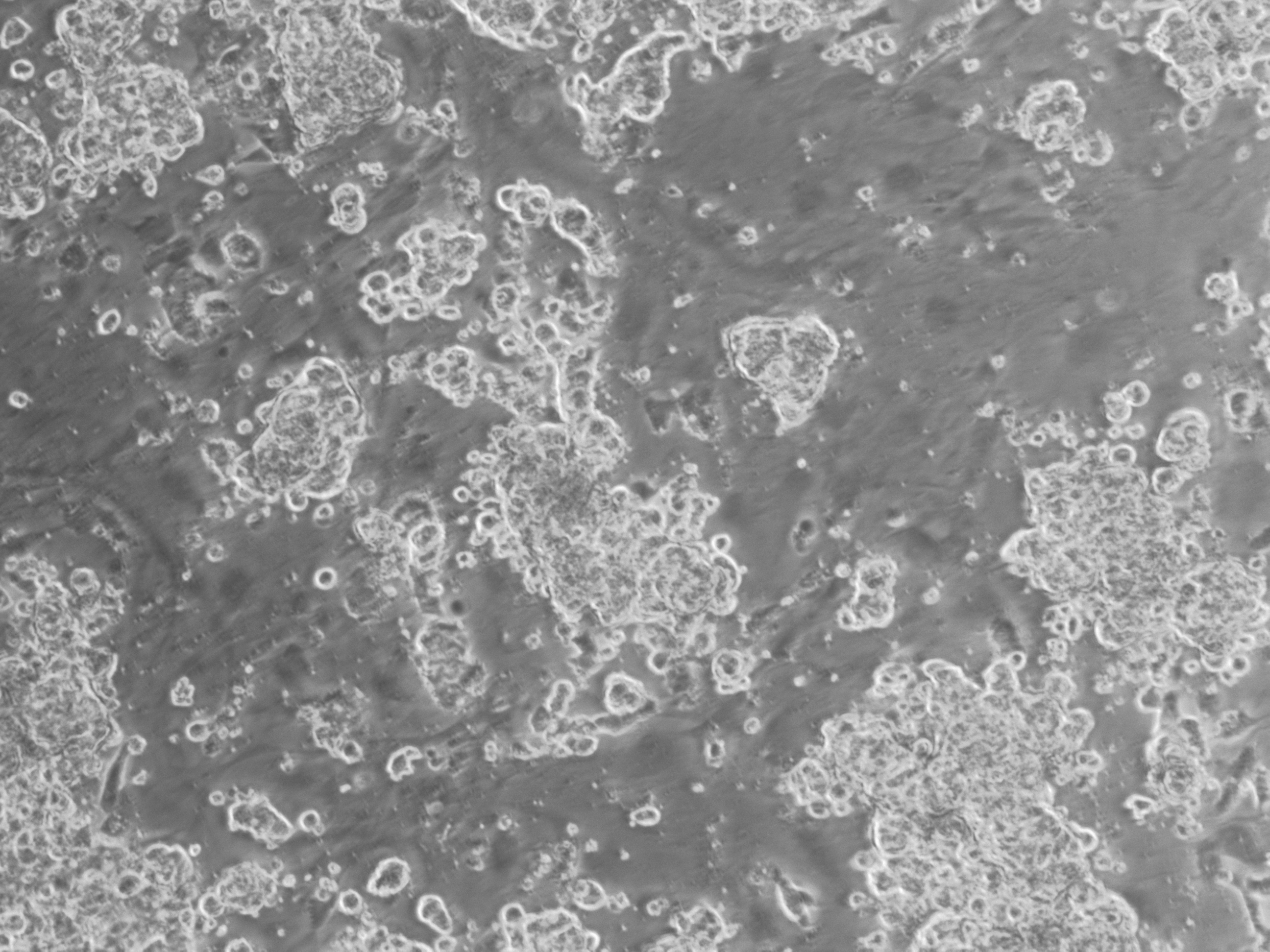

Supplement: Supplementary file 5 — Source data Fig. 3 [file 44319_2025_595_MOESM5_ESM.zip › Figure 3/3F/exp1 resetting KLF7 vs KLF4_EMPTY-iPSC_D8.tiff]

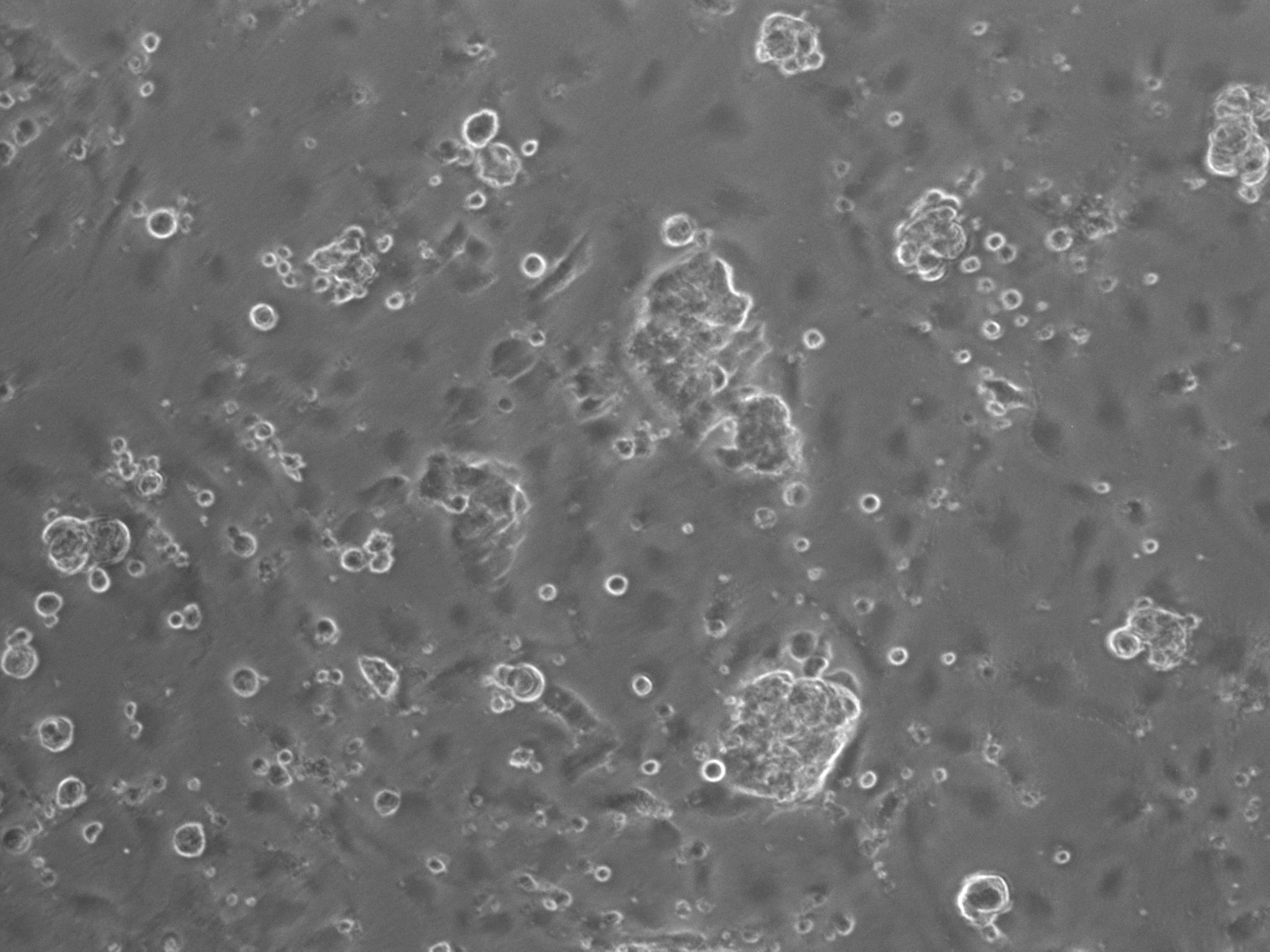

Supplement: Supplementary file 5 — Source data Fig. 3 [file 44319_2025_595_MOESM5_ESM.zip › Figure 3/3F/exp1 resetting KLF7 vs KLF4_KLF4-iPSC_D14.tiff]

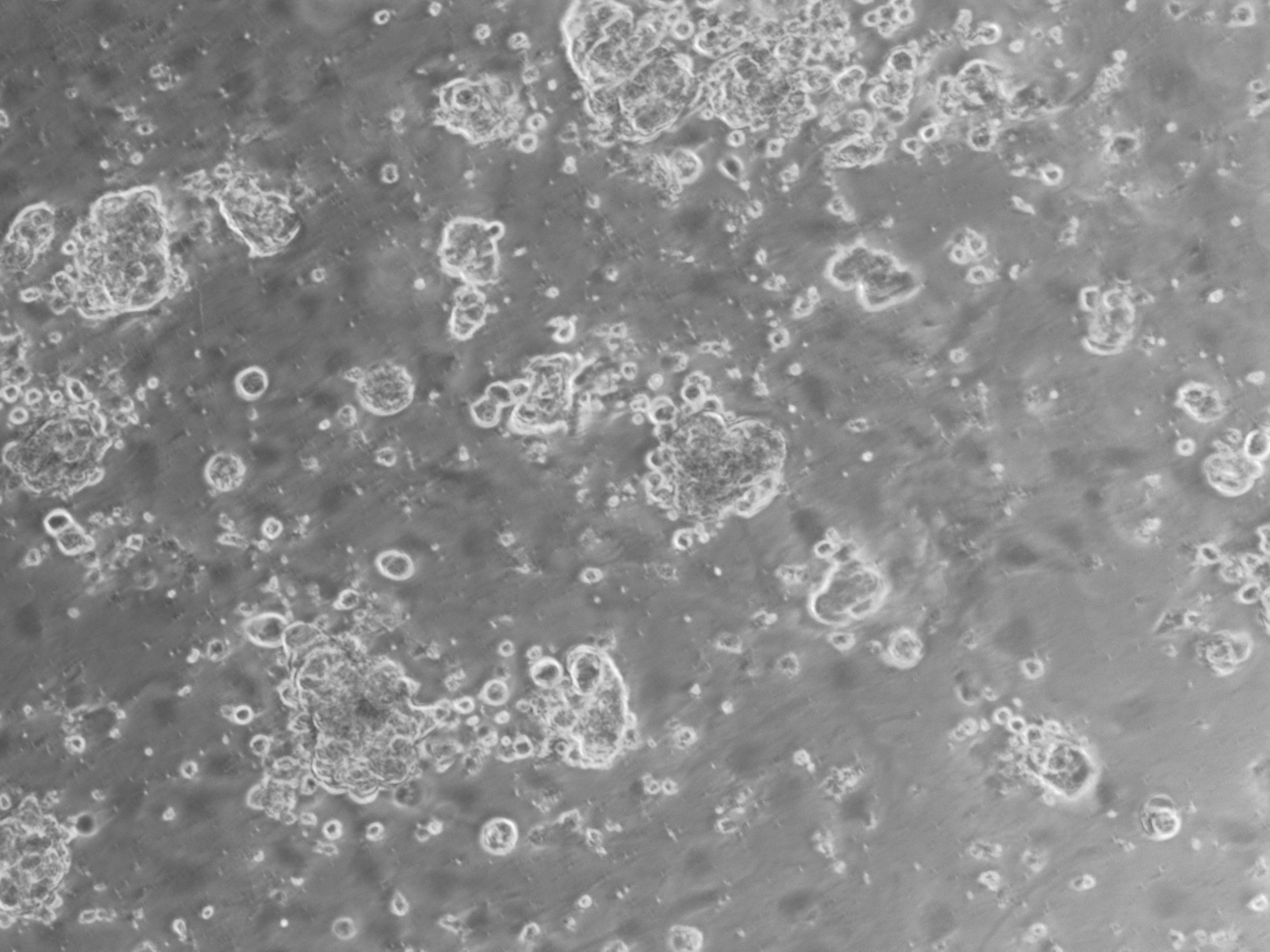

Supplement: Supplementary file 5 — Source data Fig. 3 [file 44319_2025_595_MOESM5_ESM.zip › Figure 3/3F/exp1 resetting KLF7 vs KLF4_KLF4-iPSC_D8.tiff]

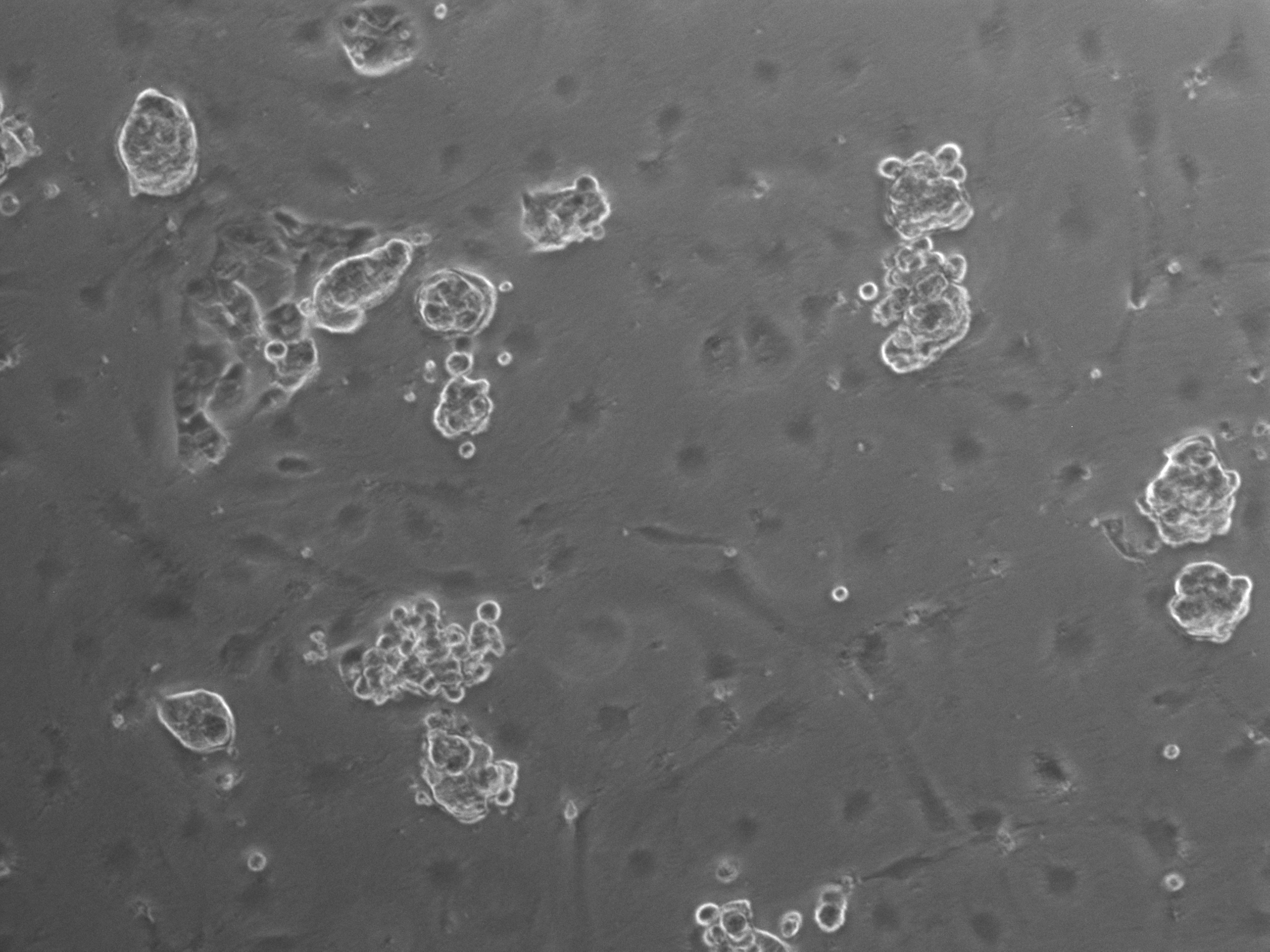

Supplement: Supplementary file 5 — Source data Fig. 3 [file 44319_2025_595_MOESM5_ESM.zip › Figure 3/3F/exp1 resetting KLF7 vs KLF4_KLF7-iPSC_D14.tiff]

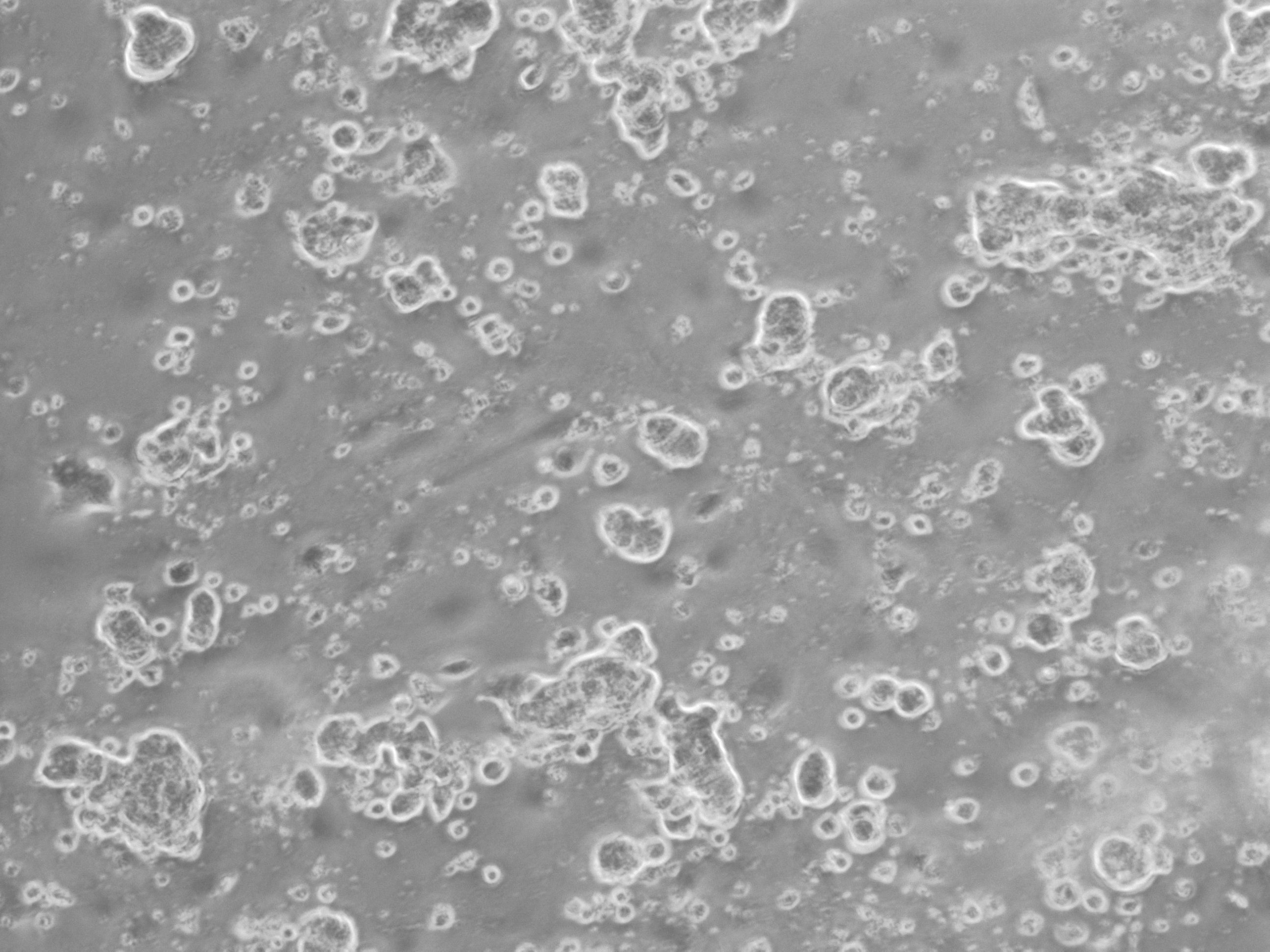

Supplement: Supplementary file 5 — Source data Fig. 3 [file 44319_2025_595_MOESM5_ESM.zip › Figure 3/3F/exp1 resetting KLF7 vs KLF4_KLF7-iPSC_D8.tiff]

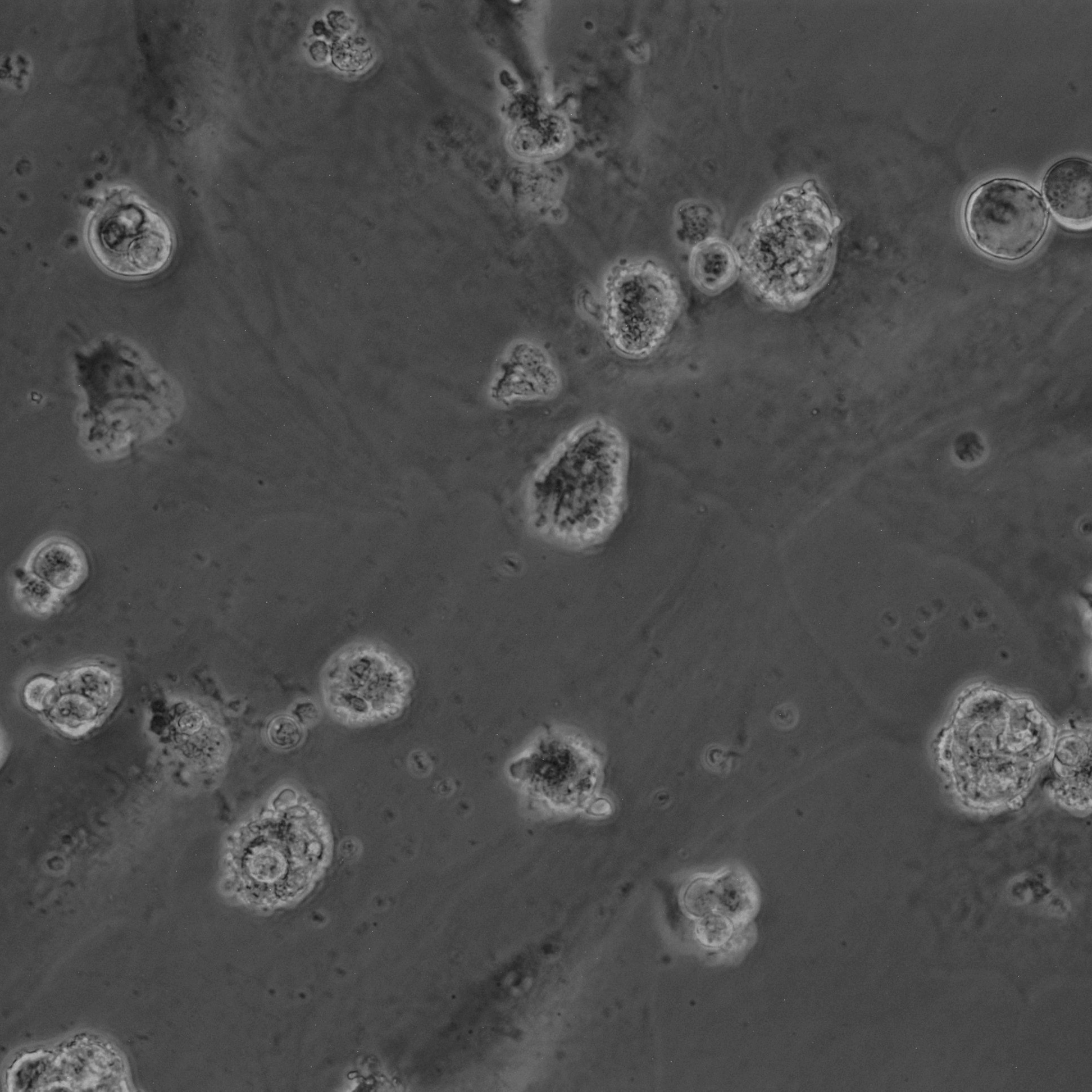

Supplement: Supplementary file 6 — Source data Fig. 4 [file 44319_2025_595_MOESM6_ESM.zip › Figure 4/4D/Control gRNA_Induced_D14_20x.jpg]

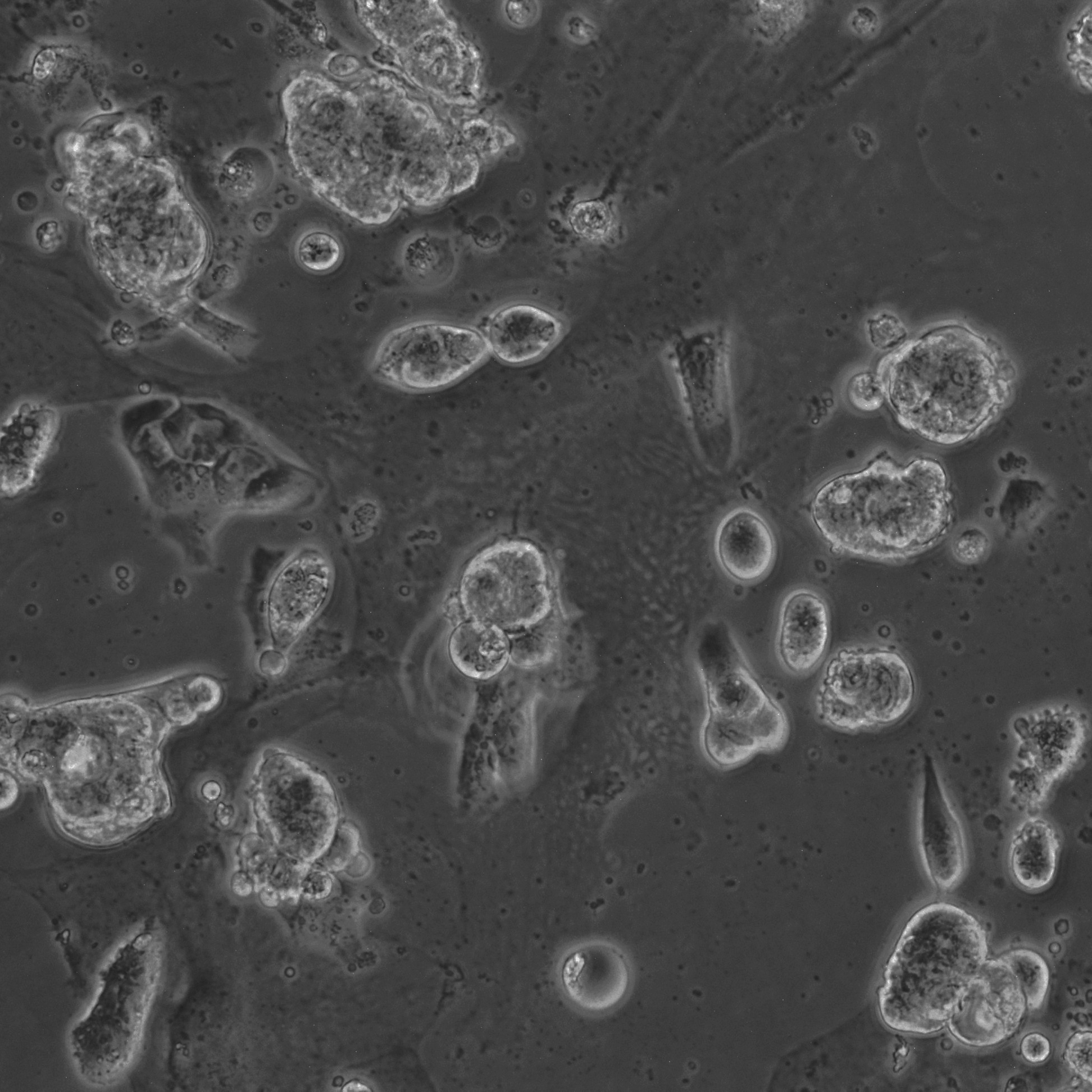

Supplement: Supplementary file 6 — Source data Fig. 4 [file 44319_2025_595_MOESM6_ESM.zip › Figure 4/4D/Control gRNA_Not Induced_D14_20x.jpg]

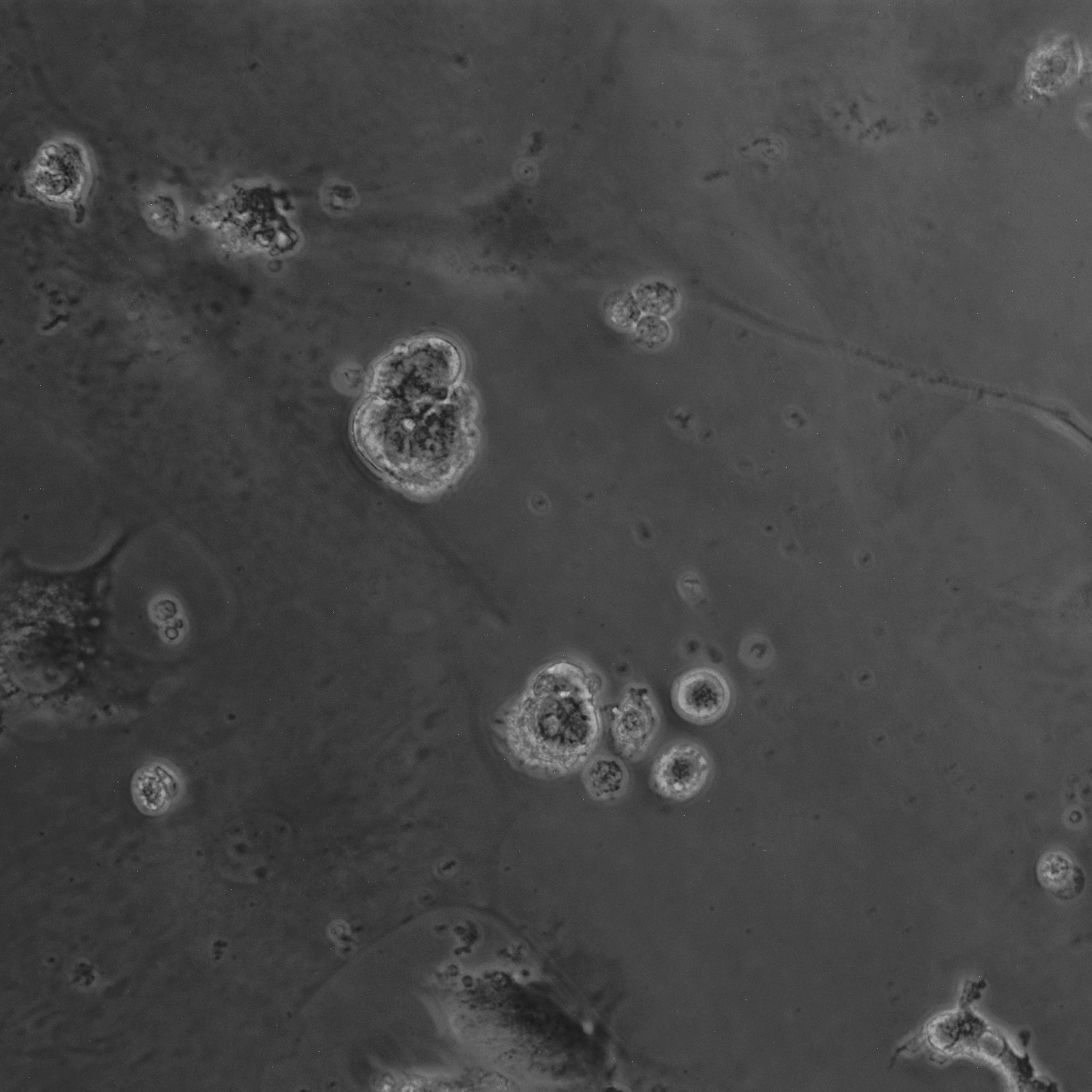

Supplement: Supplementary file 6 — Source data Fig. 4 [file 44319_2025_595_MOESM6_ESM.zip › Figure 4/4D/gRNA_KLF7_1_Induced_D14_20x.jpg]

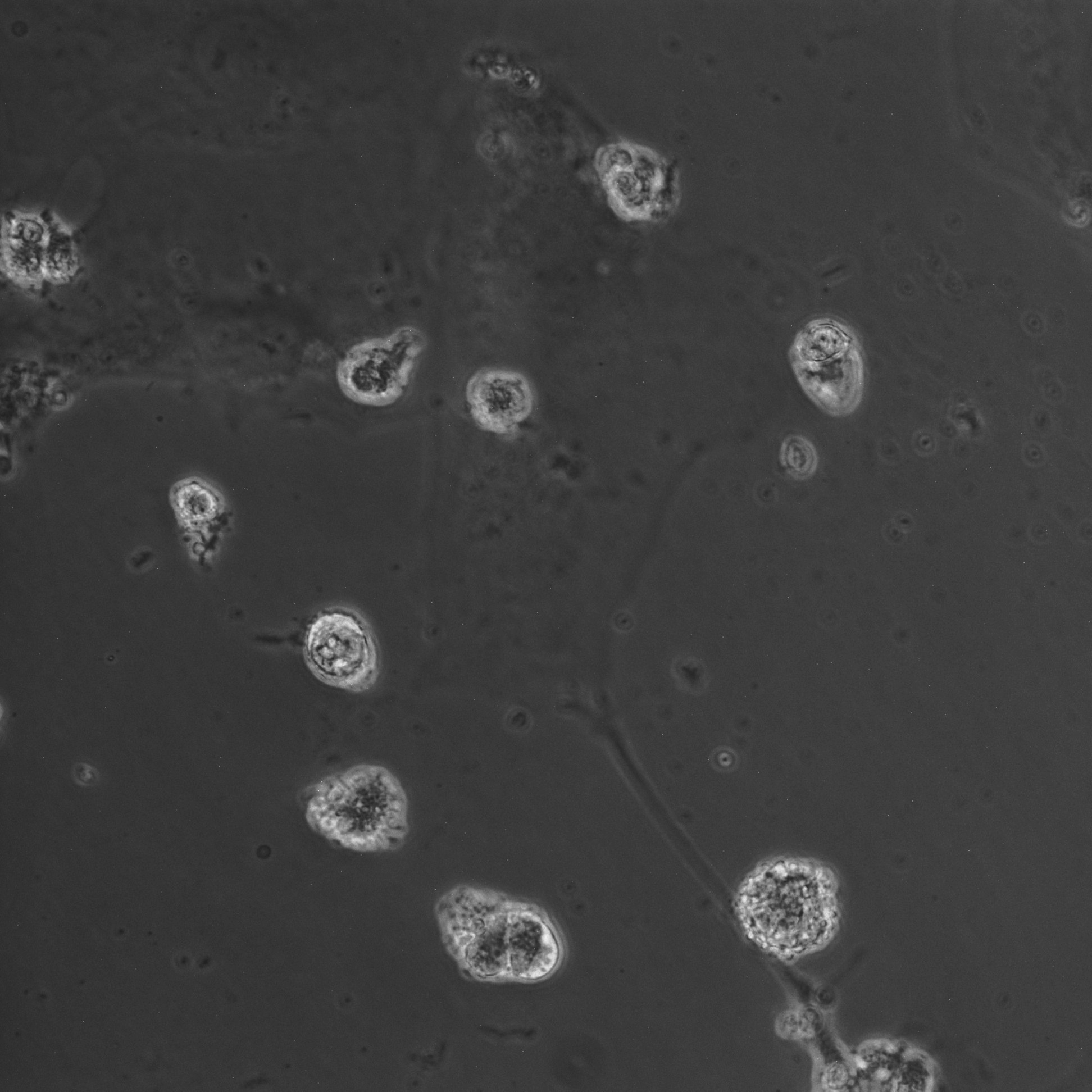

Supplement: Supplementary file 6 — Source data Fig. 4 [file 44319_2025_595_MOESM6_ESM.zip › Figure 4/4D/gRNA_KLF7_2_Induced_D14_20x.jpg]

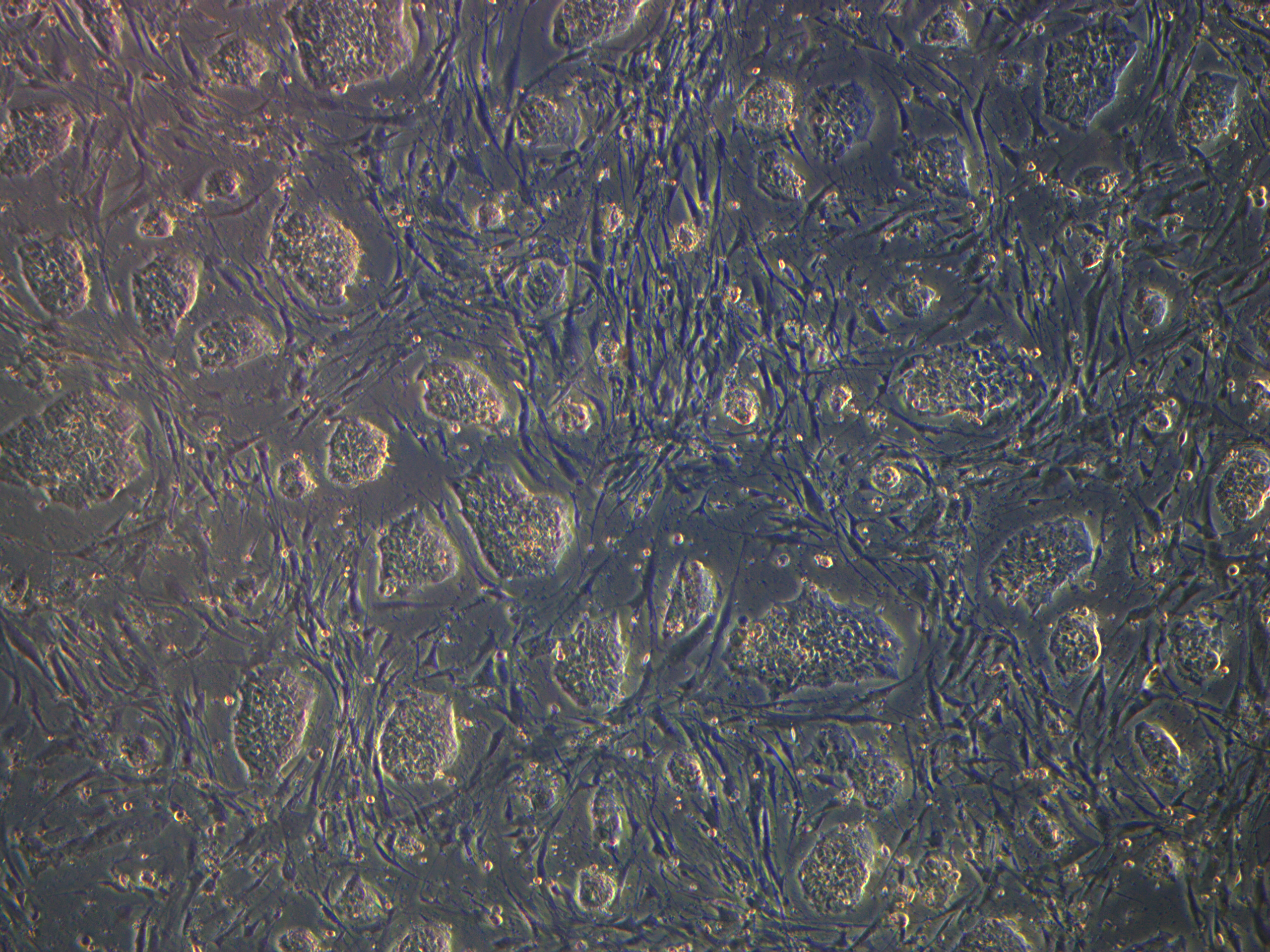

Supplement: Supplementary file 7 — Source data Fig. 5 [file 44319_2025_595_MOESM7_ESM.zip › Figure 5/5B/EMPTY-iPSC_D0.tiff]

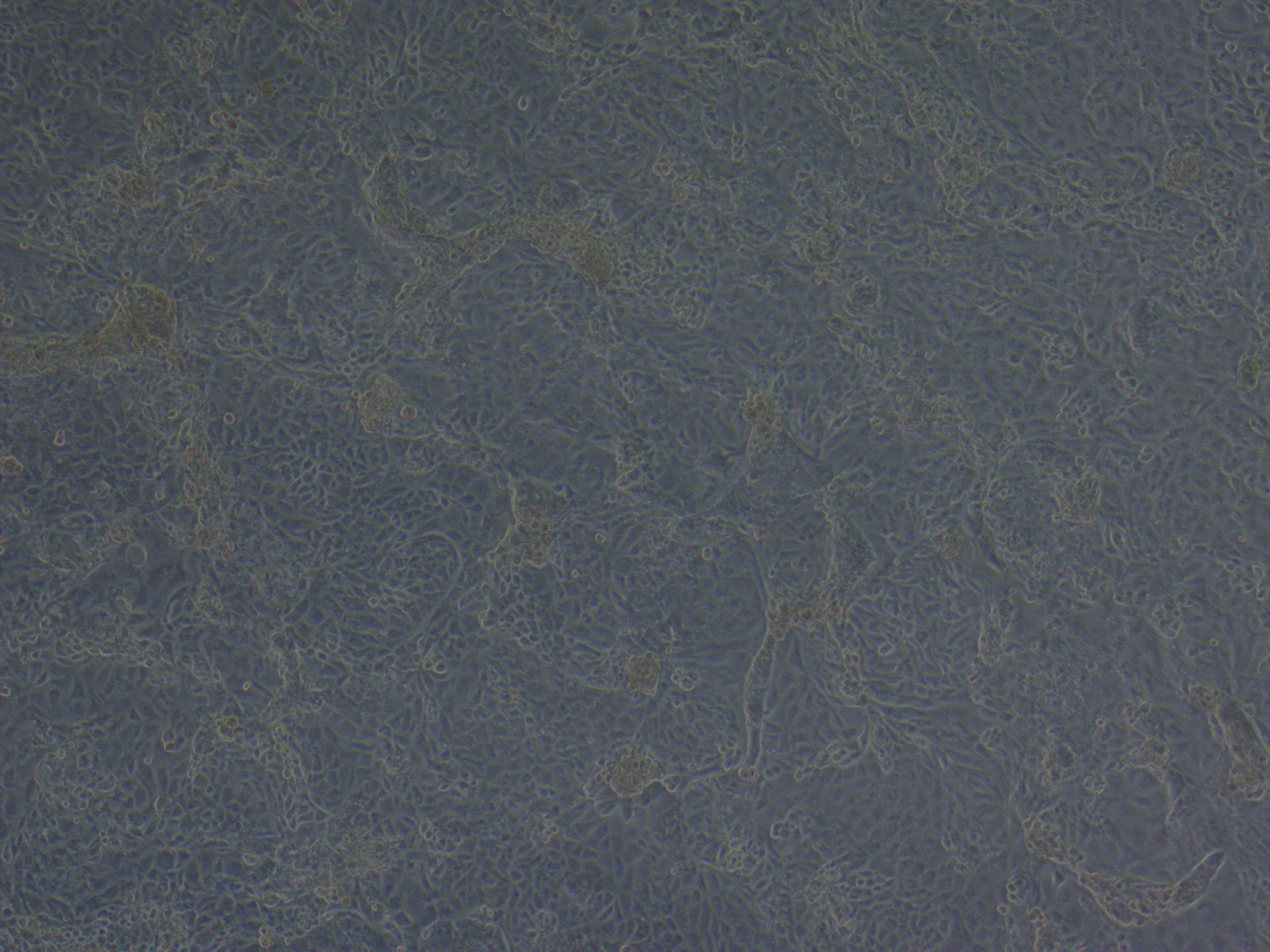

Supplement: Supplementary file 7 — Source data Fig. 5 [file 44319_2025_595_MOESM7_ESM.zip › Figure 5/5B/EMPTY-iPSC_D12_TSC.tiff]

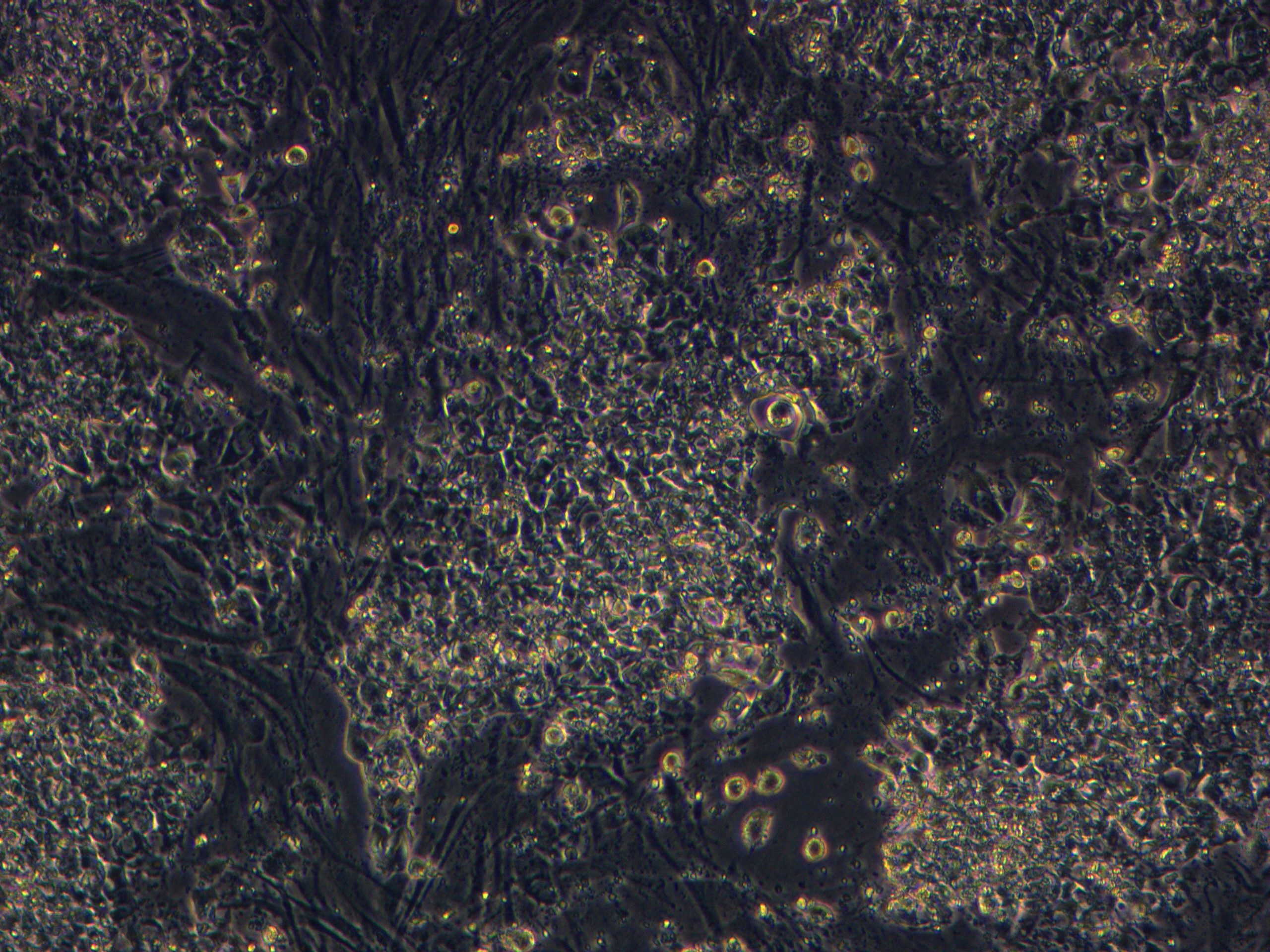

Supplement: Supplementary file 7 — Source data Fig. 5 [file 44319_2025_595_MOESM7_ESM.zip › Figure 5/5B/EMPTY-iPSC_D3.tiff]

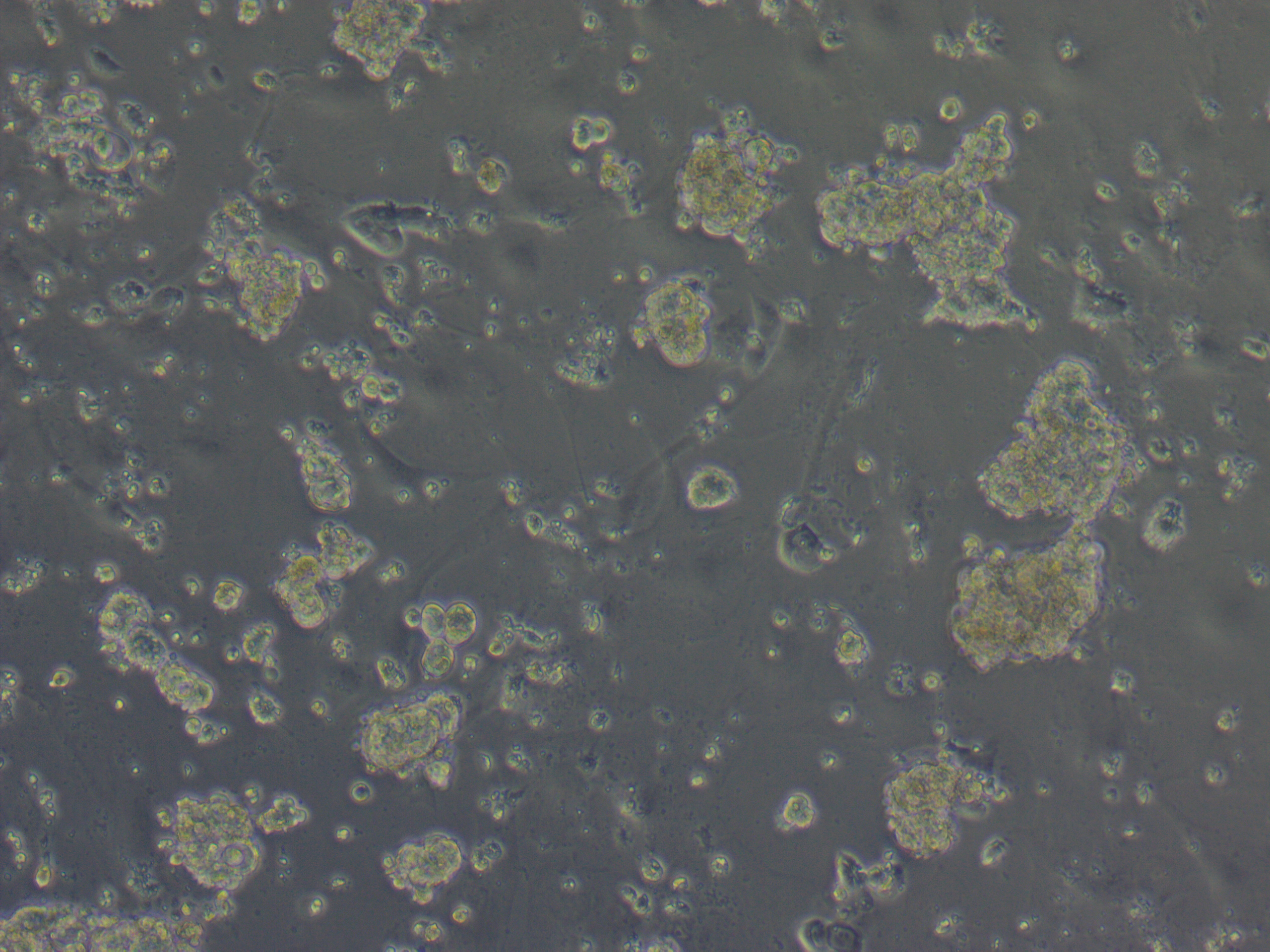

Supplement: Supplementary file 7 — Source data Fig. 5 [file 44319_2025_595_MOESM7_ESM.zip › Figure 5/5B/EMPTY-iPSC_D7_PXGL.tiff]

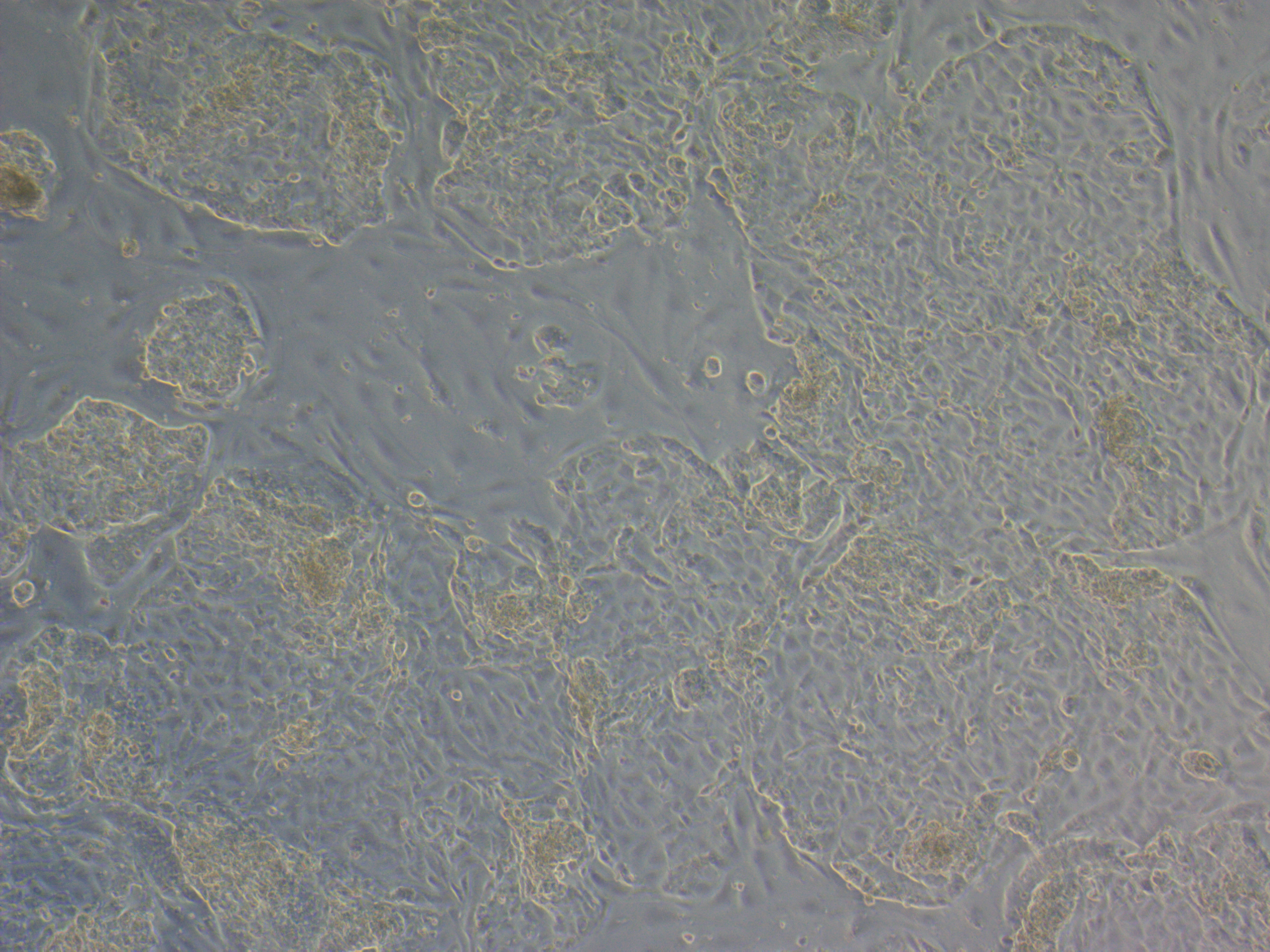

Supplement: Supplementary file 7 — Source data Fig. 5 [file 44319_2025_595_MOESM7_ESM.zip › Figure 5/5B/EMPTY-iPSC_D7_TSC.tiff]

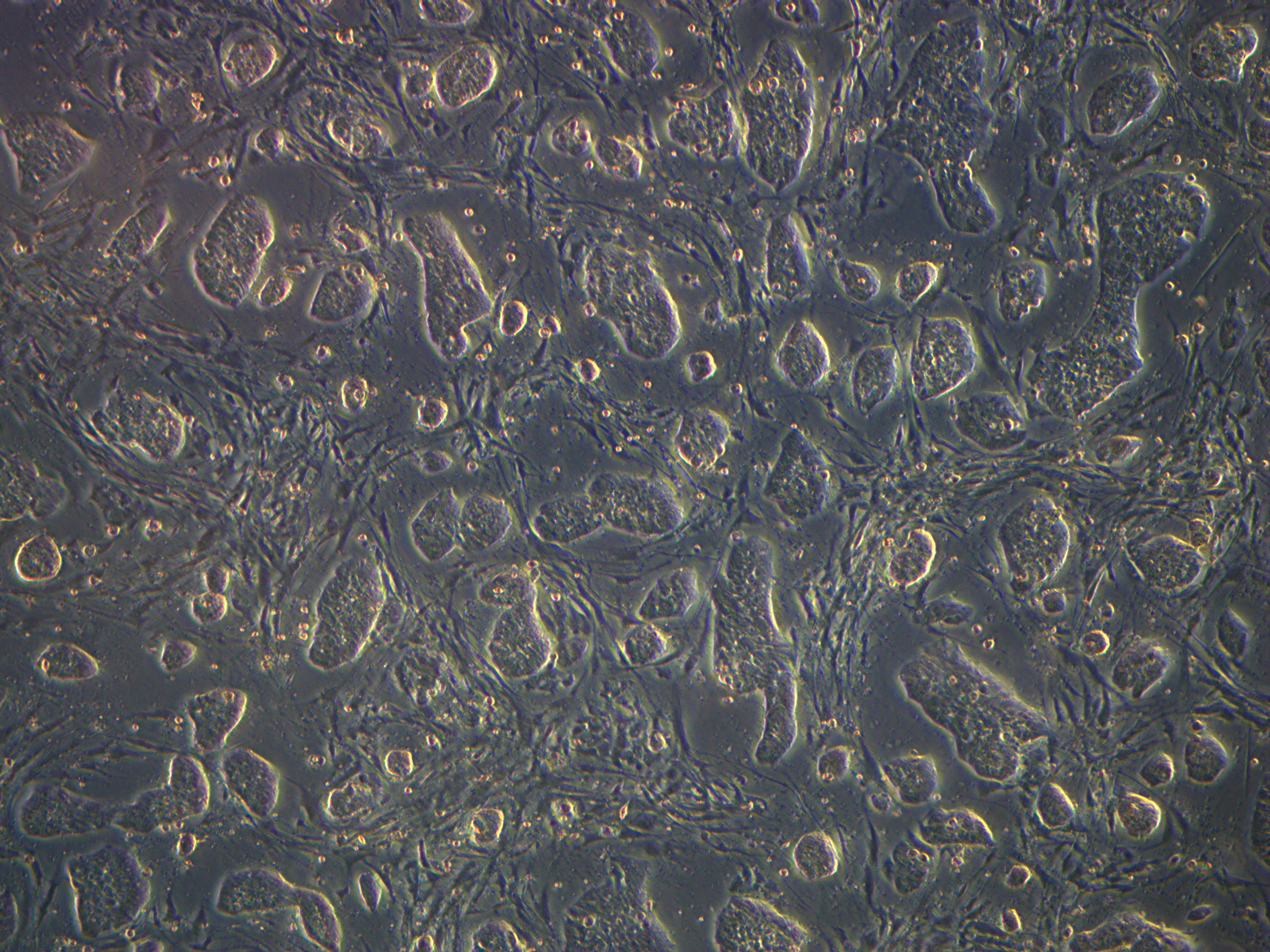

Supplement: Supplementary file 7 — Source data Fig. 5 [file 44319_2025_595_MOESM7_ESM.zip › Figure 5/5B/KLF7-iPSC_D0.tiff]

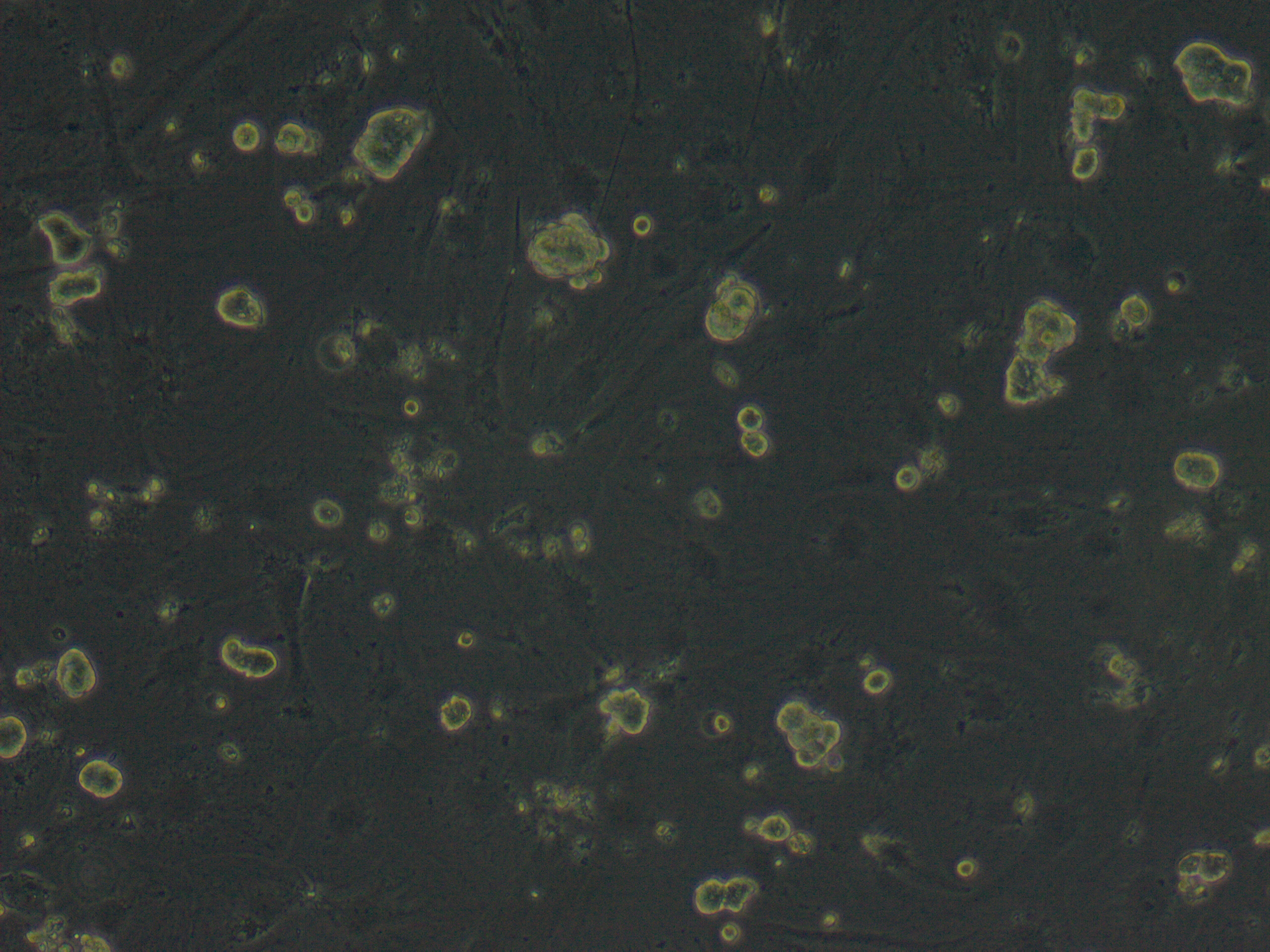

Supplement: Supplementary file 7 — Source data Fig. 5 [file 44319_2025_595_MOESM7_ESM.zip › Figure 5/5B/KLF7-iPSC_D12_PXGL.tiff]

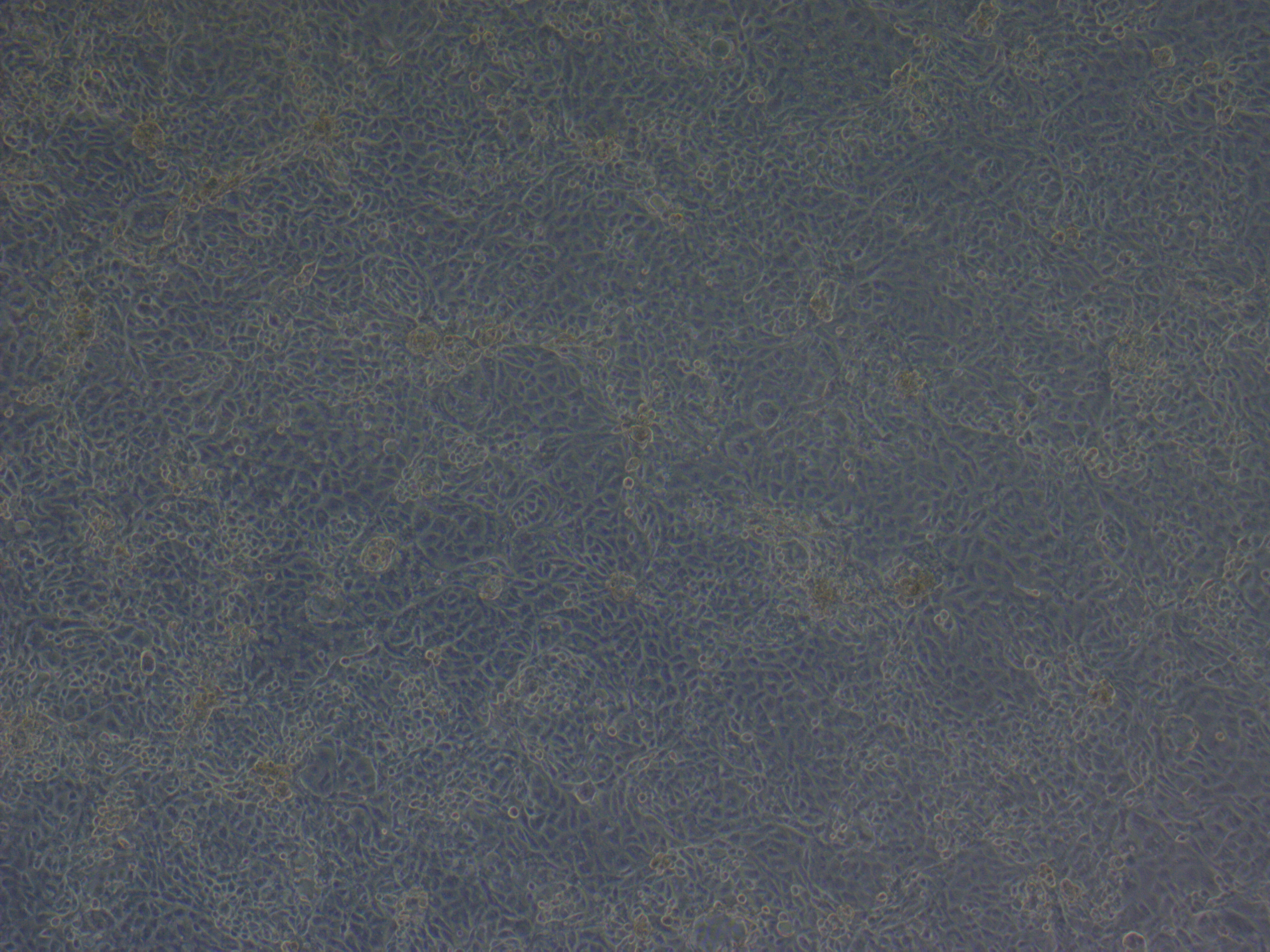

Supplement: Supplementary file 7 — Source data Fig. 5 [file 44319_2025_595_MOESM7_ESM.zip › Figure 5/5B/KLF7-iPSC_D12_TSC.tiff]

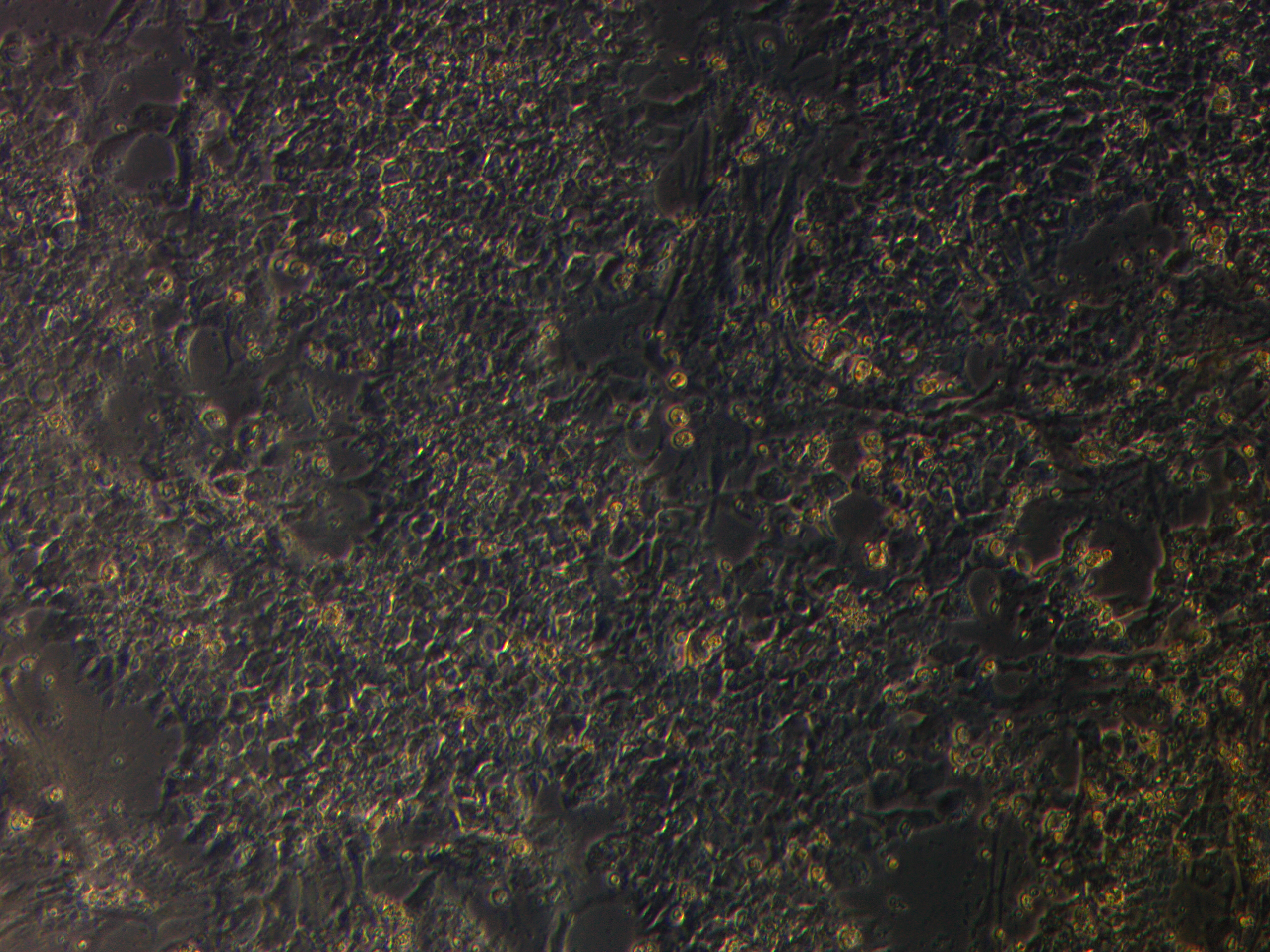

Supplement: Supplementary file 7 — Source data Fig. 5 [file 44319_2025_595_MOESM7_ESM.zip › Figure 5/5B/KLF7-iPSC_D3.tiff]

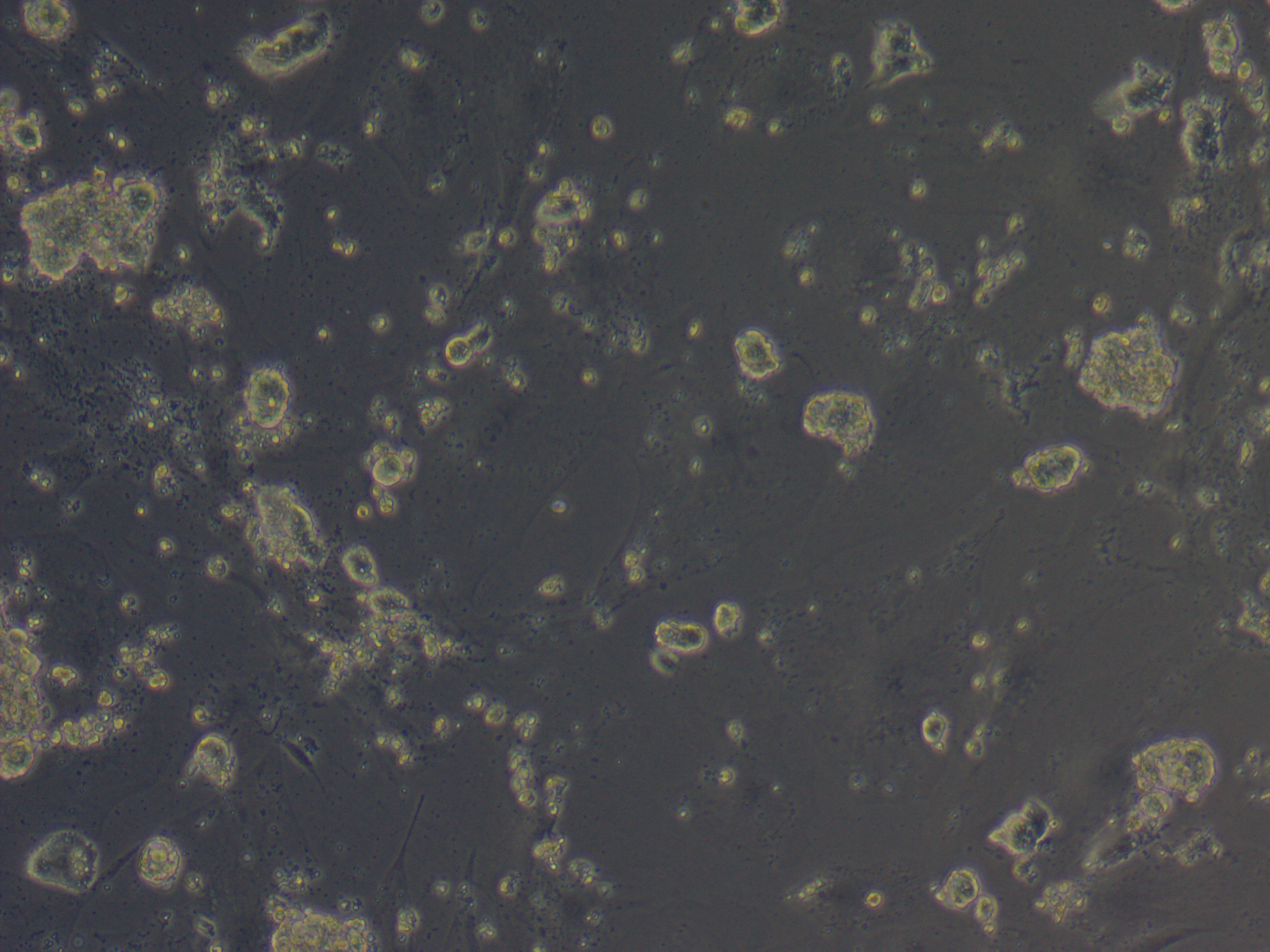

Supplement: Supplementary file 7 — Source data Fig. 5 [file 44319_2025_595_MOESM7_ESM.zip › Figure 5/5B/KLF7-iPSC_D7_PXGL.tiff]

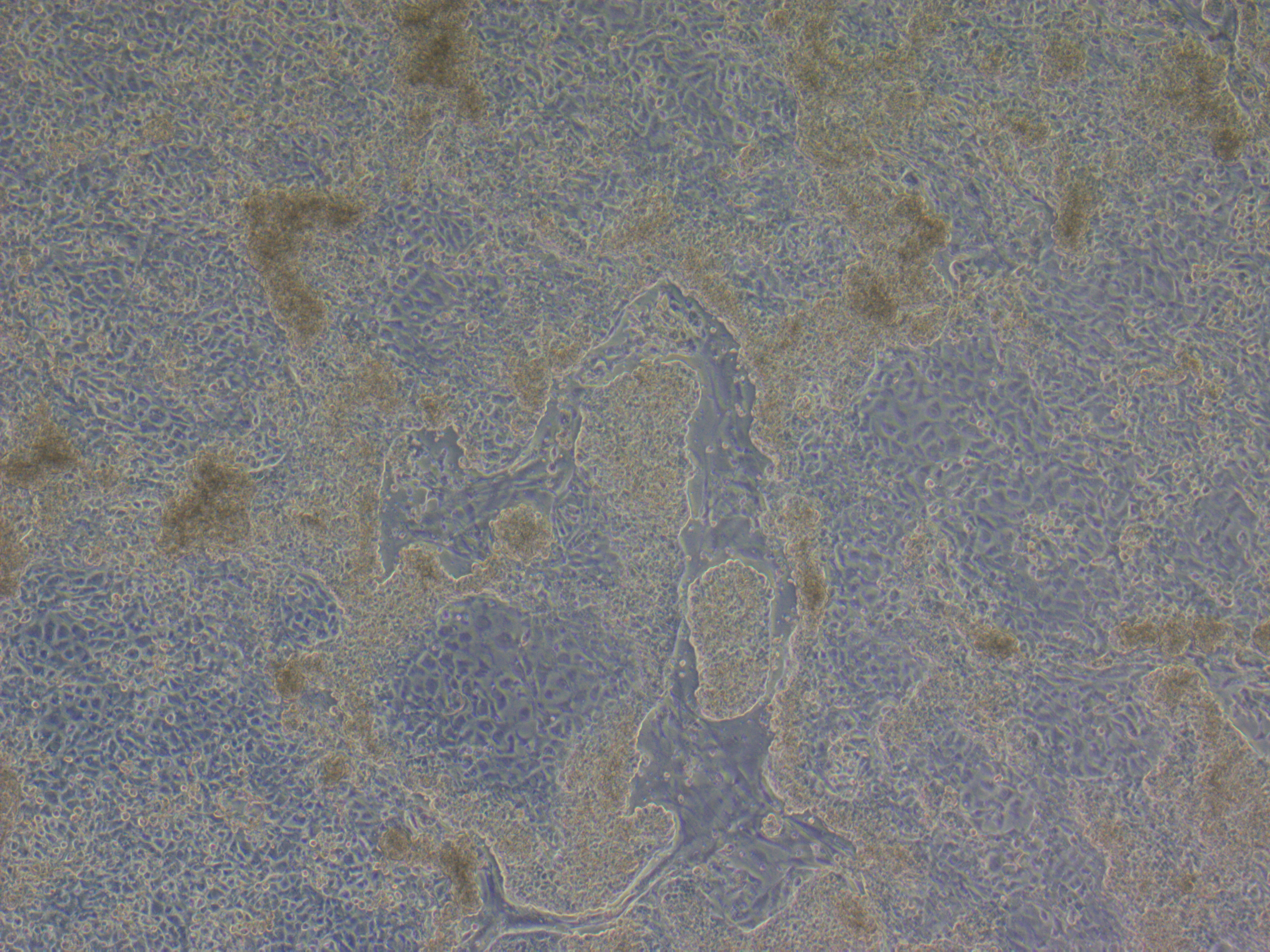

Supplement: Supplementary file 7 — Source data Fig. 5 [file 44319_2025_595_MOESM7_ESM.zip › Figure 5/5B/KLF7-iPSC_D7_TSC.tiff]

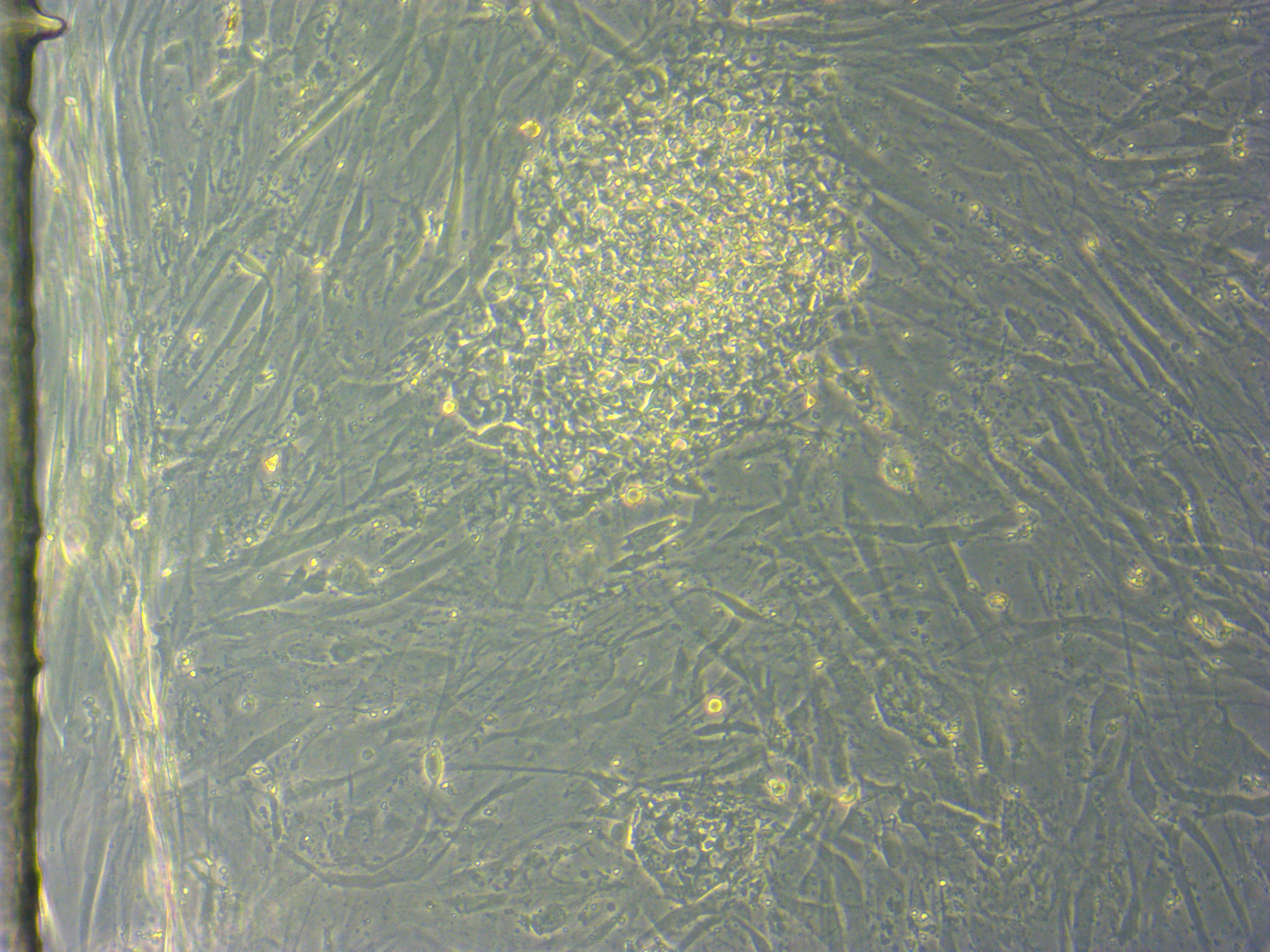

Supplement: Supplementary file 8 — Figure EV1 Source Data [file 44319_2025_595_MOESM8_ESM.zip › Figure EV1/EV1B/OSK7M_10X_Morphology.tiff]

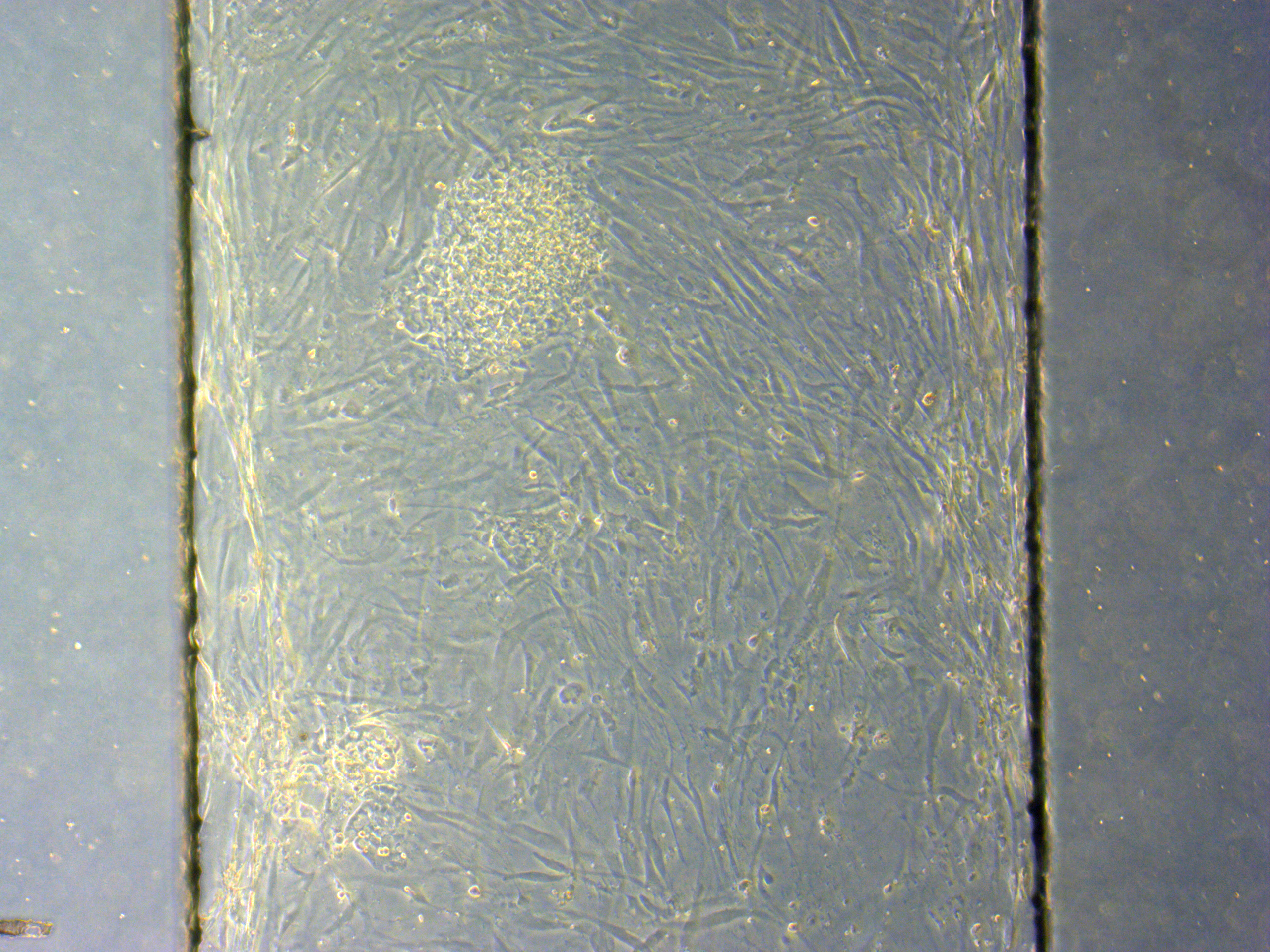

Supplement: Supplementary file 8 — Figure EV1 Source Data [file 44319_2025_595_MOESM8_ESM.zip › Figure EV1/EV1B/OSK7M_5X_Morphology.tiff]

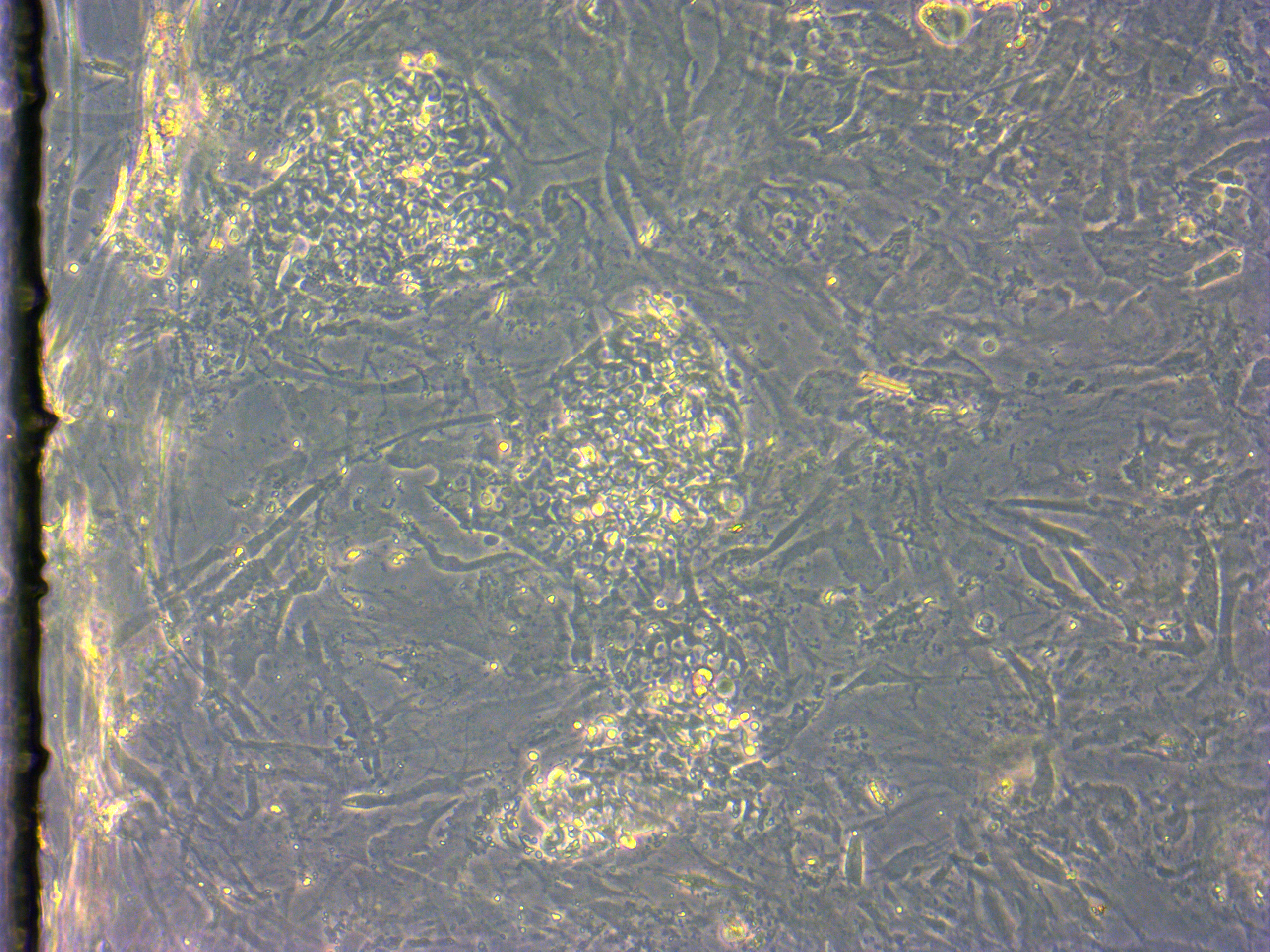

Supplement: Supplementary file 8 — Figure EV1 Source Data [file 44319_2025_595_MOESM8_ESM.zip › Figure EV1/EV1B/OSKM 10X_Morphology.tiff]

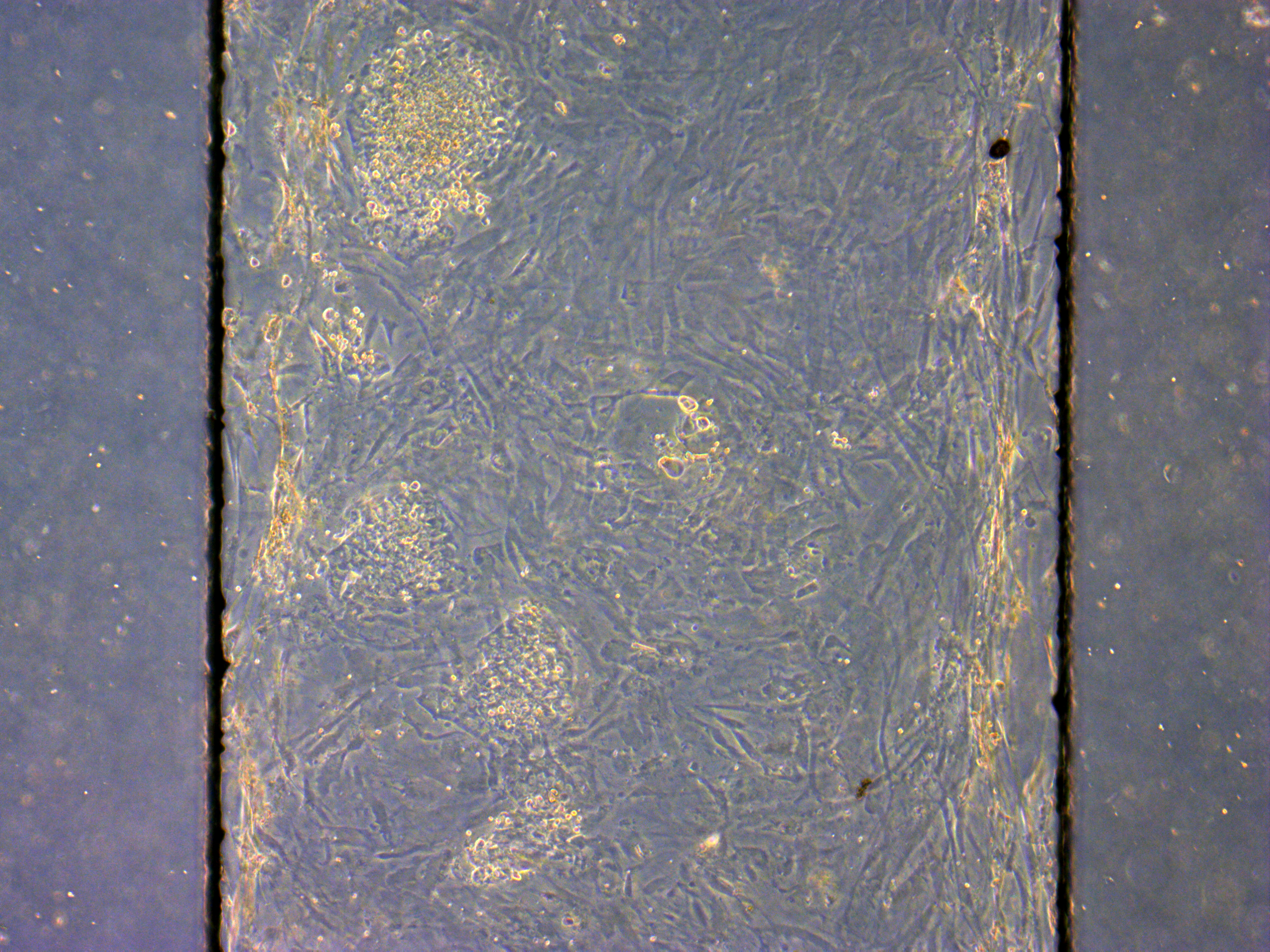

Supplement: Supplementary file 8 — Figure EV1 Source Data [file 44319_2025_595_MOESM8_ESM.zip › Figure EV1/EV1B/OSKM 5X_Morphology.tiff]

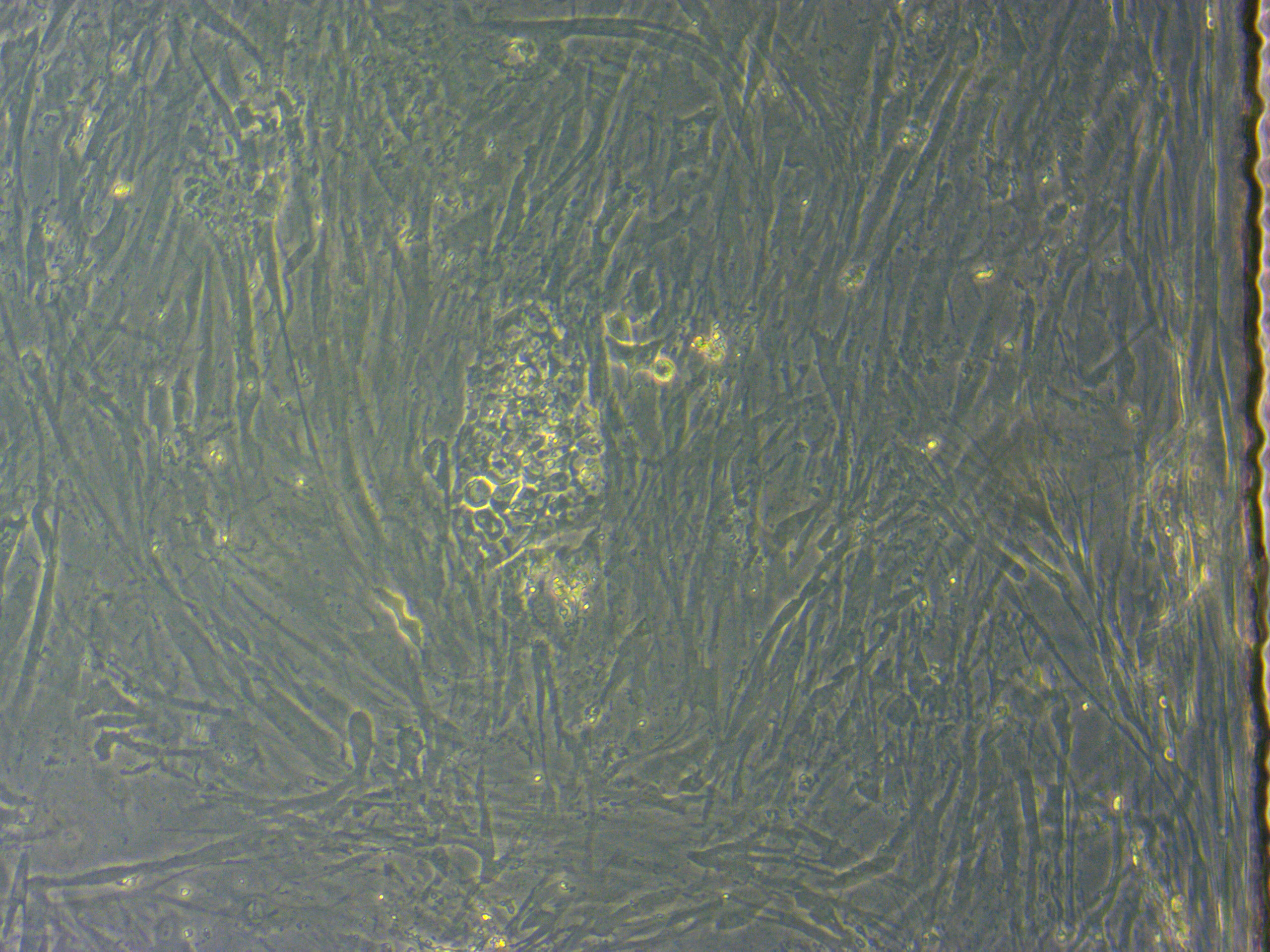

Supplement: Supplementary file 8 — Figure EV1 Source Data [file 44319_2025_595_MOESM8_ESM.zip › Figure EV1/EV1B/OSNL_10X_Morphology.tiff]

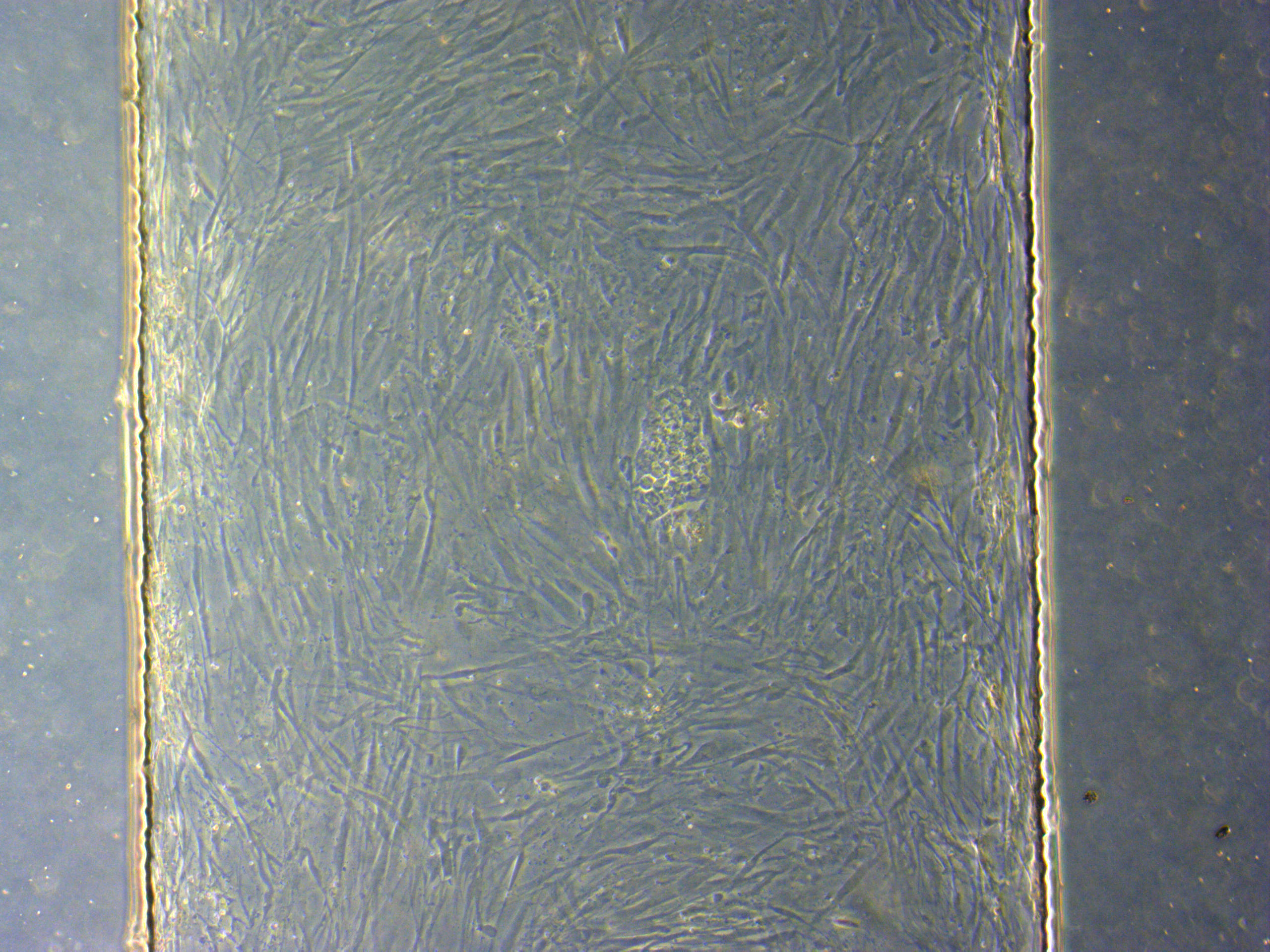

Supplement: Supplementary file 8 — Figure EV1 Source Data [file 44319_2025_595_MOESM8_ESM.zip › Figure EV1/EV1B/OSNL_5X_Morphology.tiff]

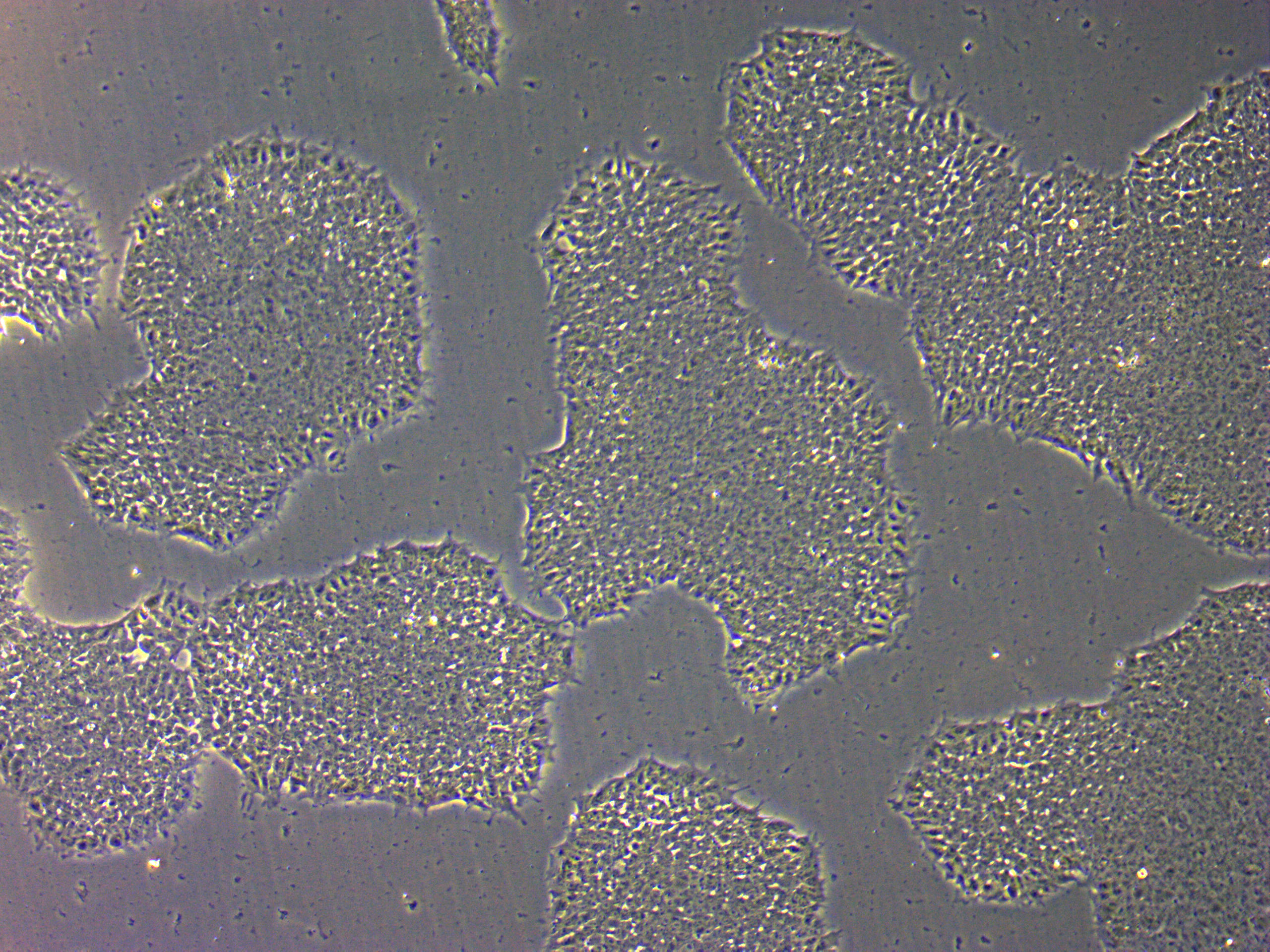

Supplement: Supplementary file 8 — Figure EV1 Source Data [file 44319_2025_595_MOESM8_ESM.zip › Figure EV1/EV1C/OSK7M P10_Morphology.tiff]

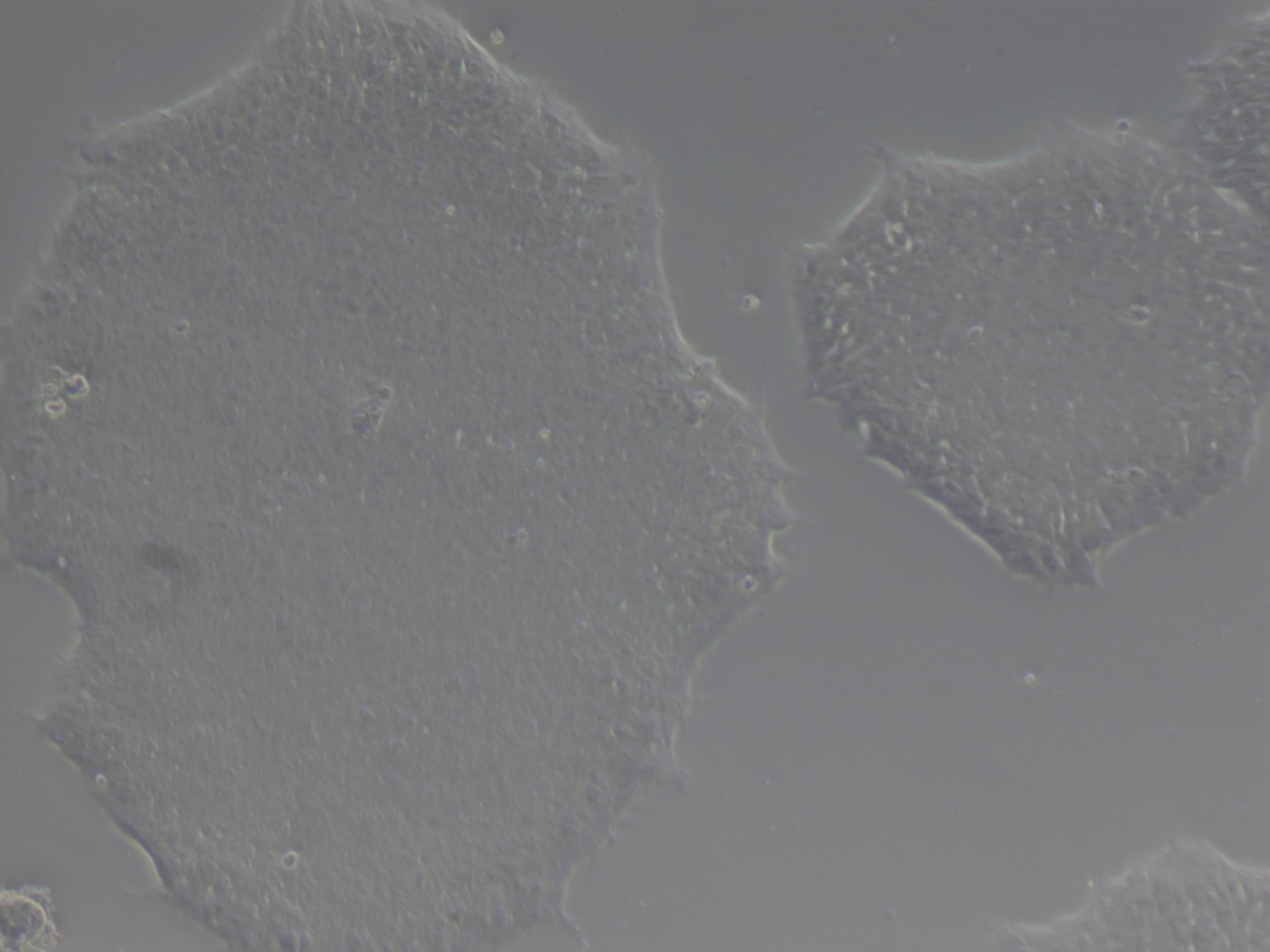

Supplement: Supplementary file 8 — Figure EV1 Source Data [file 44319_2025_595_MOESM8_ESM.zip › Figure EV1/EV1C/OSK7M P15_Morphology.tiff]

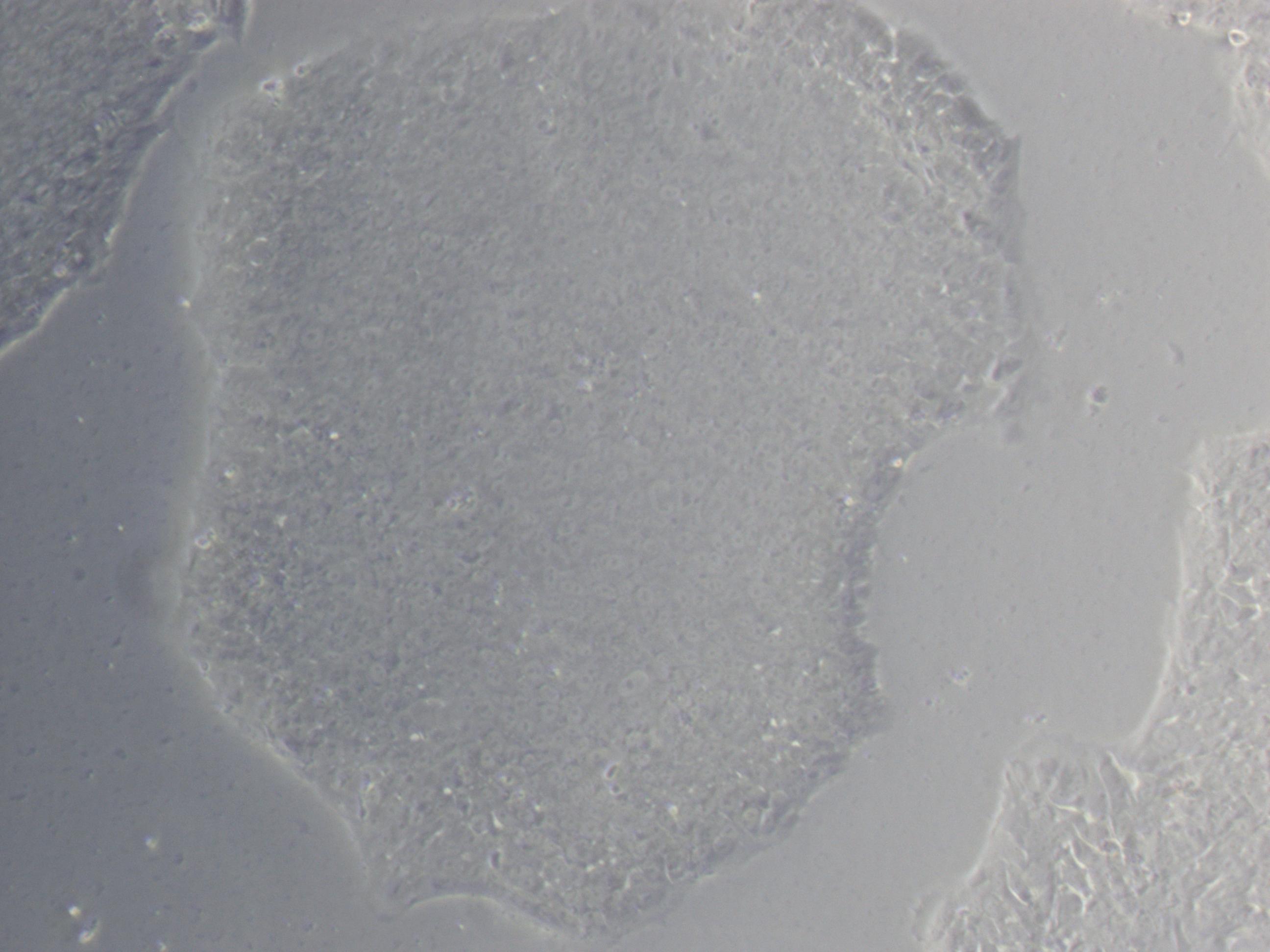

Supplement: Supplementary file 8 — Figure EV1 Source Data [file 44319_2025_595_MOESM8_ESM.zip › Figure EV1/EV1C/OSK7M P20_Morphology.tiff]

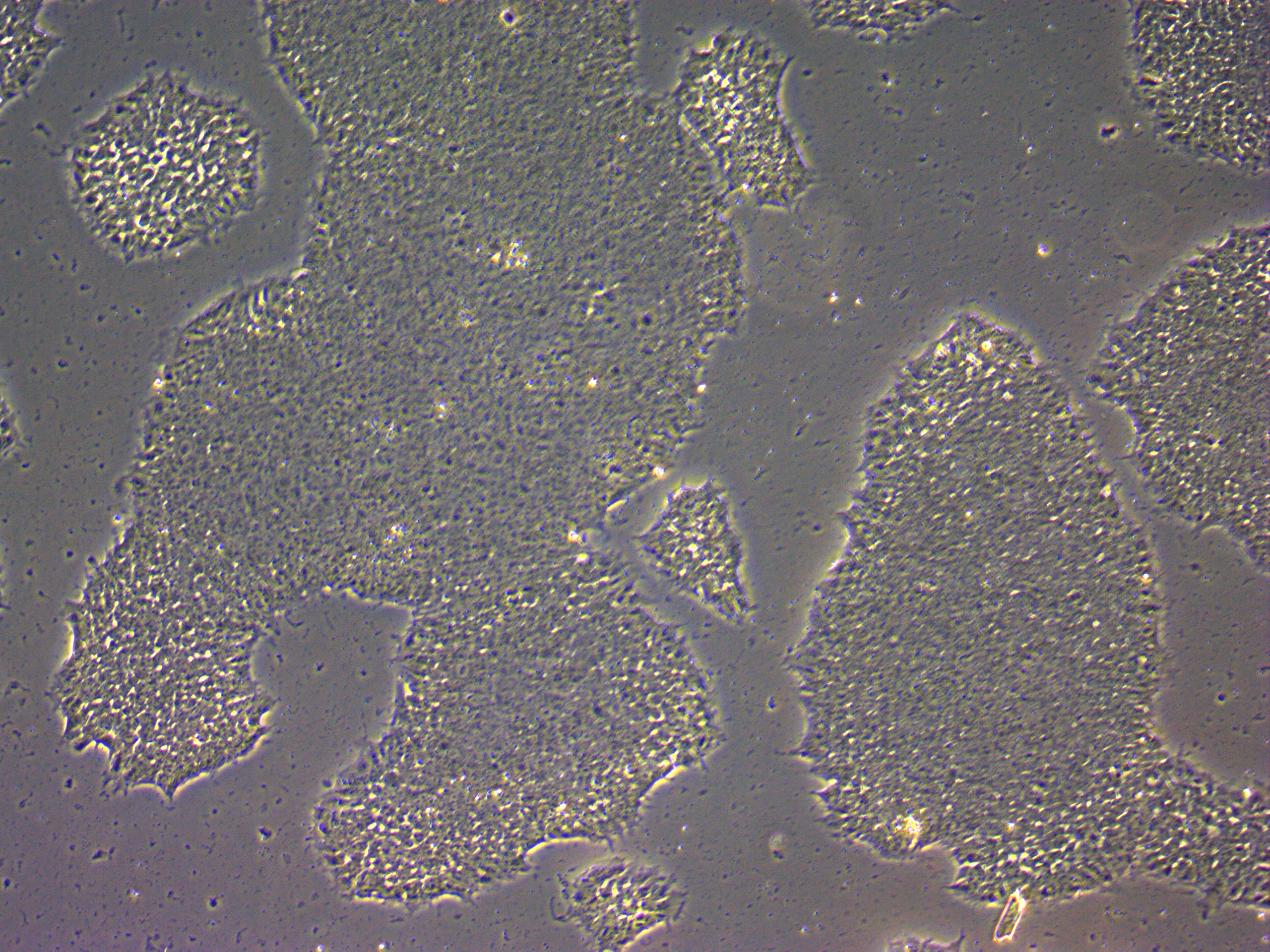

Supplement: Supplementary file 8 — Figure EV1 Source Data [file 44319_2025_595_MOESM8_ESM.zip › Figure EV1/EV1C/OSKM P10_Morphology.tiff]

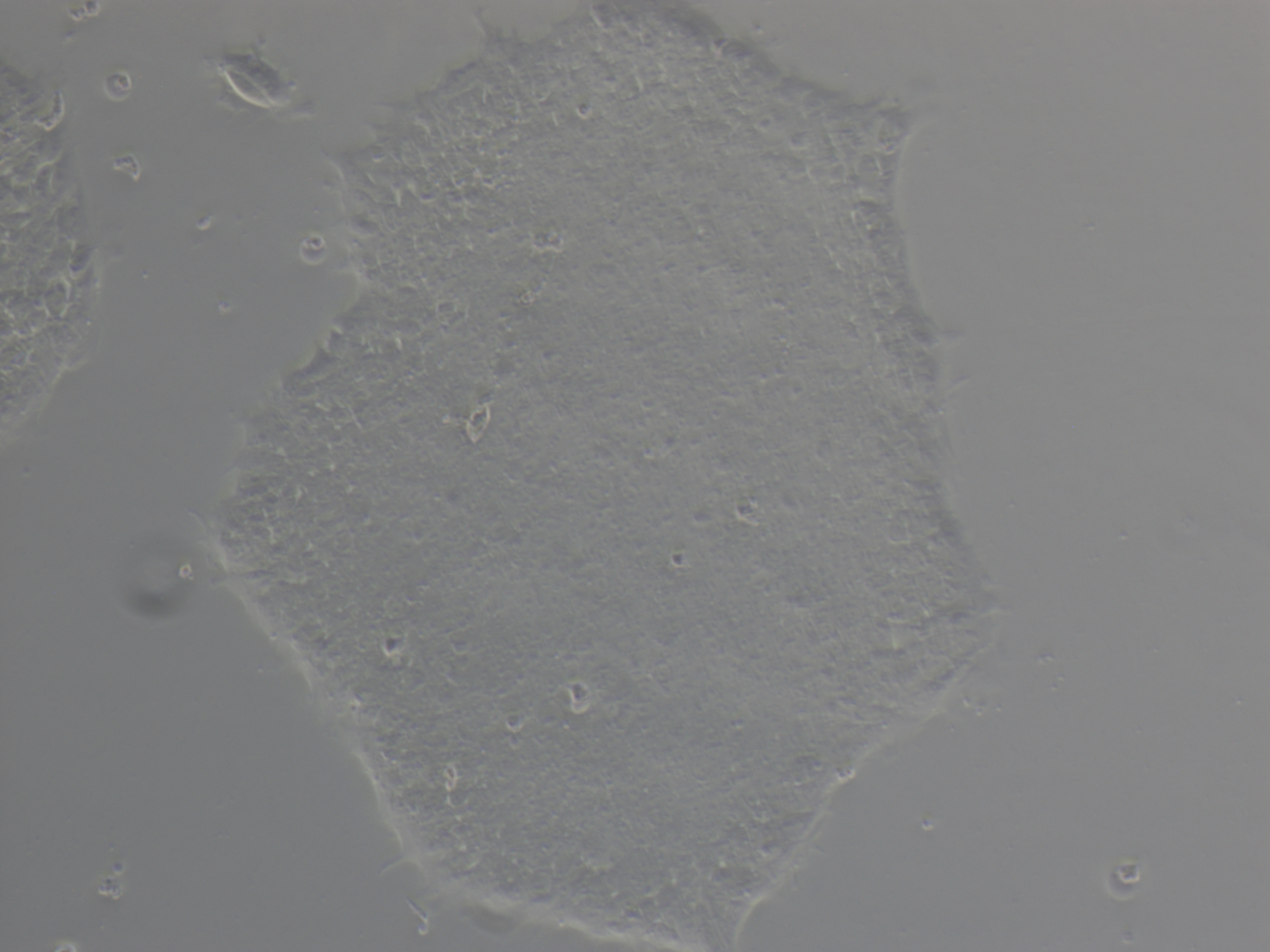

Supplement: Supplementary file 8 — Figure EV1 Source Data [file 44319_2025_595_MOESM8_ESM.zip › Figure EV1/EV1C/OSKM P15_Morphology.tiff]

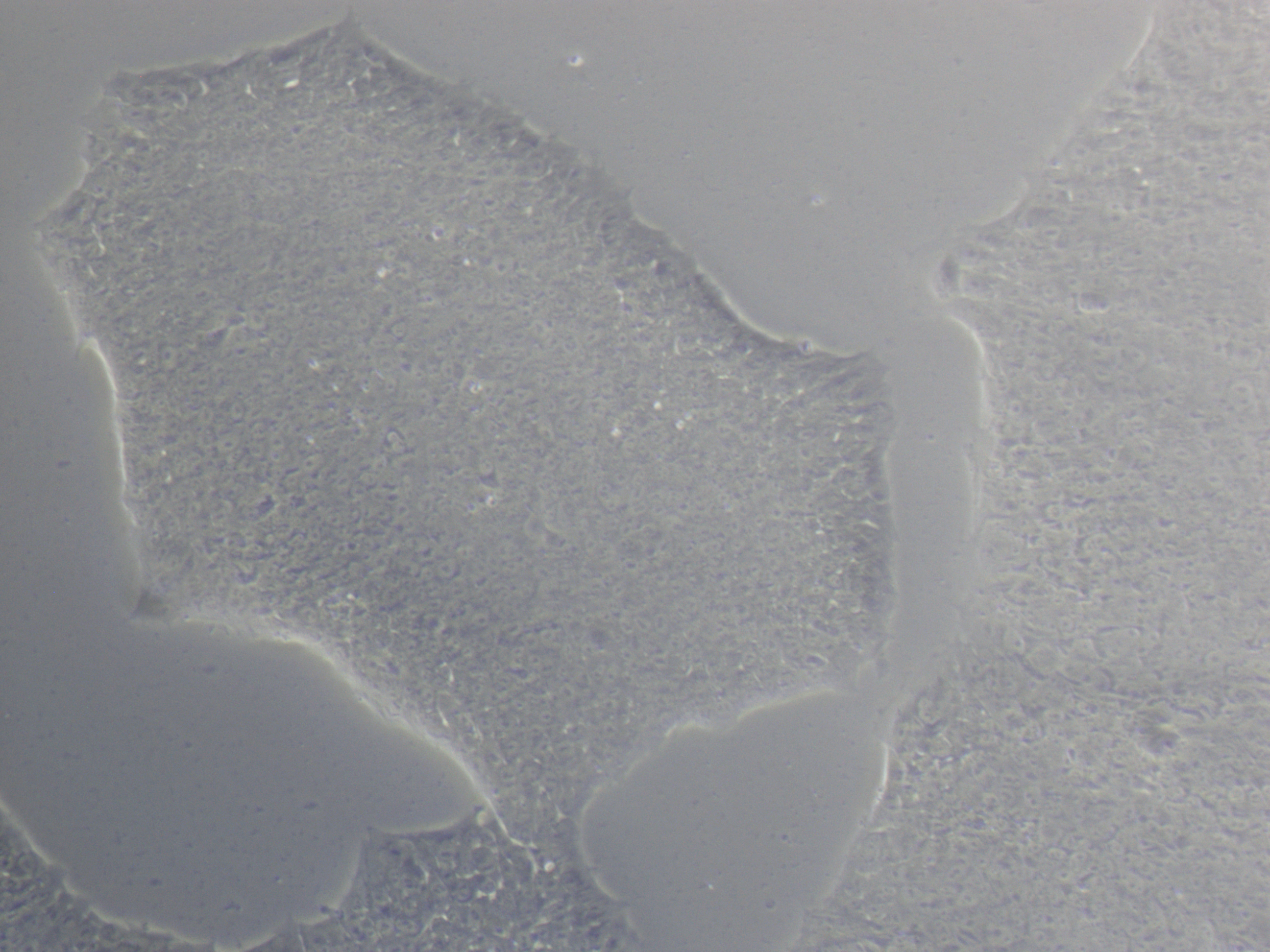

Supplement: Supplementary file 8 — Figure EV1 Source Data [file 44319_2025_595_MOESM8_ESM.zip › Figure EV1/EV1C/OSKM P20_Morphology.tiff]

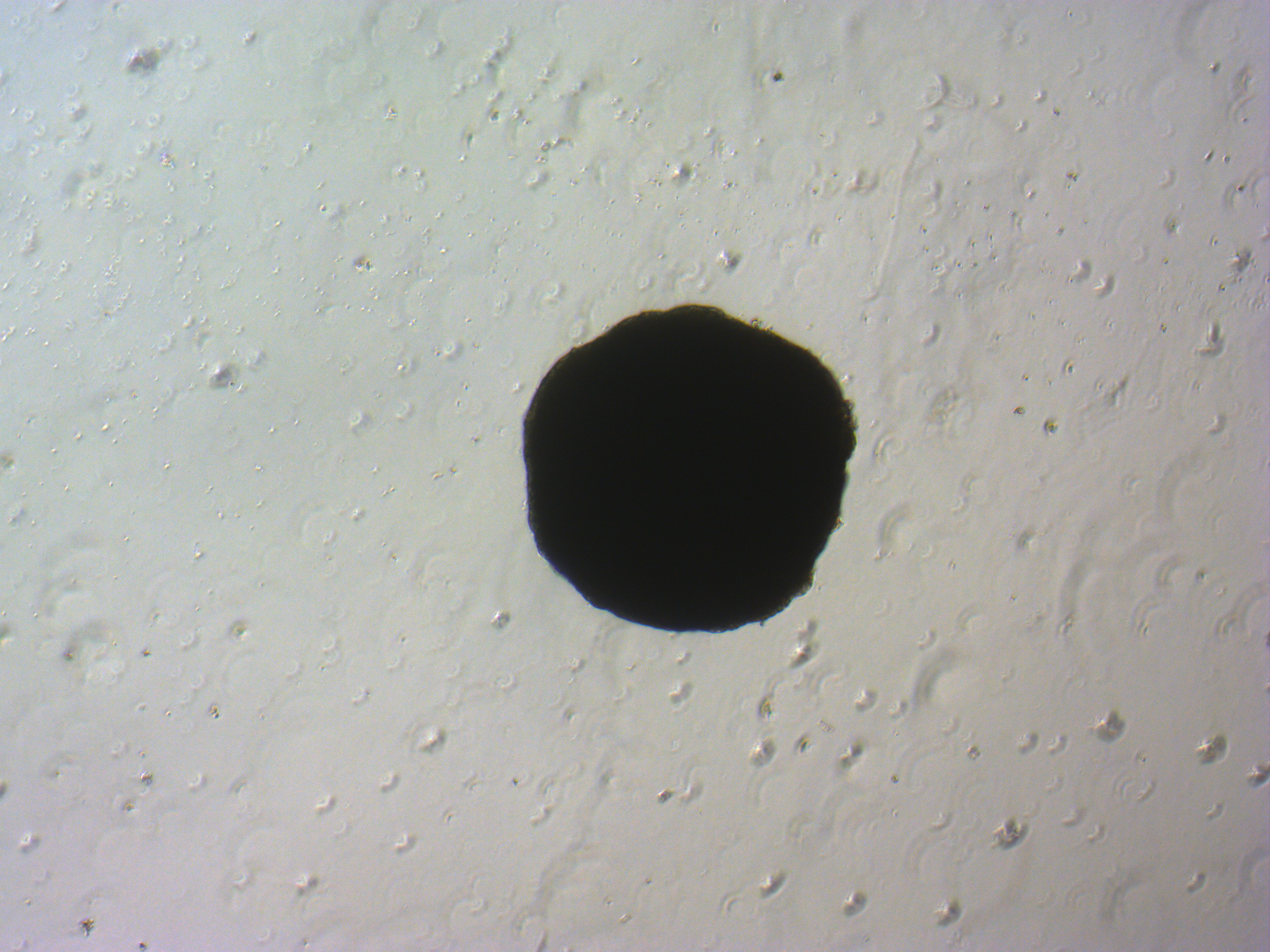

Supplement: Supplementary file 9 — Figure EV2 Source Data [file 44319_2025_595_MOESM9_ESM.zip › Figure EV2/EV2B/OSK7M Day 15 1.tiff]

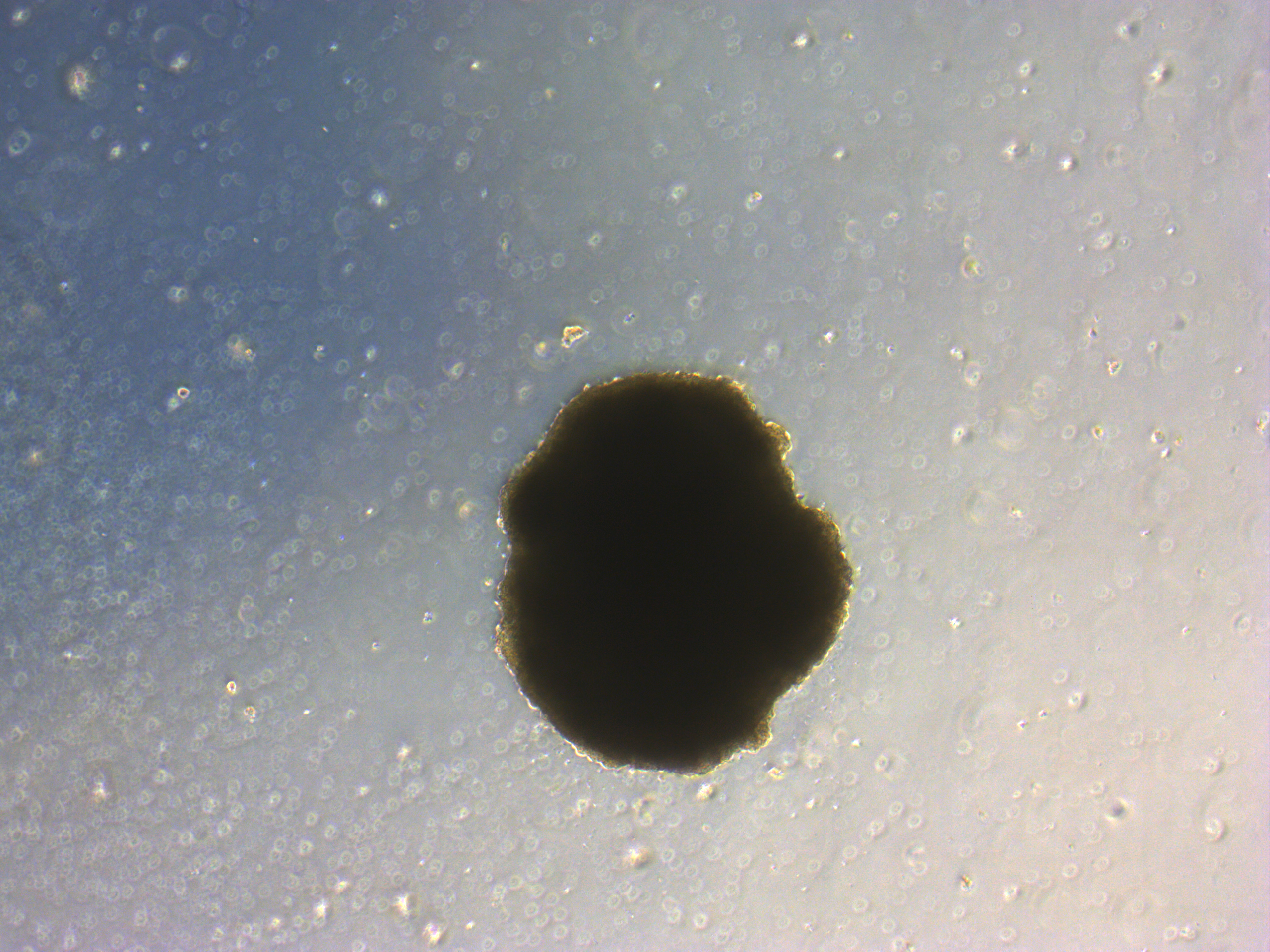

Supplement: Supplementary file 9 — Figure EV2 Source Data [file 44319_2025_595_MOESM9_ESM.zip › Figure EV2/EV2B/OSK7M Day 15 2.tiff]

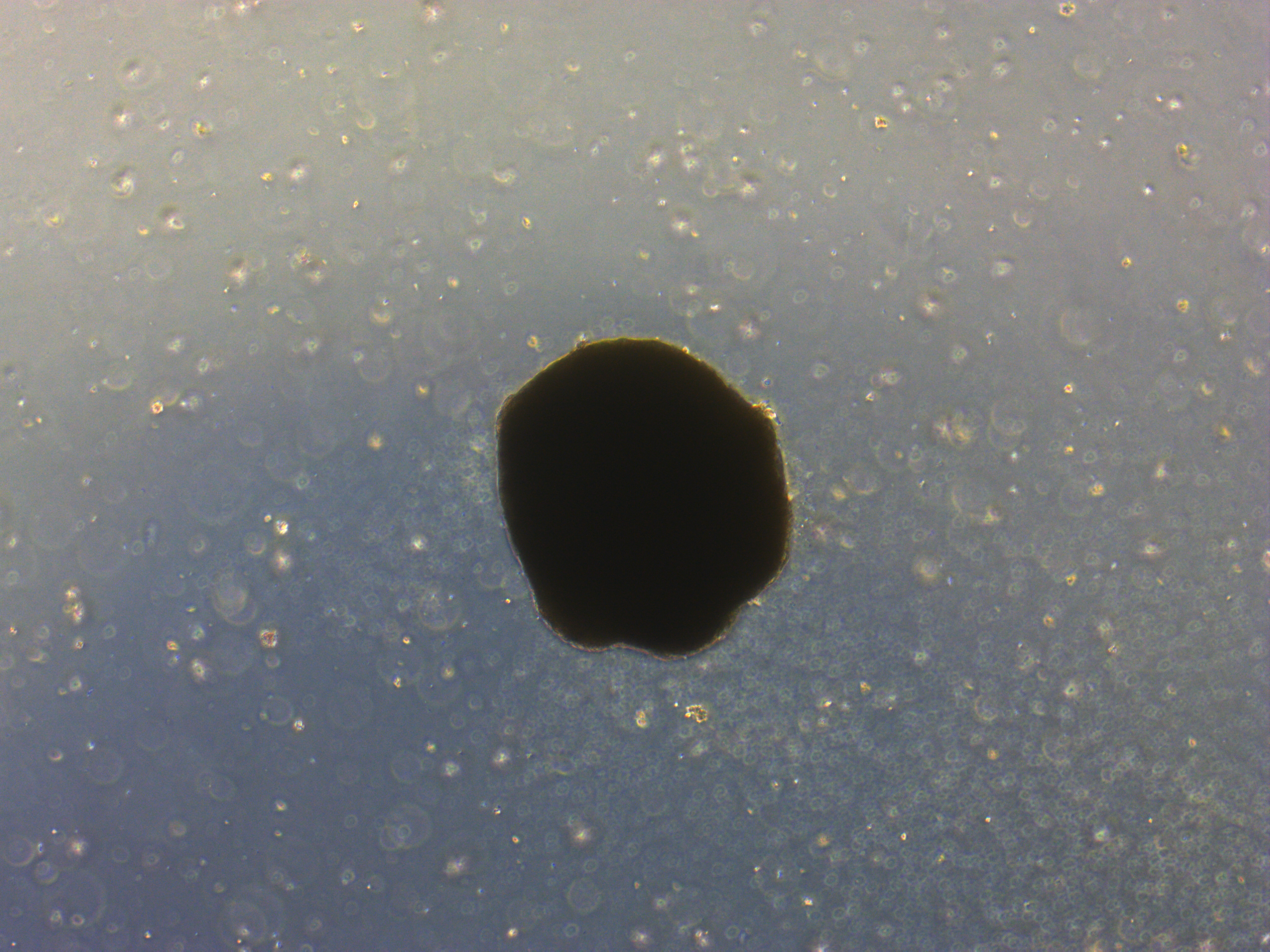

Supplement: Supplementary file 9 — Figure EV2 Source Data [file 44319_2025_595_MOESM9_ESM.zip › Figure EV2/EV2B/OSK7M Day 15 3.tiff]

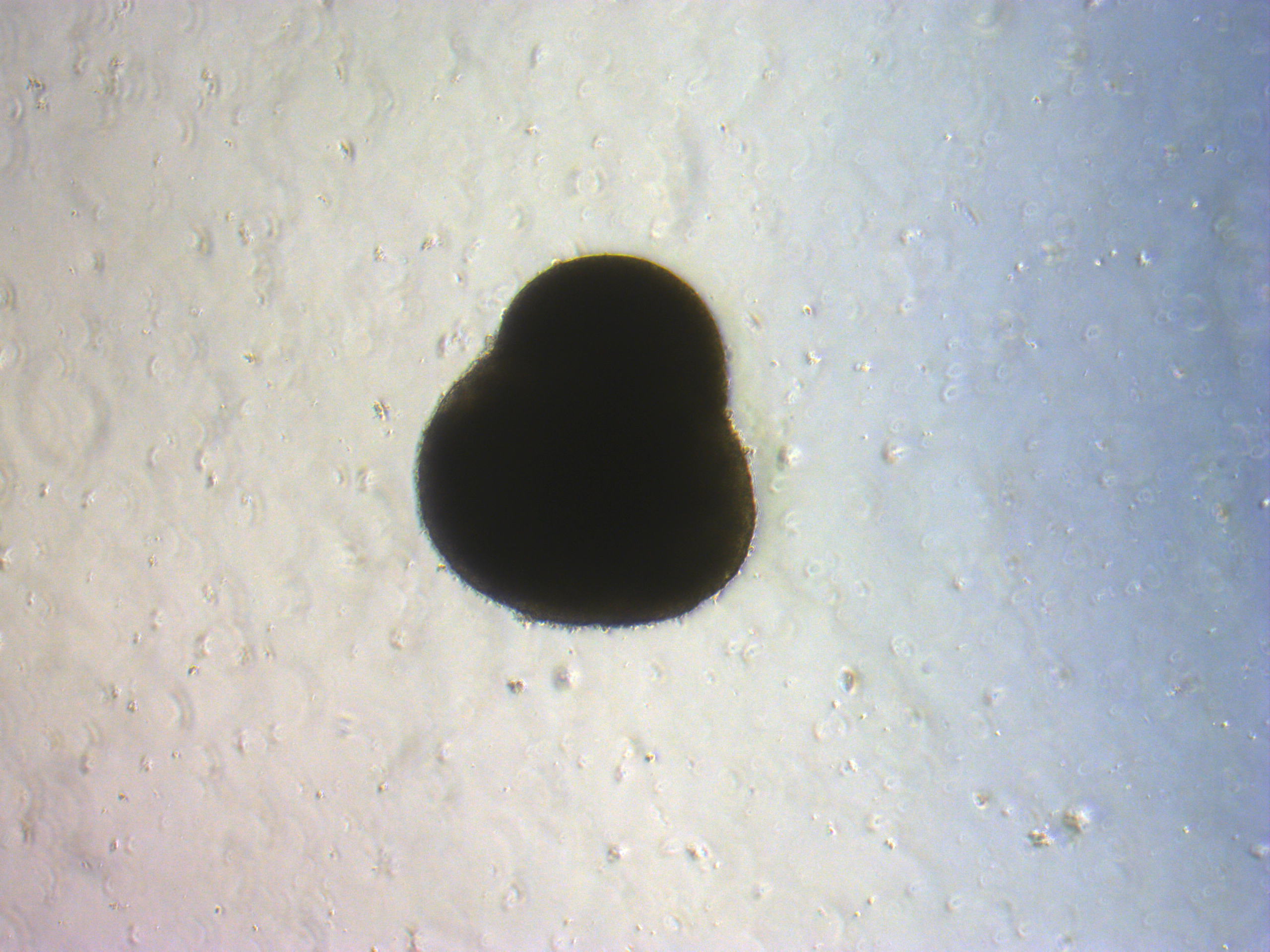

Supplement: Supplementary file 9 — Figure EV2 Source Data [file 44319_2025_595_MOESM9_ESM.zip › Figure EV2/EV2B/OSKM Day 15 1.tiff]

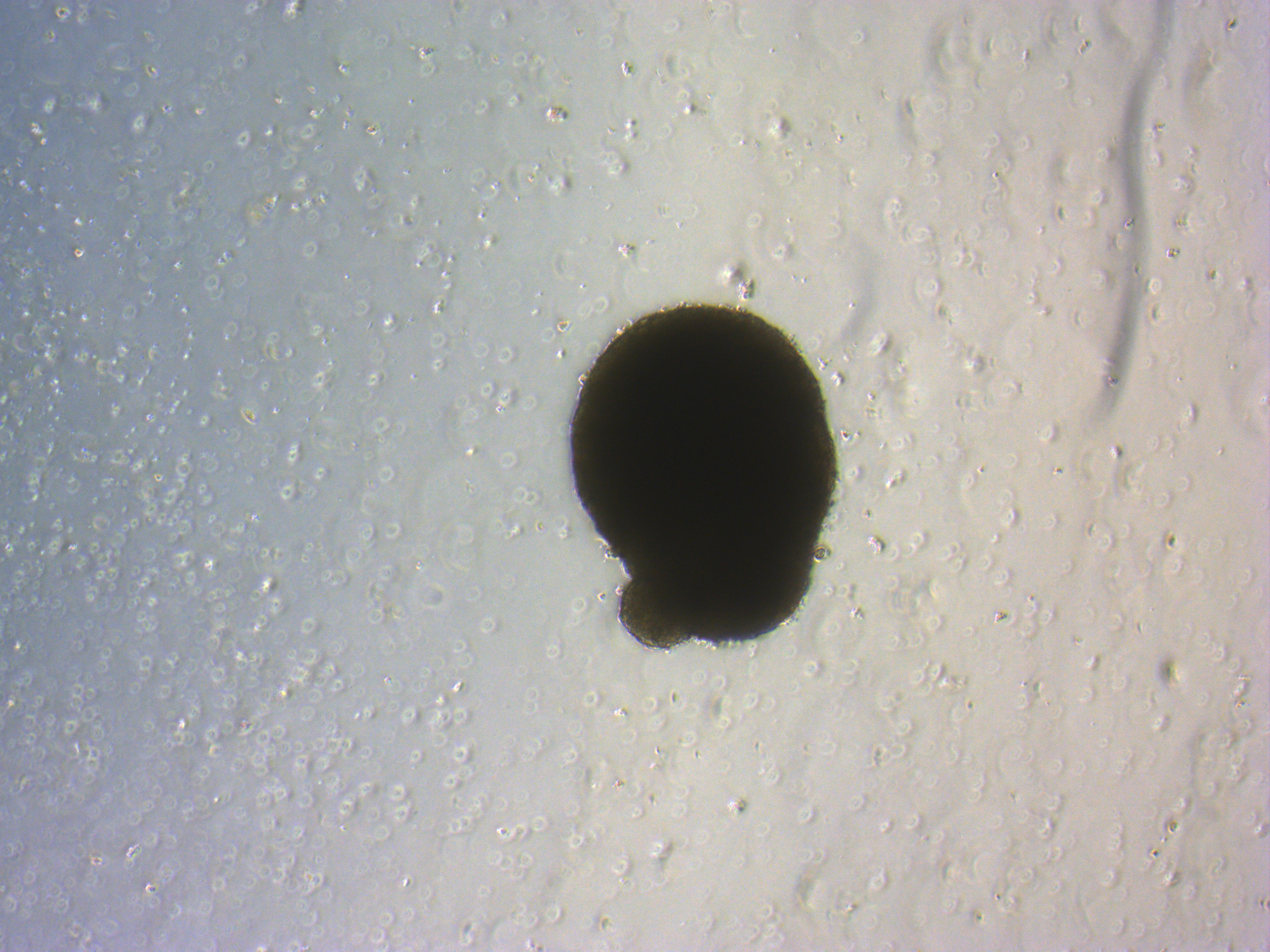

Supplement: Supplementary file 9 — Figure EV2 Source Data [file 44319_2025_595_MOESM9_ESM.zip › Figure EV2/EV2B/OSKM Day 15 2.tiff]

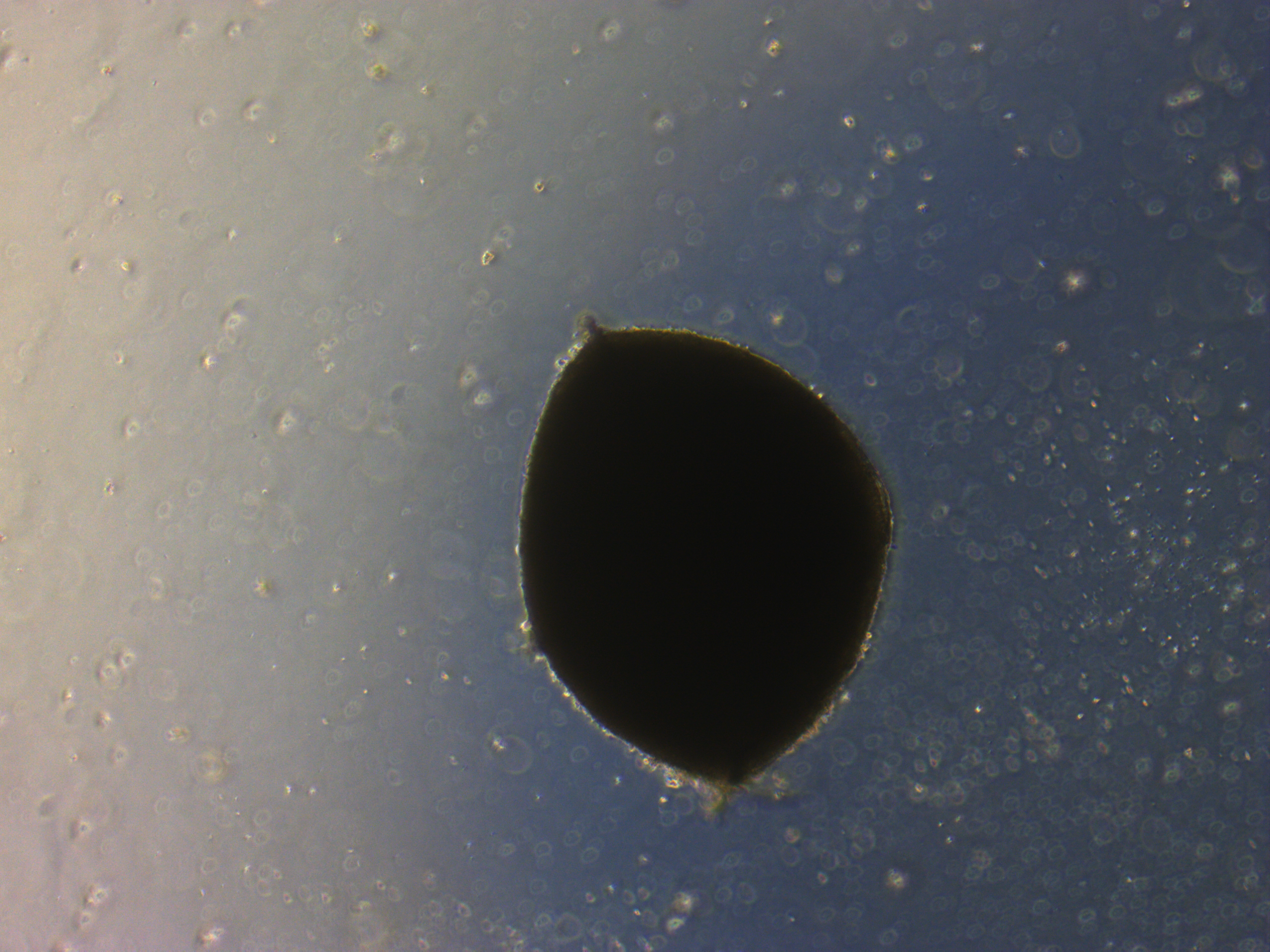

Supplement: Supplementary file 9 — Figure EV2 Source Data [file 44319_2025_595_MOESM9_ESM.zip › Figure EV2/EV2B/OSKM Day 15 3.tiff]

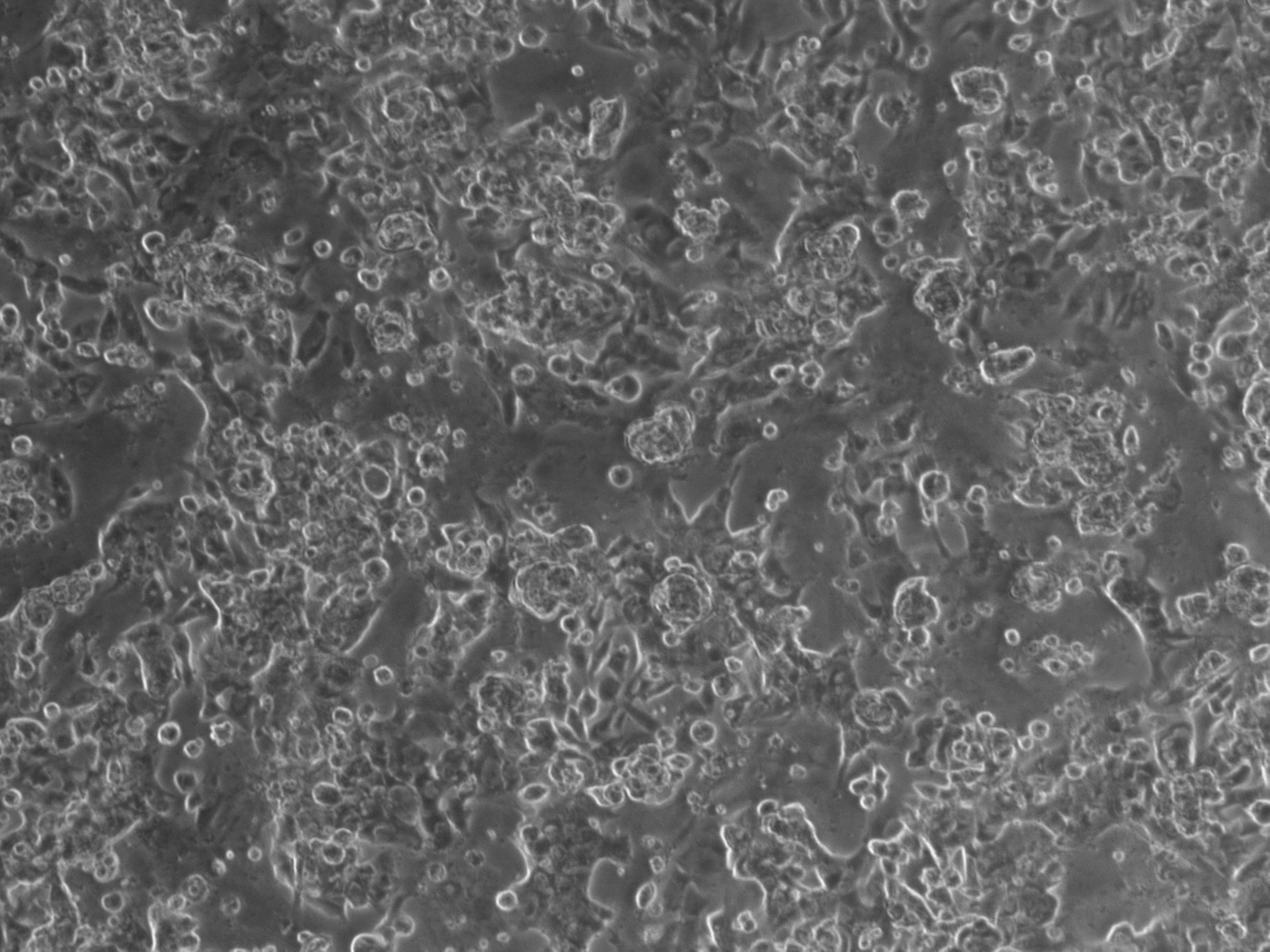

Supplement: Supplementary file 10 — Figure EV3 Source Data [file 44319_2025_595_MOESM10_ESM.zip › Figure EV3/EV3A/Empty_A_niPSC_PXGL.jpeg]

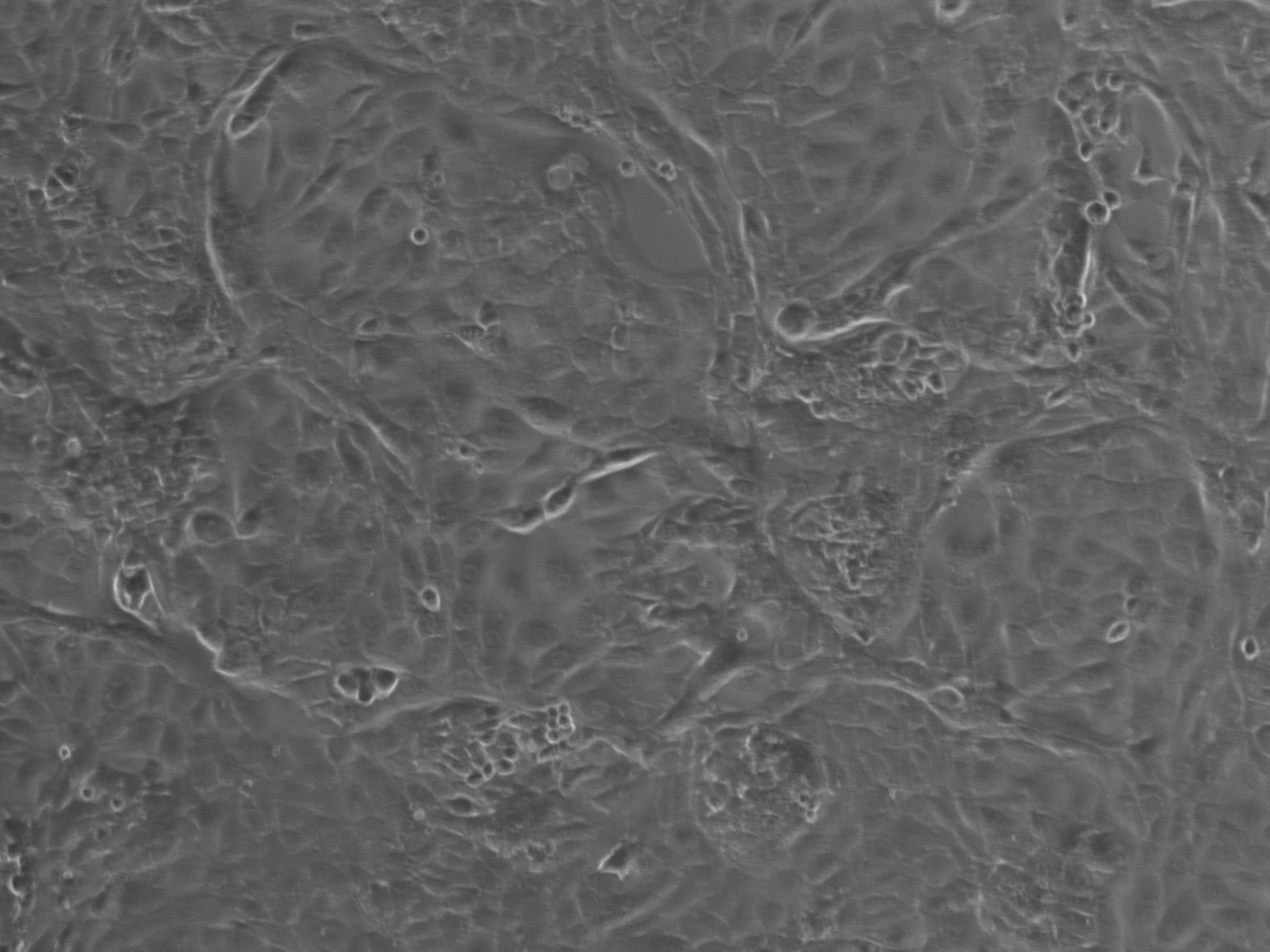

Supplement: Supplementary file 10 — Figure EV3 Source Data [file 44319_2025_595_MOESM10_ESM.zip › Figure EV3/EV3A/Empty_A_niPSC_TSC.jpeg]

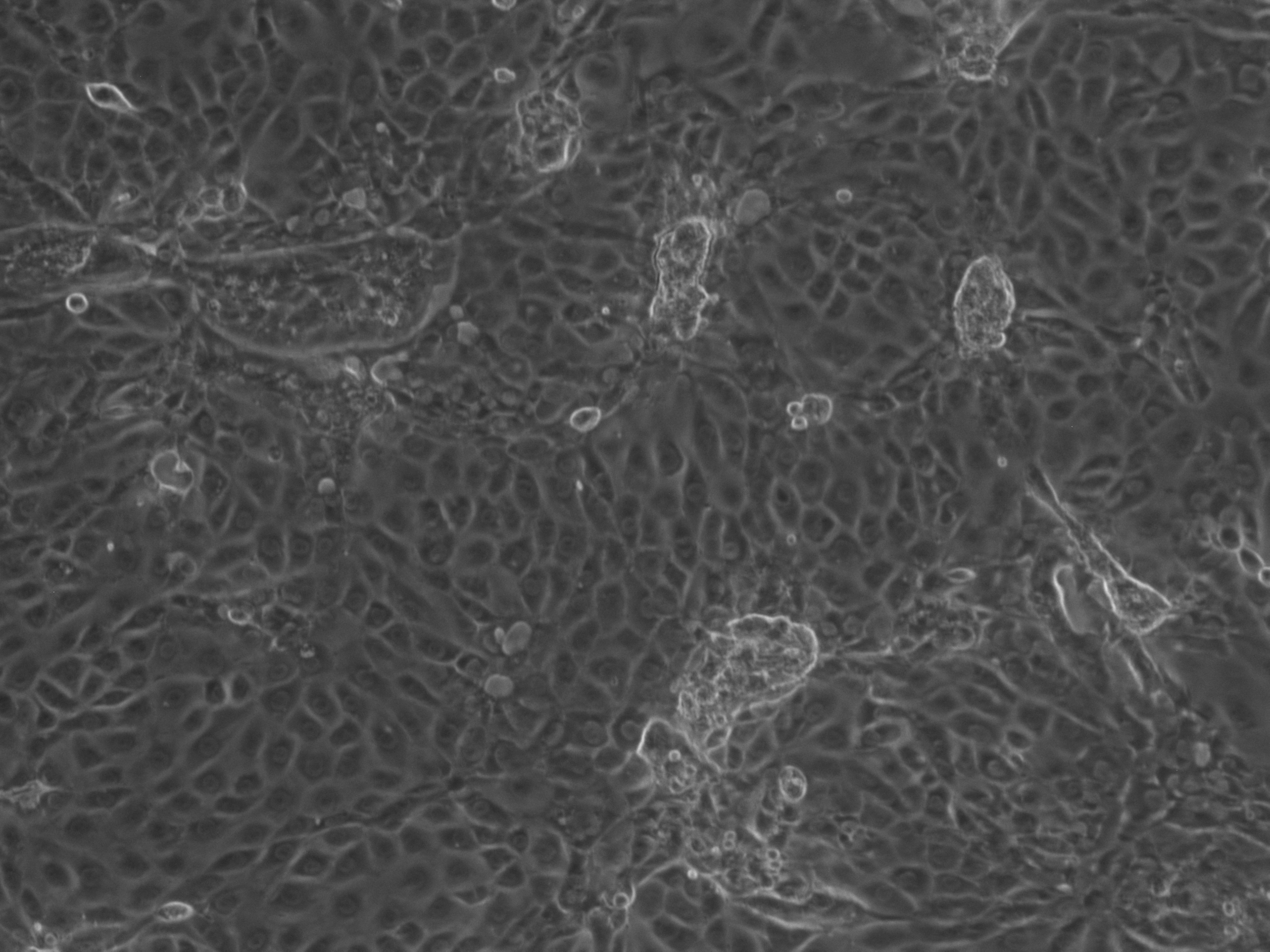

Supplement: Supplementary file 10 — Figure EV3 Source Data [file 44319_2025_595_MOESM10_ESM.zip › Figure EV3/EV3A/EMPTY_B-niPSC_TSC.jpeg]

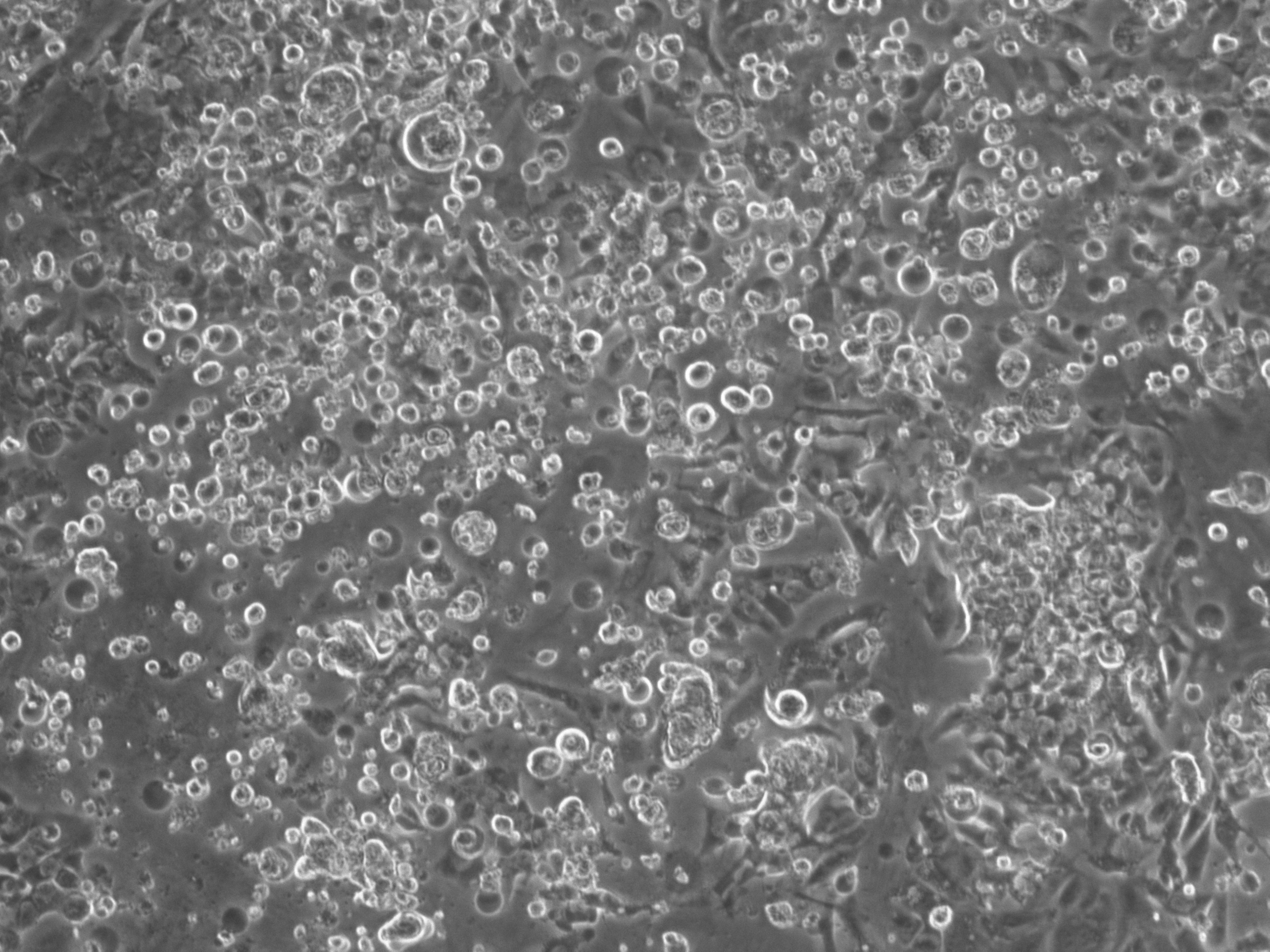

Supplement: Supplementary file 10 — Figure EV3 Source Data [file 44319_2025_595_MOESM10_ESM.zip › Figure EV3/EV3A/Empty_B_niPSC_PXGL.jpeg]

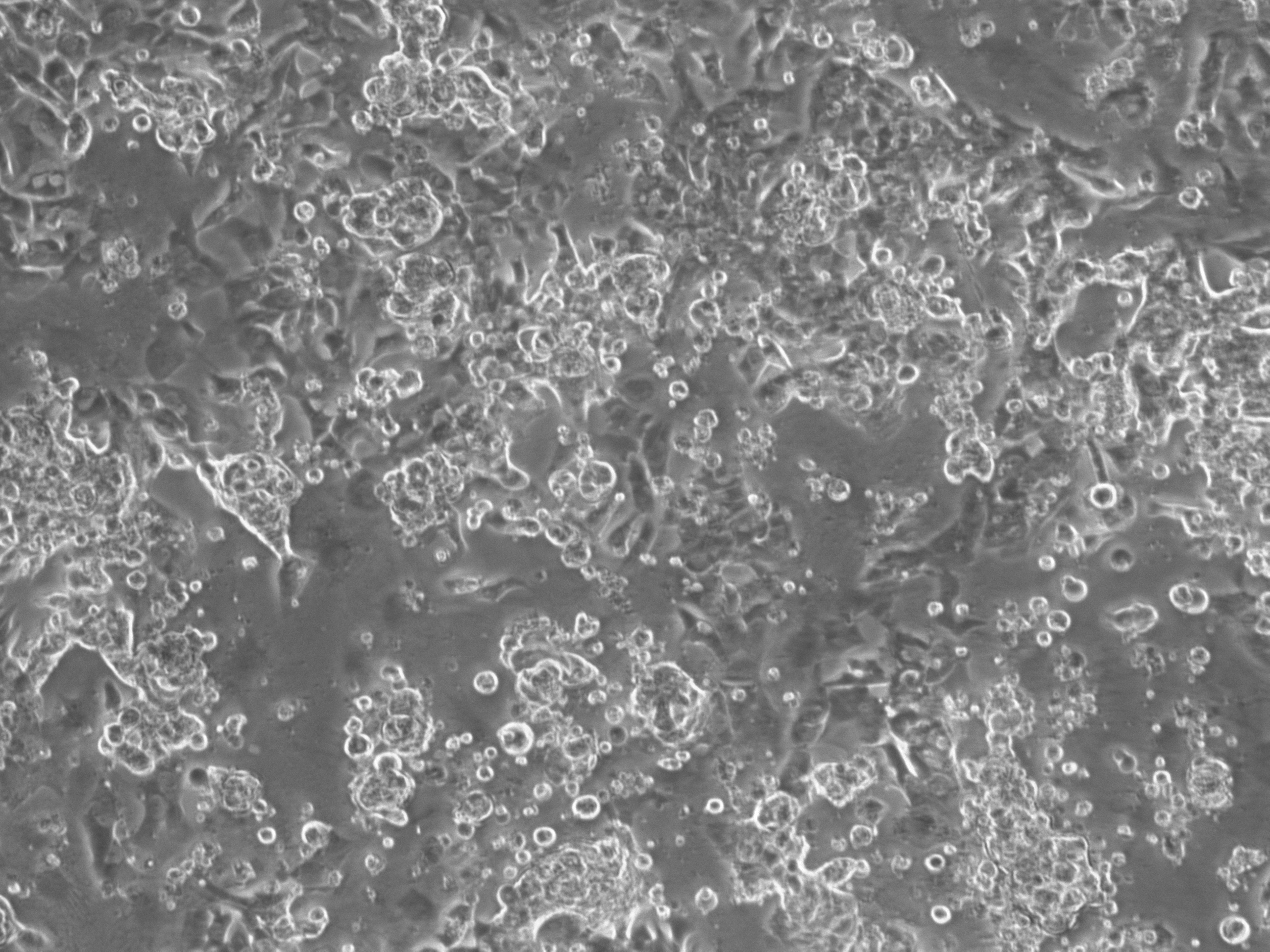

Supplement: Supplementary file 10 — Figure EV3 Source Data [file 44319_2025_595_MOESM10_ESM.zip › Figure EV3/EV3A/KLF7-niPSC_PXGL.jpeg]

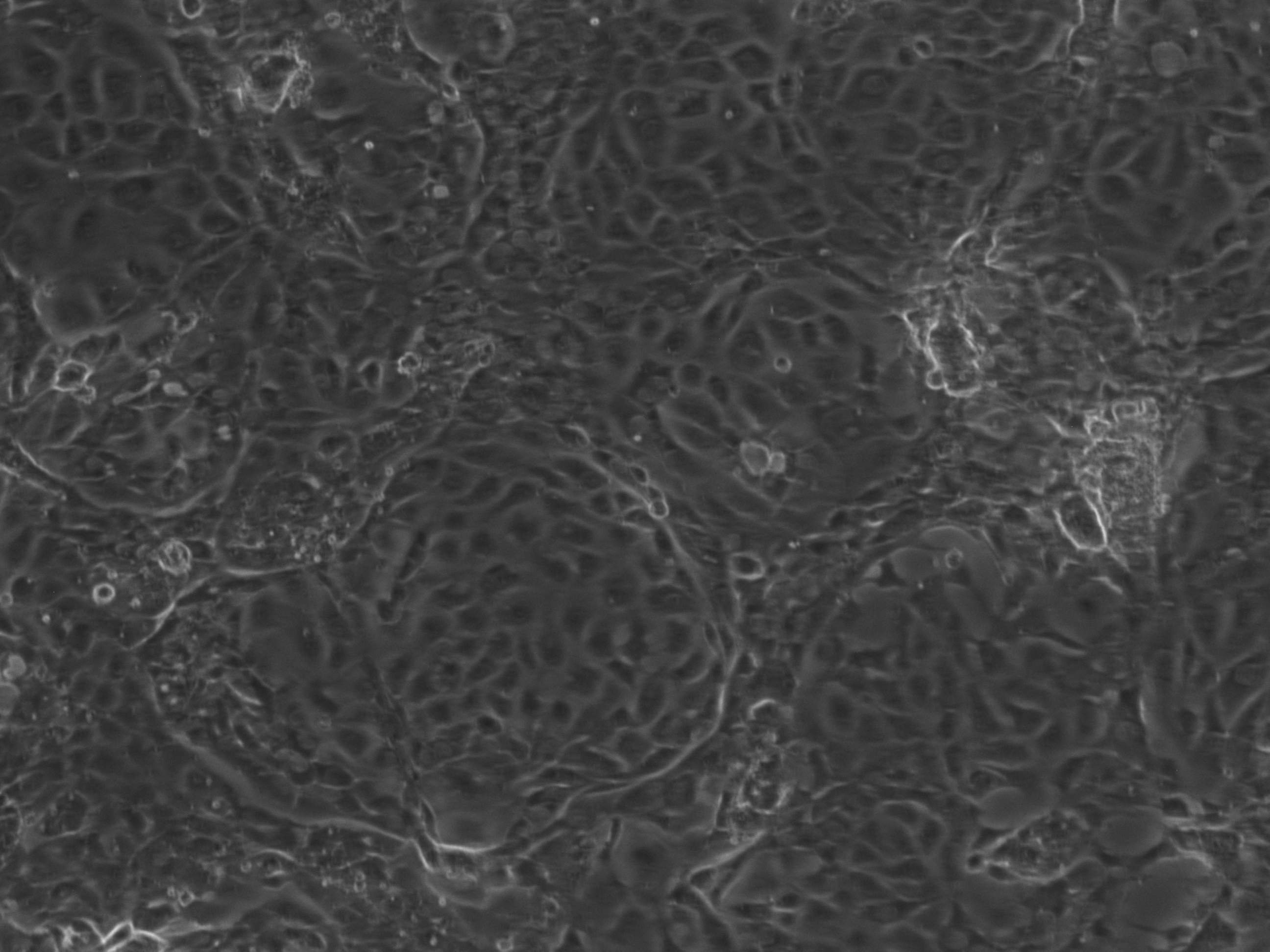

Supplement: Supplementary file 10 — Figure EV3 Source Data [file 44319_2025_595_MOESM10_ESM.zip › Figure EV3/EV3A/KLF7-niPSC_TSC.jpeg]
