# Supplementary material for: KLF7 is a general inducer of human pluripotency
Source: EMBO Rep. 2025 Oct 15;26(22):5372–96. doi: 10.1038/s44319-025-00595-2 (PMC12635360; doi:10.1038/s44319-025-00595-2)
Supplement: Supplementary file 13 — Expanded View Figures [file 44319_2025_595_MOESM13_ESM.pdf]

Expanded View Figures

Figure EV1. Characterisation of OSK7M iPSCs.

(**A**) Barplot showing the absolute expression, measured by RNAseq, of Krüppel-like factors KLF4 and KLF7 in conventional hESCs and hiPSCs. Data were obtained from (Choi et al, 2015; Dong et al, 2020; Giulitti et al, 2019; Jang et al, 2022; Liu et al, 2017; Theunissen et al, 2016; Wei et al, 2021; Zorzan et al, 2020, 2023). Mean +/− SD of at least 8 biological replicates is shown. (**B**) Brightfield images of iPSCs obtained at day 14 from fibroblasts reprogrammed by using 3 distinct reprogramming cocktails, OSKM, OSK7M and OSNL. Dashed circles indicate fully reprogrammed iPSCs colonies. Representative images of at least 3 independent experiments are shown. Scale bars: 100 µm. (**C**) Representative brightfield images of stabilised iPSCs cultures (10, 15 and 20 passages), obtained with OSKM and OSK7M reprogramming cocktails. Representative images of at least 3 independent experiments are shown. Scale bar: 100 µm. (**D**) Immunofluorescence images of pluripotency markers OCT4 and NANOG on stabilised iPSCs cultures (15 and 20 passages). Nuclei were stained with DAPI. Representative images of at least 3 independent experiments are shown. Scale bar: 30 µm. (**E**) Barplots showing expression measured by qPCR of primed pluripotency markers in stabilised iPSCs cultures, obtained with OSKM and OSK7M reprogramming cocktails. Bars= mean of 2 independent experiments, shown as dots. Source data are available online for this figure.

© The Author(s)

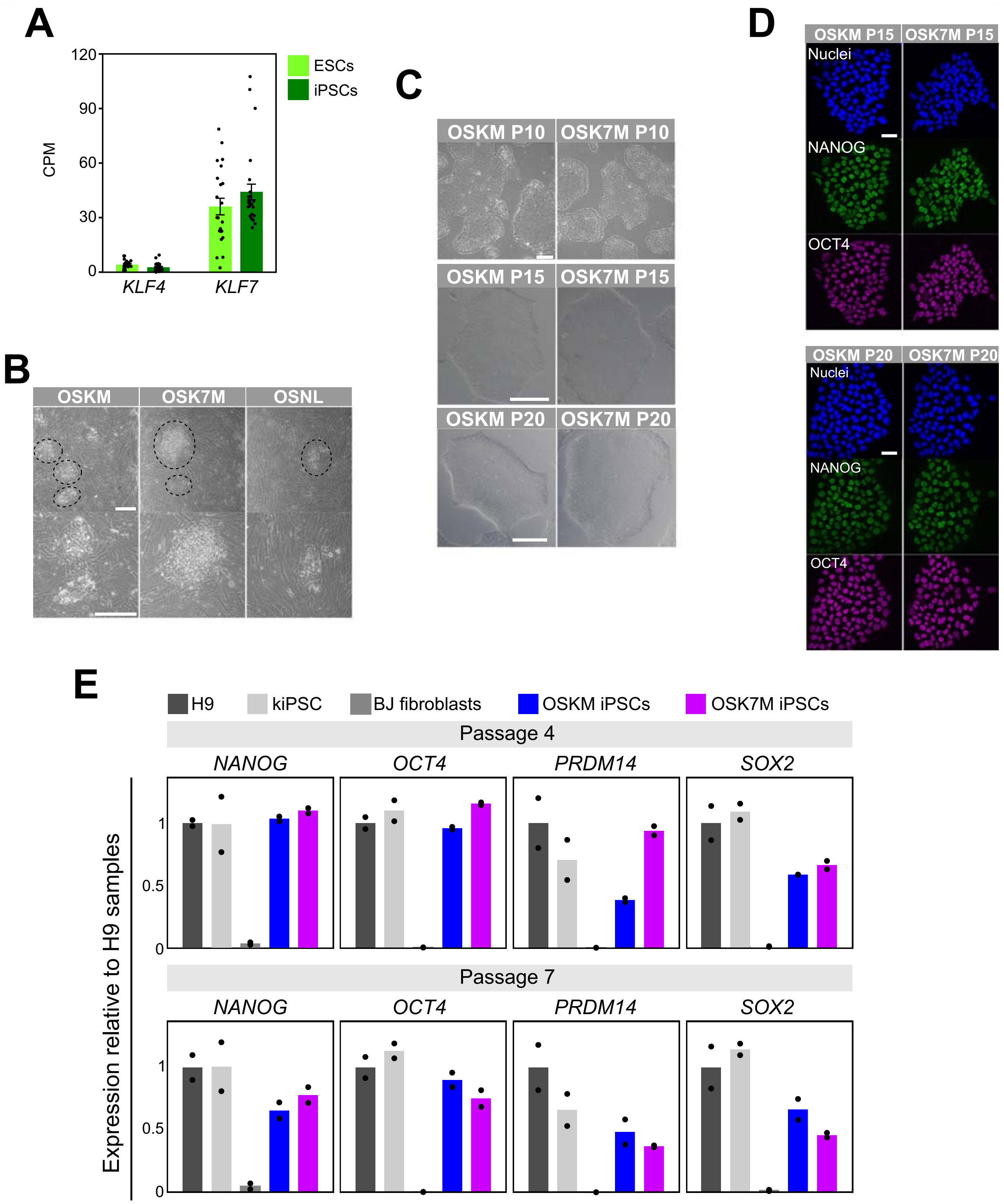
A
120
90
60
30
0
CPM
ESCs
iPSCs
KLF4
KLF7
B
OSKM
OSK7M
OSNL
C
OSKM P10
OSK7M P10
OSKM P15
OSK7M P15
OSKM P20
OSK7M P20
D
OSKM P15
OSK7M P15
Nuclei
NANOG
OCT4
OSKM P20
OSK7M P20
Nuclei
NANOG
OCT4
E
H9
kiPSC
BJ fibroblasts
OSKM iPSCs
OSK7M iPSCs
Passage 4
NANOG
OCT4
PRDM14
SOX2
Passage 7
NANOG
OCT4
PRDM14
SOX2
Expression relative to H9 samples
1
0.5
0

 © The Author(s)

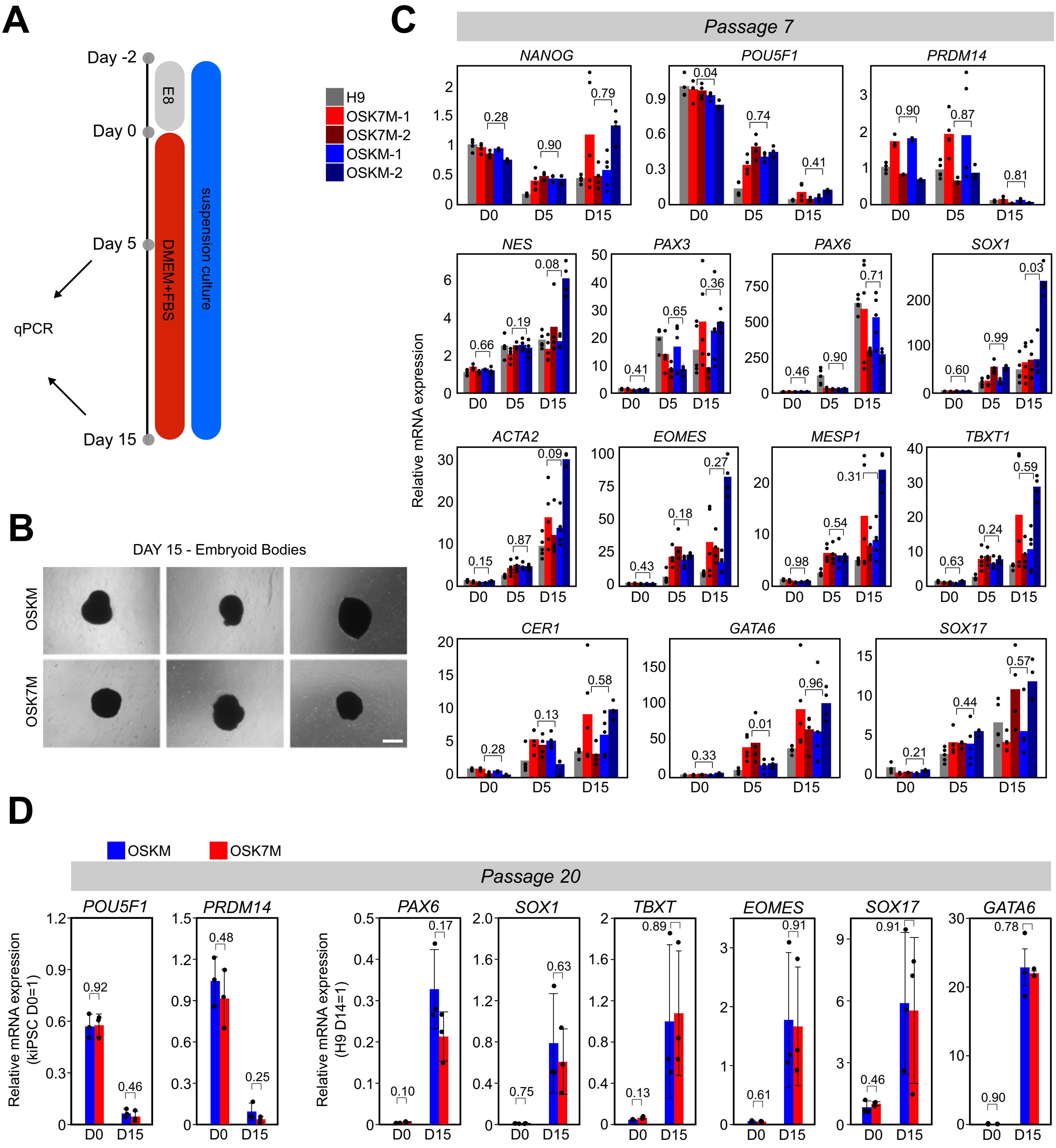

Figure EV2. Embryoid bodies differention of OSKM and OSK7M iPSCs.

(**A**) Schematic representation of experimental strategy used for Embryoid Bodies (EBs) differentiation of iPSCs obtained from reprogramming of fibroblasts with OSKM and OSK7M. (**B**) Representative bright field images of EBs obtained from OSKM and OSK7M iPSCs. Scale bar = 200 μm. (**C**, **D**) Barplots showing relative mRNA expression measured by qPCR of pluripotency and lineage markers in Embryoid bodies (EBs) obtained from differentiation of iPSCs generated by reprogramming with OSKM and OSK7M cocktails and stabilised in culture for 7 and 20 passages. kiPSC and H9 cell lines were used as positive control. Bars indicate the mean of at least 3 biological replicates, shown as dots. Unpaired two-tailed *t* test. Source data are available online for this figure.

© The Author(s)

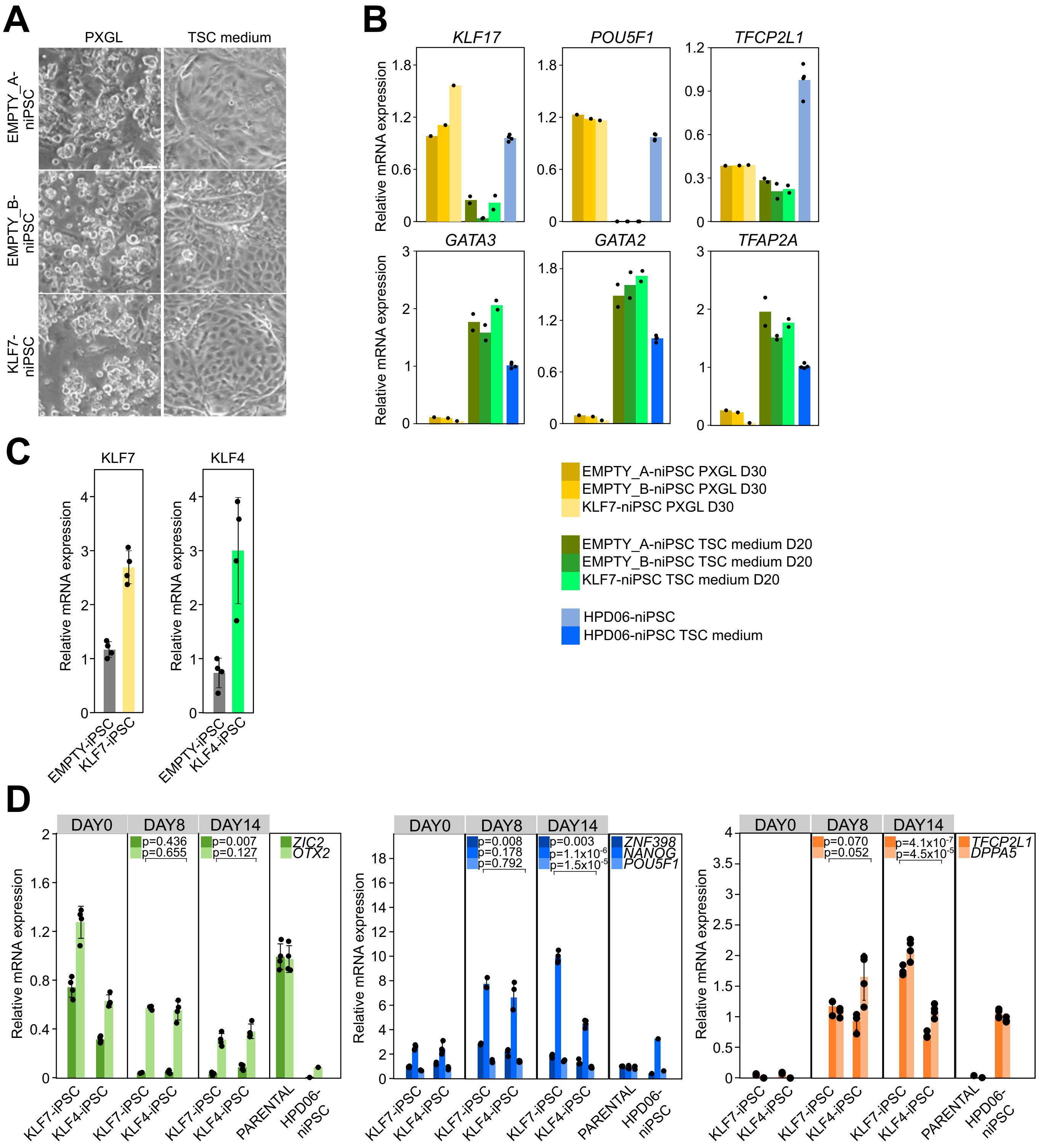
A
PXGL
TSC medium
EMPTY_A-niPSC
EMPTY_B-niPSC
KLF7-niPSC
B
KLF17
POU5F1
TFCP2L1
GATA3
GATA2
TFAP2A
Relative mRNA expression
EMPTY_A-niPSC PXGL D30
EMPTY_B-niPSC PXGL D30
KLF7-niPSC PXGL D30
EMPTY_A-niPSC TSC medium D20
EMPTY_B-niPSC TSC medium D20
KLF7-niPSC TSC medium D20
HPD06-niPSC
HPD06-niPSC TSC medium
C
KLF7
KLF4
Relative mRNA expression
EMPTY-iPSC
KLF7-iPSC
KLF4-iPSC
D
DAY0
DAY8
DAY14
p=0.436
p=0.655
p=0.007
p=0.127
ZIC2
OTX2
p=0.008
p=0.178
p=0.792
p=0.003
p=1.1x10-6
p=1.5x10-5
ZNF398
NANOG
POU5F1
p=0.070
p=0.052
p=4.1x10-7
p=4.5x10-5
TFCP2L1
DPPA5
KLF7-iPSC
KLF4-iPSC
PARENTAL
HPD06-niPSC

 © The Author(s)

Figure EV3. Testing KLF7 and KLF4 role in the induction of niPSCs.

(**A**) Representative brightfield images naive iPSCs (niPSC) obtained by chemical resetting of two independent kiPSCs expressing an empty vector (EMPTY_A-niPSC and EMPTY_B-niPSC) and of kiPSCs expressing KLF7 (KLF7-niPSC), and cultured in PXGL naive medium or in TSC medium for 20 days. Representative images of 2 biological replicates are shown. Scale bar: 100 µm. (**B**) Expression by qPCR of naive (KLF17, POU5F1 and TFCP2L1) and trophoblast (GATA3, GATA2 and TFAP2A) markers in the indicated naive iPSC lines cultured in TSC medium for 20 days, bars indicate the mean of 2 biological replicates. HPD06 naive iPSCs, HDP06 TSCs and EMPTY_A/B- or KLF7-niPSCs were used as controls. Bars indicate the mean of 1 or 4 biological replicates, shown as dots. (**C**) Barplot showing expression by qPCR of KLF7 and KLF4 in kiPSCs transfected with a plasmid carrying the KLF7 or the KLF4 transgenes respectively. Mean $+/-$ SD of 4 biological samples, shown as dots. (**D**) Expression by qPCR of conventional, shared and naive markers in kiPSC overexpressing an empty vector (EMPTY-iPSCs), the KLF7 transgene (KLF7-iPSCs) or the KLF4 transgene (KLF4-iPSCs) at day 0 and after 8 and 14 days of chemical resetting. Bars are the mean $+/-$ SD of 4 biological samples, shown as dots. Parental kiPSCs and naive iPSCs HPD06 were used as controls, $n = 1$ or 4 biological replicates, shown as dots. Source data are available online for this figure.

A

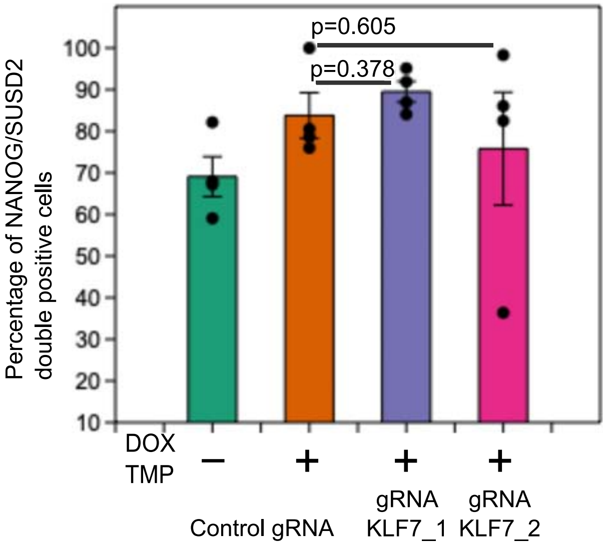

Figure EV4. Effect of KLF7 inhibition during chemical resetting.

(**A**) Quantification of the percentage of cells expressing both NANOG and SUSD2 after 14 days of chemical resetting with control and KLF7-CRISPRi cells. See Fig. 4F for representative images. Bars indicate means of 4 biological replicates. Unpaired two-tailed *t* test. Source data are available online for this figure.

 © The Author(s)

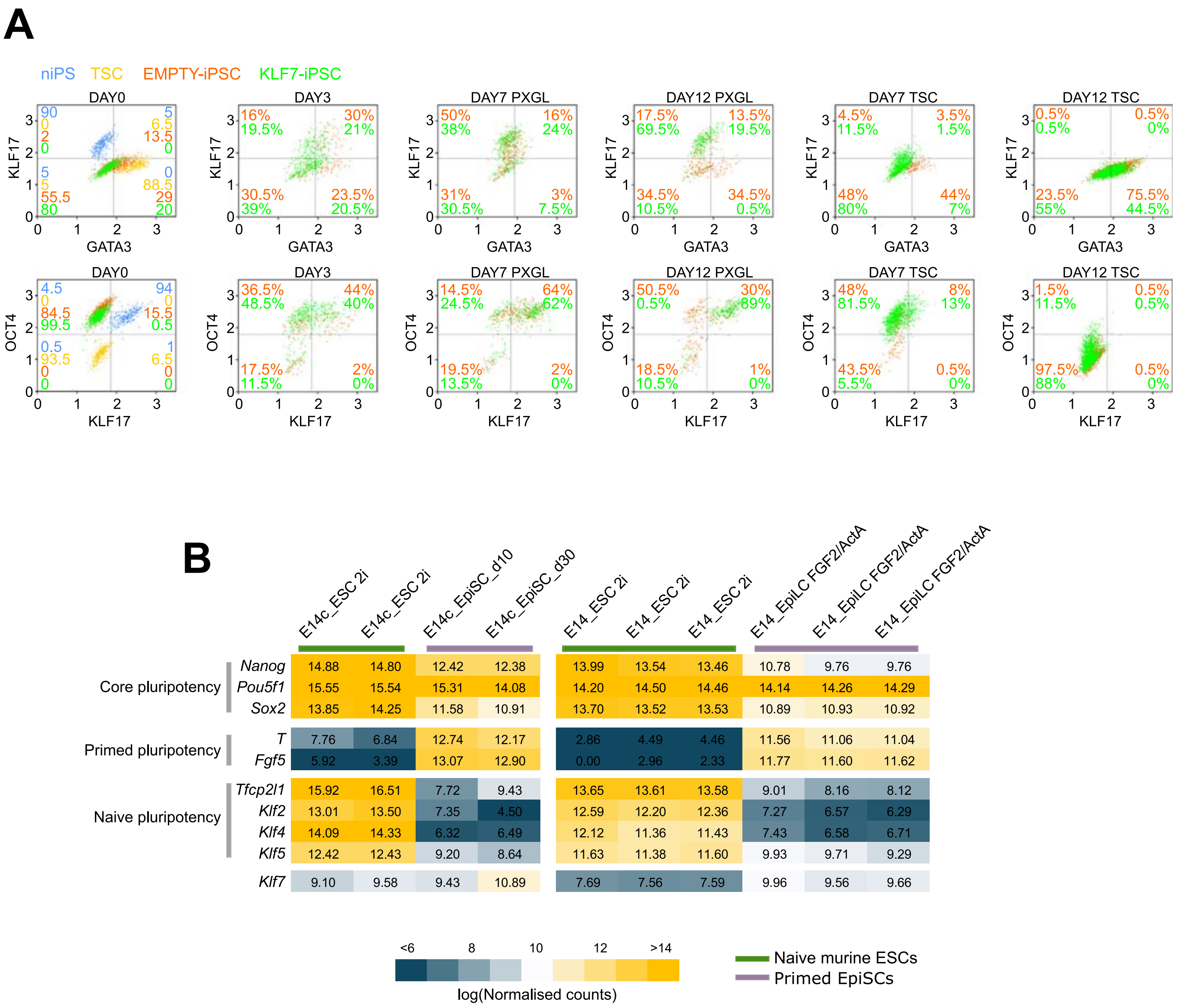

Figure EV5. Effect of KLF7 on chemical resetting and Klf7 expression analysis in murine PSCs.

(**A**) Scatter plots of quantification of immunofluorescence signals for OCT4, KLF17 and GATA3. Log10 Integrated Intensity signal obtained from Cell Profile software analysis is displayed (See Methods). The percentage of cells in each quadrant is the mean of 2 independent experiments. (**B**) Heatmap showing the expression levels of core, naive and primed pluripotency markers in mouse naive ESCs, primed EpiSCs and EpiLCs. Data from (Fan et al, 2020; Zhang et al, 2016). Source data are available online for this figure.

© The Author(s)
